# Crude Polysaccharide Extracts from Fresh and Dried *Cistanche deserticola* Attenuate Cyclophosphamide-Induced Immunosuppression in Mice

**DOI:** 10.3390/foods15213955

**Published:** 2026-11-04

**Authors:** Kun Lei, Haichun Hu, Yihan Lu, Haiyu Ji, Xiaoyu Jia, Wenjie Ding, Juan Yu, Bing Han

**Affiliations:** 1 Collaborative Innovation Center for Nutrition, Health, and Ecological Security of Food Resources in the Yellow River Basin, School of Life Sciences, Yantai University, Yantai 264005, China; leikun0104@163.com (K.L.); haichunhu@163.com (H.H.); luyih0410@163.com (Y.L.); yujuan14615@163.com (J.Y.); 2 Institute of Agricultural Products Preservation and Processing Technology (National Engineering Technology Research Center for Preservation of Agriculture Product), Tianjin Academy of Agricultural Sciences, Tianjin 300384, China; jiaxiaoyu@taas.ac.cn; 3 College of Food Science and Engineering, Tianjin University of Science and Technology, Tianjin 300457, China; dingwenjie1120@163.com

**Keywords:** *Cistanche deserticola* crude polysaccharide extracts, immunoregulatory function, drying treatment, gut microbiota, metabolomics

## Abstract

Primary drying is a routine operation for *Cistanche deserticola*, a traditional tonic whose polysaccharides serve as functional ingredients, yet whether it reshapes their fine structure and alters their biological effects remains unclear. Two crude polysaccharide extracts from fresh (FCDPE) and dried (DCDPE) *C. deserticola* were preliminarily characterised via gel-permeation chromatography, gas chromatography–mass spectrometry and infrared spectroscopy. Both extracts were predominantly composed of carbohydrates (total carbohydrate contents of 80.82% and 82.24%, respectively) and showed comparable main-peak apparent molecular weights; FCDPE retained a markedly higher arabinose proportion, whereas DCDPE was glucose-dominated and showed systematically smaller minor high-molecular-weight fractions. In cyclophosphamide-immunosuppressed mice, both preparations were associated with partially restored proportions of circulating CD3^+^ and CD19^+^ cells and with attenuated splenic and colonic injury relative to the model group. Microbiota profiling and faecal metabolomics revealed preparation-specific signatures: Lachnospiraceae were enriched in the FCDPE group, and Peptococcaceae were enriched in the DCDPE group, in contrast to the Enterobacteriaceae enrichment of the model group. Amino acids, oligopeptides and acylcarnitines were elevated in the FCDPE group, ceramides and succinate were decreased in both groups, together with the five lysophospholipid features shared by the two comparisons; and steroid biosynthesis was the most enriched pathway in both comparisons, although after Benjamini–Hochberg correction, it remained significant only in the DCDPE comparison (adjusted *p* = 0.029; adjusted *p* = 0.44 in the FCDPE comparison). Primary drying of *C. deserticola* therefore alters the composition of these crude extracts, and these compositional changes are accompanied by distinct microbial and metabolic signatures and by convergent, though partial, recovery of the measured immune readouts. These microbial and metabolic associations are exploratory and hypothesis-generating rather than confirmatory.

## 1. Introduction

*Cistanche deserticola* Y.C. Ma (Orobanchaceae), a perennial parasitic herb attached to the roots of the desert shrub *Haloxylon ammodendron*, has been consumed for centuries as a tonic material and used in traditional medicine, where it is regarded as the “ginseng of the desert” and is prescribed to invigorate kidney yang, moisten the intestine and relieve constipation [1,2]; these uses are also recorded in the national pharmacopoeia [3]. More than fifty constituents, including polysaccharides, phenylethanoid glycosides, iridoids and lignans, have been described in recent reviews [4]. *C. deserticola* polysaccharides, water-soluble glycans, mainly consisting of glucose, arabinose, galactose, rhamnose, mannose and xylose, possess diverse biological activities and are increasingly regarded as functional food ingredients; different preparation processes, moreover, yield polysaccharides with different structural features and bioactivities [5,6].

The biological activity of a polysaccharide is governed by its molecular weight, monosaccharide composition and glycosidic-linkage pattern, which together determine solubility, chain conformation and the epitopes recognised by microbial glycoside hydrolases and host immune receptors [7]. Because few glycosidic linkages are cleaved by mammalian digestive enzymes, most dietary glycans reach the colon largely intact, where they are fermented and thereby modify both the composition and the metabolic output of the resident community. Drying, the principal operation by which the fresh root is converted into a shelf-stable commodity, is far from inert: iridoids are degraded, Maillard reactions are driven towards 5-hydroxymethylfurfural, alkaloids are altered and the polysaccharide fraction is reorganised, as reflected in the pronounced colour difference between fresh and dried slices [8]. Studies comparing extraction and drying routes reach a consistent conclusion: the polysaccharide recovered is defined by the route taken, not by the plant alone [9]. Although partial structural information, including a galactoglucan backbone [10], two structurally distinct polymers [11] and characteristic acidic fractions [12], has been obtained, a direct comparison in which the fine structures of the fresh and dried polysaccharides are linked to microbial and immune readouts has not yet been conducted.

Dietary polysaccharides that escape digestion in the small intestine are fermented in the colon, where the resident microbiota—whose cell number is comparable to that of the host [13]—harvests energy from them and, in doing so, helps to maintain the mucosal barrier and to sustain immune homeostasis [14,15]. This arrangement is profoundly disturbed by alkylating chemotherapy, and cyclophosphamide (CTX) has been widely adopted to model this disturbance because it both ablates rapidly dividing immune cells and disrupts the mucus layer, which in turn compromises epithelial integrity and induces microbial dysbiosis [16]. Non-digestible carbohydrates have been reported to counteract these changes by promoting saccharolytic taxa, increasing short-chain fatty acid (SCFA) production and acting directly on immune cells, although these routes are difficult to separate experimentally [17]. For *C. deserticola*, shifts in microbial composition and SCFA profiles, together with dose- and host-state-dependent immunomodulation, have been described [18,19]. However, structural differences, community remodelling, metabolic output and immune recovery have not yet been examined together for *C. deserticola* polysaccharides, so the links between them remain assumed rather than tested.

In this study, the drying step used in primary processing was investigated by preparing two crude polysaccharide-containing extracts, designated FCDPE and DCDPE, from fresh and dried *C. deserticola* and by preliminarily characterising their apparent molecular-weight distributions, monosaccharide compositions and functional-group fingerprints by high-performance gel-permeation chromatography (HPGPC), gas chromatography–mass spectrometry (GC-MS) and Fourier-transform infrared spectroscopy (FT-IR). Subsequently, the gut microbiota, faecal metabolites, tissue morphology and lymphocyte subsets were evaluated in CTX-immunosuppressed mice by multi-omics and cytological assays. This study explores whether primary drying, a routine food-processing operation, alters the composition of these crude extracts and whether such compositional change is accompanied by changes in immune function and in the gut microbiota and metabolic profiles. Because the extracts are crude preparations, the polysaccharide fraction is treated as one of several constituents together with proteins, phenylethanoid glycosides, free sugars and minerals that may contribute to the observed effects.

## 2. Materials and Methods

### 2.1. Materials and Reagents

Fresh *C. deserticola* was provided by Xinjiang Yuanxiang Agricultural Technology Co., Ltd. (Hetian, China). The dried material was not purchased commercially: part of this fresh batch was dried in our laboratory by the procedure described below, and the remainder was processed in the fresh state, so the fresh (FCDPE) and dried (DCDPE) preparations differ only in the drying step. Specific-pathogen-free (SPF) female Kunming mice were provided by Shandong Pengyue Laboratory Animal Technology Co., Ltd. (Jinan, China). Erythrocyte-lysis reagent, phosphate-buffered saline, monosaccharide standards and T-series dextran calibration standards (T-3, T-10, T-70, T-100 and T-500) were obtained from Beijing Solarbio Science & Technology Co., Ltd. (Beijing, China).

For the dried material, fresh *C. deserticola* was cleaned, steamed until thoroughly cooked, cooled, and sliced into 3–5 mm pieces; the slices were dried in a hot-air circulating oven at 55 °C to a final moisture content ≤ 10.0%. Dried slices were sealed and stored at 2–8 °C in a dry environment protected from light, moisture and pests. Fresh and dried *C. deserticola* materials were immersed in deionised water at a solid-to-liquid ratio of 1:30 (g/mL) and extracted twice for 2 h in an 80 °C water bath; the two extracts were then pooled, filtered and concentrated. The concentrated extract was brought to a final ethanol concentration of 75% (*v*/*v*) by the dropwise addition of anhydrous ethanol to precipitate the polysaccharides; the resulting precipitate was collected, redissolved and lyophilised to yield FCDPE and DCDPE, respectively. Chemical analysis showed that both extracts were predominantly composed of carbohydrates, with total carbohydrate contents (phenol–sulfuric acid method) of 80.82 ± 4.26% in FCDPE and 82.24 ± 3.19% in DCDPE, whereas protein (Coomassie brilliant blue method; 5.6–6.0%), uronic acid (carbazole–sulfuric acid method; 7.3–8.9%) and reducing sugar (DNS method; 2.3–2.4%) were present only as minor constituents [20].

### 2.2. Molecular-Weight Distribution Assay

FCDPE, DCDPE, and T-series dextran standards were dissolved in deionised water at a concentration of 1 mg/mL, and all solutions were filtered through 0.45 μm aqueous microporous membranes. The molecular-weight distributions of the samples were determined by HPGPC on an Agilent 1200 series HPLC system (Agilent Technologies, Santa Clara, CA, USA). Separation was carried out on a TSKgel G4000PWxl aqueous size-exclusion column (7.8 mm × 300 mm, 10 μm particle size; Tosoh Bioscience, Tokyo, Japan) with refractive-index (RI) detection, and retention times and peak profiles were recorded simultaneously [21].

### 2.3. Monosaccharide Composition Assay

FCDPE and DCDPE were hydrolysed with 2 M trifluoroacetic acid (TFA) at 110 °C for 2 h; the hydrolysates were then evaporated to remove residual acid and redissolved in deionised water. After acetylation with acetic anhydride in pyridine, the resulting monosaccharide derivatives were extracted with dichloromethane and analysed on an Agilent 7000C GC-MS system (Agilent Technologies, Santa Clara, CA, USA) fitted with an HP-5 capillary column (30 m × 0.25 mm × 0.25 μm; Agilent Technologies, Santa Clara, CA, USA). Six monosaccharide standards, namely, rhamnose (Rha), arabinose (Ara), xylose (Xyl), mannose (Man), glucose (Glc) and galactose (Gal), were used for identification by comparison of retention times, and molar percentages were calculated by peak-area normalisation [22].

### 2.4. Functional Groups Assay

Lyophilized polysaccharide samples (0.7 mg) were ground with 150 mg of dried KBr and pressed into a transparent pellet. Spectra were recorded over 4000–400 cm^−1^ on a Bruker VECTOR-22 FT-IR spectrometer (Bruker BioSpin GmbH, Karlsruhe, Germany), and characteristic functional groups were assigned from the resulting bands [23].

### 2.5. Animal Model and Grouping

Forty female SPF Kunming mice (25 ± 2 g) were acclimatised for 1 week at 25 ± 2 °C and 50 ± 5% relative humidity under a 12 h light/dark cycle and then randomly assigned to a blank group (Blank), a model group (Model), an FCDPE group and a DCDPE group (10 mice per group). The FCDPE and DCDPE groups received the corresponding extract by oral gavage at 100 mg/kg/day (dose expressed on a crude-extract basis) from day 1 until sample collection on day 22, whereas the other two groups received an equivalent volume of saline. On days 8–10, all groups except Blank received intraperitoneal CTX (60 mg/kg) once daily to induce immunosuppression; the design was therefore prophylactic, with extract administration continuing during and after CTX exposure. On day 22, peripheral blood, faeces, spleen and colon were collected for further analysis [24]. Two animals per group with abnormal body-weight trajectories were excluded from the flow-cytometric analysis, which was therefore based on eight animals per group. The 16S rRNA sequencing and faecal metabolomics analyses were performed on faecal samples from three animals per group whose peripheral CD3^+^ and CD19^+^ proportions were closest to the group mean, so the profiled samples represent the central tendency of each group rather than its most extreme responders. 

### 2.6. Histopathological H&E Staining Assay

Tissue samples from the spleen and colon were immersed in 4% paraformaldehyde for fixation, passed through a series of graded ethanol solutions for dehydration, and subsequently embedded in paraffin blocks, from which 5 μm sections were cut and subjected to haematoxylin–eosin staining. Briefly, the sections were deparaffinised with xylene, rehydrated through a descending ethanol series, treated with Harris haematoxylin for 5 min, differentiated in 1% acid alcohol, blued in dilute ammonia solution and counterstained with eosin for 2 min. After dehydration and clearing in xylene, the stained sections were finally coverslipped with neutral balsam. Stained sections were imaged on a Flea-n cell imaging system (LC, Shanghai Leite Technology Co., Ltd., Shanghai, China) equipped with a 3.2 MP high-sensitivity CMOS camera [25]. The histological evaluation was a preliminary, qualitative observation of morphological changes, without blinded semi-quantitative scoring or morphometry; representative sections from each group were examined at both magnifications.

### 2.7. Flow-Cytometric Assay

Peripheral blood was collected and the erythrocytes were lysed; the remaining cells were incubated in the dark with CD3-FITC and CD19-PE antibodies for 30 min at 4 °C, washed, resuspended and filtered through nylon mesh. All samples were then acquired on a CytoFLEX flow cytometer (Beckman Coulter, Brea, CA, USA), and the proportions of CD19^+^ B cells and CD3^+^ T cells were determined with CytExpert software (v2.5) [26]. Briefly, the acquired events were first gated on forward versus side scatter to exclude debris and cell aggregates, and the gated single cells were then displayed as CD3 versus CD19 dot plots, in which the lymphocyte populations were assigned to quadrants Q2-UL (CD3^−^CD19^+^, B cells) and Q2-LR (CD3^+^CD19^−^, T cells). Fluorescence-minus-one (FMO) controls were designed and acquired, and the quadrant boundaries in the CD3/CD19 dot plots were positioned on the basis of these FMO controls rather than set arbitrarily, so the subset proportions reported here rest on a defined positivity threshold.

### 2.8. 16S rRNA Sequencing of Gut Microbiota

Total microbial DNA was extracted from faecal samples using a QIAamp PowerFecal Pro DNA Kit (Qiagen, Hilden, Germany) according to the manufacturer’s instructions. The V3–V4 region of the 16S rRNA gene was amplified with the primer pair 338F (5′-ACTCCTACGGGAGGCAGCAG-3′) and 806R (5′-GGACTACHVGGGTWTCTAAT-3′), which provides broad bacterial coverage. Amplicons were purified, quantified with a QuantiFluor-ST fluorometer (Promega, Madison, WI, USA), indexed, and paired-end-sequenced on an Illumina MiSeq platform (Illumina, San Diego, CA, USA). Reads that passed quality control were clustered into operational taxonomic units (OTUs), and Venn, rarefaction, taxonomic-composition and linear discriminant analysis effect size (LEfSe) analyses were performed with QIIME2 (v2022.2) using Greengenes 13_8 as the reference database; LEfSe was run at an LDA score threshold of 4.0, with *p* < 0.05 considered significant [27].

### 2.9. Faecal Metabolomics Assay

Faecal samples were ground in liquid nitrogen, accurately weighed, and extracted with 80% methanol; the supernatants obtained after centrifugation were separated on a Dionex Ultimate 3000 UHPLC system (Thermo Fisher Scientific, Germering, Germany) coupled to a C18 column (2.1 mm × 100 mm, 1.7 μm; Phenomenex, Torrance, CA, USA) and analysed on a Q Exactive mass spectrometer (Thermo Fisher Scientific, Bremen, Germany) for untargeted profiling, following established global metabolic profiling workflows. Metabolites were identified and quantified, and differential metabolites were selected by combined multivariate and univariate statistics based on variable importance in projection (VIP), *p* value and fold change, with differential features defined by VIP > 1, unadjusted *p* < 0.05 and FC ≥ 1.5 or ≤0.67, followed by KEGG pathway enrichment [28].

### 2.10. Statistical Analysis

All data are expressed as means ± standard deviation (SD), and lymphocyte proportions were compared by one-way analysis of variance (ANOVA) followed by Tukey’s HSD post hoc test using SPSS 26.0 (IBM Corp., Armonk, NY, USA), with *p* < 0.05 considered statistically significant. The comparisons of primary interest were those between each extract group and the model group; a formal statistical contrast between the FCDPE and DCDPE groups was not performed, and the two preparations are therefore compared descriptively rather than in terms of differential efficacy. In the flow-cytometric figures, * denotes a significant difference versus the blank group and # a significant difference versus the model group. Figures were generated with instrument software and Microsoft Excel 2021 (Microsoft Corp., Redmond, WA, USA).

## 3. Results

### 3.1. Molecular-Weight Distributions of FCDPE and DCDPE

**Figure 1:**
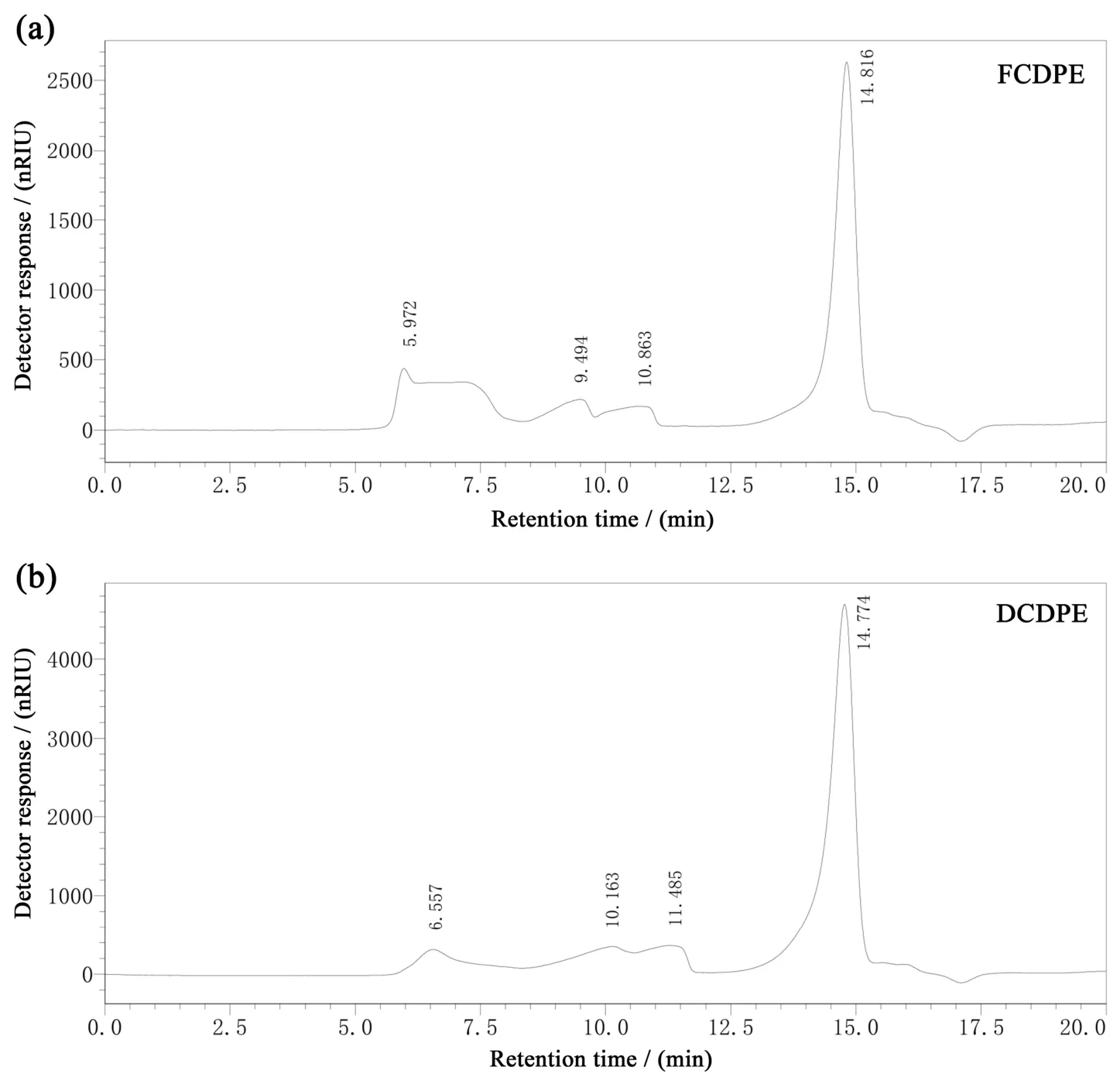
HPGPC chromatograms of FCDPE (**a**) and DCDPE (**b**).

The molecular weight distributions of FCDPE and DCDPE are shown in Figure 1; neither preparation appeared as a single homogeneous component in the HPGPC chromatogram, and their elution profiles differed appreciably. On the basis of the dextran calibration curve (lgMw = −0.4628·RT + 11.0476, R^2^ = 0.9984), the main components of the two preparations had comparable average molecular weights, approximately 1.55 × 10^4^ Da for FCDPE (retention time 14.816 min) and 1.62 × 10^4^ Da for DCDPE (retention time 14.774 min). In addition to the main peak, both preparations exhibited three minor high-molecular-weight peaks: for FCDPE, the peaks at 5.972, 9.494 and 10.863 min corresponded to apparent molecular weights of approximately 1.92 × 10^8^, 4.51 × 10^6^ and 1.05 × 10^6^ Da, respectively; for DCDPE, the peaks at 6.557, 10.163 and 11.485 min corresponded to apparent molecular weights of approximately 1.03 × 10^8^, 2.21 × 10^6^ and 5.40 × 10^5^ Da, respectively. These results indicate that broad molecular-weight distributions were exhibited by both extracts, and it was noted that the G4000PWxl column had a nominal exclusion limit of approximately 3 × 10^5^ Da while the adopted dextran standards (T-3 to T-500) did not cover the apparent molecular weights of the three minor high-molecular-weight peaks, which were positioned beyond the fractionation range near the column void volume where retention was no longer governed by molecular size. The reported values were extrapolated dextran-equivalent apparent estimates with limited quantitative reliability, which were included only for completeness and were not interpreted as true molecular masses, so the shift in these peaks between the two preparations was described as a change in the excluded fraction rather than a change in molecular weight. Systematically smaller minor high-molecular-weight peaks were observed after drying, which was consistent with degradation or altered extractability of these fractions although hydrolysis was not demonstrated by the present data alone.

### 3.2. Chemical and Monosaccharide Compositions of FCDPE and DCDPE

Because both extracts contained about 80–82% total carbohydrate (Section 2.1), the carbohydrate fraction accounted for 80.82% and 82.24% of the administered crude-extract mass in the FCDPE and DCDPE groups, respectively (a relative difference of 1.8%), so the two groups received essentially the same amount of carbohydrate per unit body weight and the biological comparison was not confounded by a difference in carbohydrate dose. The remaining constituents were present in similarly minor proportions in the two extracts, so the compositional differences described below are more likely to reflect the nature of the carbohydrate fraction than differences in overall purity.

**Figure 2:**
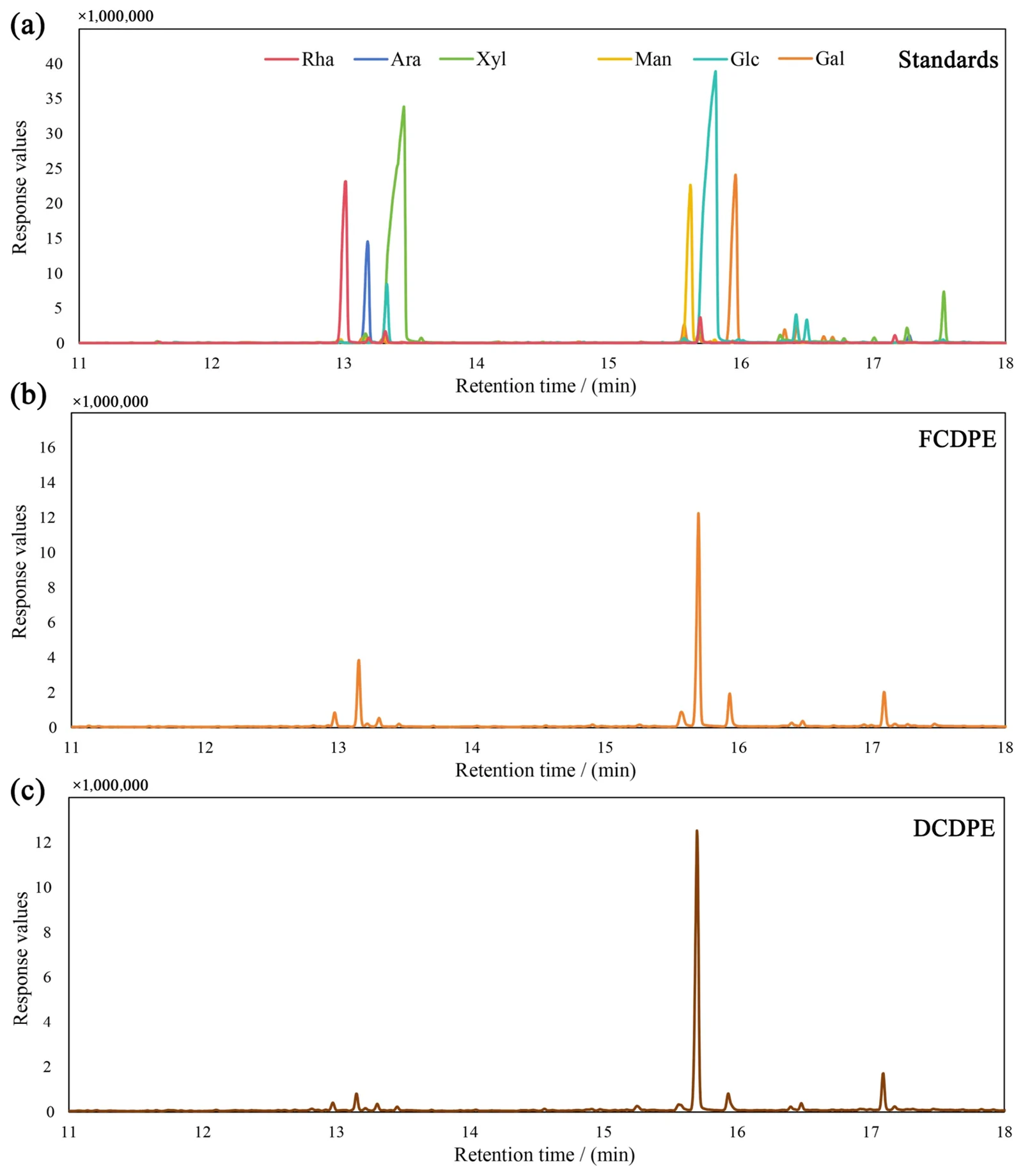
GC-MS chromatograms of monosaccharide standards (**a**), FCDPE (**b**), and DCDPE (**c**).

Both FCDPE and DCDPE contained the same six monosaccharides (Figure 2), although their molar proportions differed markedly. DCDPE was dominated by glucose (74.87%), with galactose (6.83%), arabinose (6.54%), mannose (4.22%), rhamnose (3.81%) and xylose (3.72%) as minor components, a composition consistent with a glucan-rich extract. Relative to DCDPE, FCDPE contained less glucose (54.74%) and approximately threefold more arabinose (20.24%); galactose, mannose and rhamnose were also higher (9.59%, 6.58% and 5.02%, respectively), whereas xylose was essentially unchanged (3.83%). Arabinose is characteristic of arabinan- and arabinogalactan-type polysaccharides; the lower arabinose proportion in the dried extract is consistent with loss, degradation or reduced extractability of arabinose-rich material, but compositional data alone cannot localise such changes to side chains of a common backbone, and the interpretation of bioactivity differences remains tentative [11].

### 3.3. FT-IR Spectral Features of FCDPE and DCDPE

**Figure 3:**
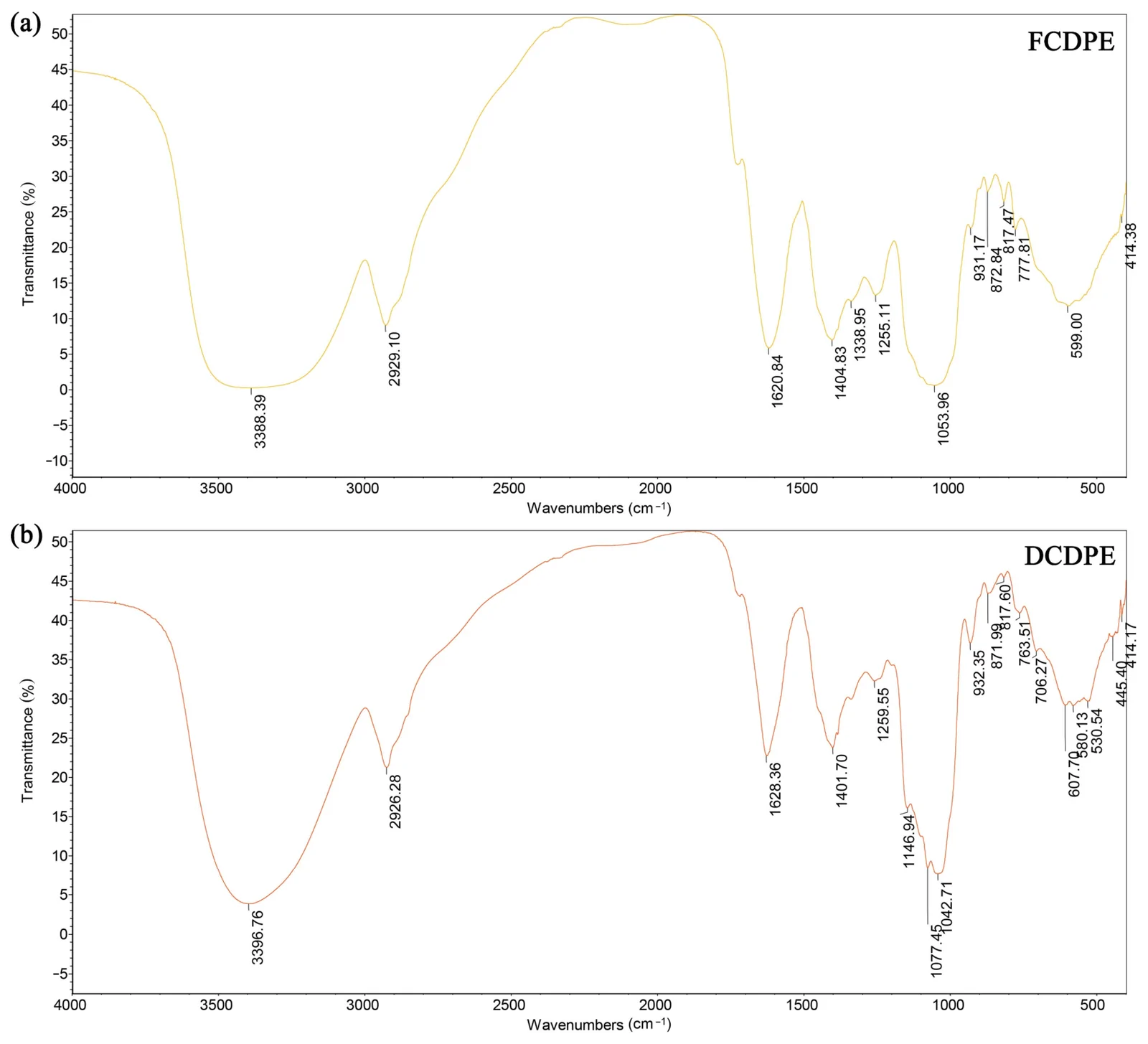
FT-IR spectroscopic characterisation and comparison of FCDPE (**a**) and DCDPE (**b**).

The FT-IR spectra of FCDPE and DCDPE shared several characteristic absorption bands typical of carbohydrates (Figure 3). Both samples exhibited broad, strong absorption centred at 3388.39 cm^−1^ (FCDPE) and 3396.76 cm^−1^ (DCDPE), assigned to O–H stretching vibrations involved in intra- and intermolecular hydrogen bonding. The weak bands at 2929.10 cm^−1^ (FCDPE) and 2926.28 cm^−1^ (DCDPE) were attributable to C–H stretching vibrations. The absorptions at 1620.84 cm^−1^ (FCDPE) and 1628.36 cm^−1^ (DCDPE) overlapped the O–H bending vibration of bound water and the amide I band [11,29]. The bands at 1404.83 cm^−1^ (FCDPE) and 1401.70 cm^−1^ (DCDPE) corresponded to C–H bending vibrations, and the prominent absorptions assigned to the C–O–C and C–O–H stretching vibrations of the sugar-ring skeleton occurred at 1053.96 cm^−1^ in FCDPE and at 1042.71/1077.45 cm^−1^ in DCDPE [29].

In the 900–750 cm^−1^ region, the bands at 931.17 cm^−1^ (FCDPE) and 932.35 cm^−1^ (DCDPE) were assigned to the asymmetric ring-stretching vibration of the pyranose ring, and the bands at 872.84 and 817.47 cm^−1^ in FCDPE and at 871.98 and 817.60 cm^−1^ in DCDPE were attributed to furanose ring-skeletal vibrations [30]. Within this region, the bands at 777.81 cm^−1^ (FCDPE) and 763.51 cm^−1^ (DCDPE) were the only clearly shifted features, with a displacement of approximately 14 cm^−1^ that may reflect differences in substitution pattern or in hydrogen-bonding environment between the two samples.

### 3.4. Histopathological Changes (H&E)

**Figure 4:**
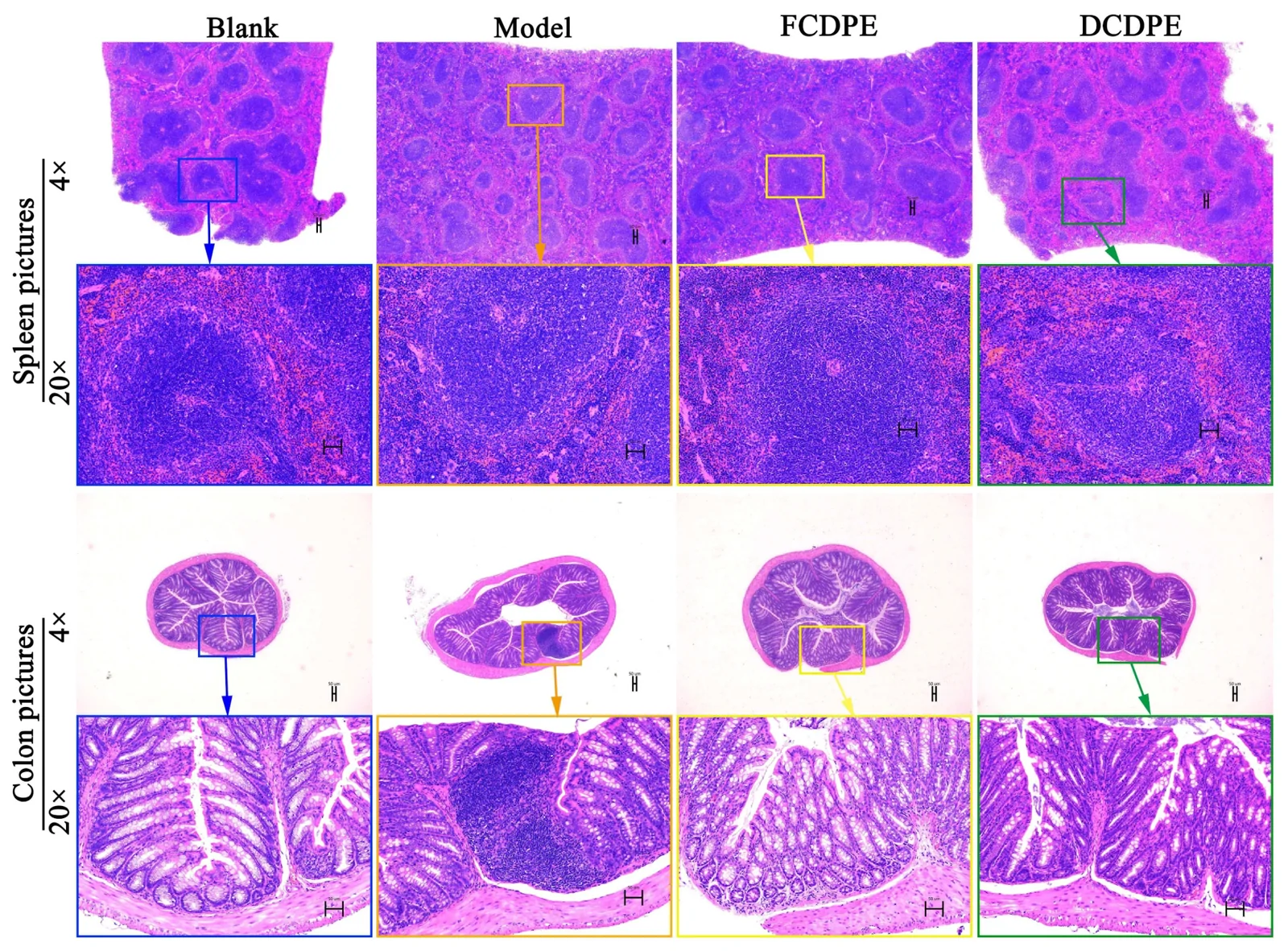
H&E staining of the spleen (**upper**) and colon (**lower**) in each group.

Histopathological changes in the spleen were assessed by H&E staining at both low (4×) and high (20×) magnification (Figure 4, upper panels). In the blank group, the spleen displayed a well-organised architecture, in which the white pulp and red pulp were clearly demarcated, and the splenic nodules (lymphoid follicles) exhibited distinct boundaries with discernible germinal centres and compactly arranged lymphocytes. In the model group, the splenic architecture appeared disorganised, with loss of clear demarcation between the white pulp and red pulp, indistinct boundaries of the splenic nodules, a lower density of lymphocytes and less clearly defined germinal centres. Compared with the model group, the FCDPE and DCDPE groups showed a more regular splenic architecture, with clearer boundaries of the splenic nodules and a higher lymphocyte density, although splenic morphology was not fully restored to that of the blank group [31].

The colonic histomorphology was evaluated by H&E staining at both low (4×) and high (20×) magnification (Figure 4, lower panels). In the blank group, the colonic mucosa showed an intact epithelium, with regularly arranged crypts, abundant goblet cells and a lamina propria free of obvious inflammatory cell infiltration. In the model group, the colonic mucosa exhibited evident damage, including disruption of the crypt architecture, a reduction in goblet cells, inflammatory cell infiltration in the lamina propria, and submucosal oedema. Compared with the model group, the FCDPE and DCDPE groups displayed a more intact mucosal architecture, with more regularly arranged crypts, more abundant goblet cells and attenuated inflammatory cell infiltration, indicating attenuated colonic mucosal injury [32]. This assessment is qualitative; no blinded semi-quantitative scoring or morphometric measurements (e.g., crypt depth, goblet-cell counts or inflammatory scores) were performed, and this limitation is acknowledged in the Discussion.

### 3.5. Peripheral Lymphocyte Subsets

**Figure 5:**
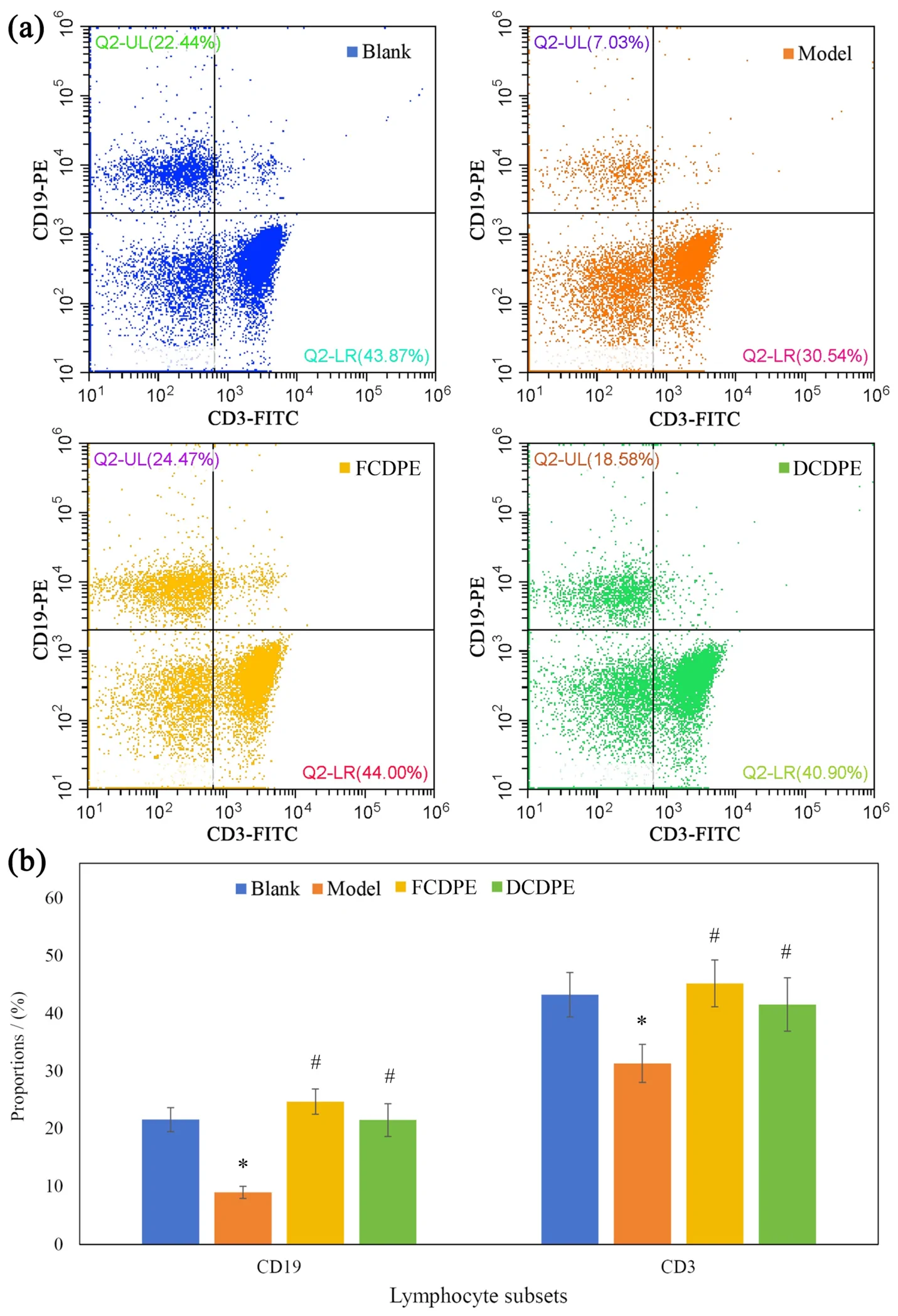
Flow-cytometric scatter plots (**a**) and proportions (**b**) of peripheral CD3/CD19 cells in each group. Quadrants: Q2-UL, CD3^−^CD19^+^ (B lymphocytes); Q2-LR, CD3^+^CD19^−^ (T lymphocytes). Values are relative proportions (%) of gated events, not absolute counts; bars show means ± SD (*n* = 8) and the per-animal values are available from the corresponding authors on request. Note: * *p* < 0.05 versus the blank group; # *p* < 0.05 versus the model group.

To assess the effects of the two *C. deserticola* extracts on peripheral lymphocyte subsets, whole blood was stained with anti-CD3-FITC and anti-CD19-PE and analysed by flow cytometry; representative events, resolved into quadrants Q2-UL (CD3^−^CD19^+^, B cells) and Q2-LR (CD3^+^CD19^−^, T cells), are shown in Figure 5. In the representative scatter plots (Figure 5a), the cells form well-defined clusters with clearly separated populations in the expected quadrants, indicating that the gating strategy resolved the two subsets. As summarised in Figure 5b, relative to the blank group, the model group showed markedly lower CD19^+^ and CD3^+^ proportions (both *p* < 0.05), whereas both FCDPE and DCDPE showed higher CD19^+^ and CD3^+^ proportions than the model group (*p* < 0.05). Taken together, partial recovery of circulating T and B lymphocyte subset proportions was observed in immunosuppressed mice receiving either extract preparation; the reported values are relative proportions (%) of gated events rather than absolute counts. These data nevertheless describe subset proportions only and therefore do not permit conclusions about the underlying mechanisms [33].

### 3.6. Taxonomic Composition of Gut Microbiota

**Figure 6:**
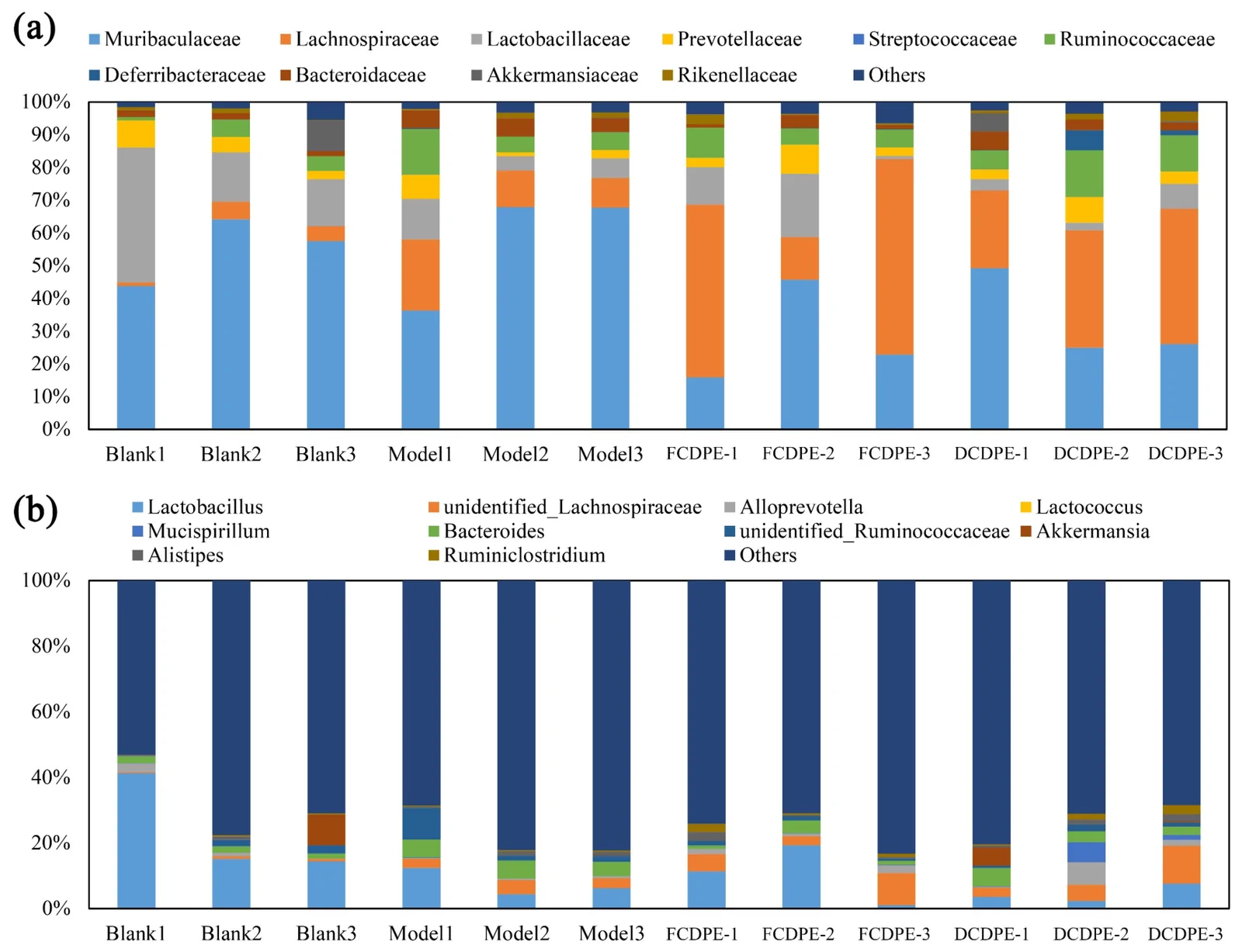
Relative abundance of the top 10 taxa at the family (**a**) and genus (**b**) levels in each group (*n* = 3).

The distribution of dominant bacterial families, determined by 16S rRNA gene amplicon sequencing, was summarised as stacked bar plots (Figure 6a), reflecting the overall family-level composition of the gut microbiota. The most abundant families across all samples were Muribaculaceae, Lachnospiraceae, Lactobacillaceae, Prevotellaceae, Streptococcaceae and Ruminococcaceae. The family-level composition differed among the groups. At the genus level, the relative abundances of the dominant genera were displayed as stacked bar plots (Figure 6b). The most abundant genera across all samples were *Lactobacillus*, unidentified_Lachnospiraceae, *Alloprevotella*, *Lactococcus*, *Mucispirillum* and *Bacteroides*. The genus-level composition also differed among the groups; the detailed differences are addressed in the differential analysis below.

**Figure 7:**
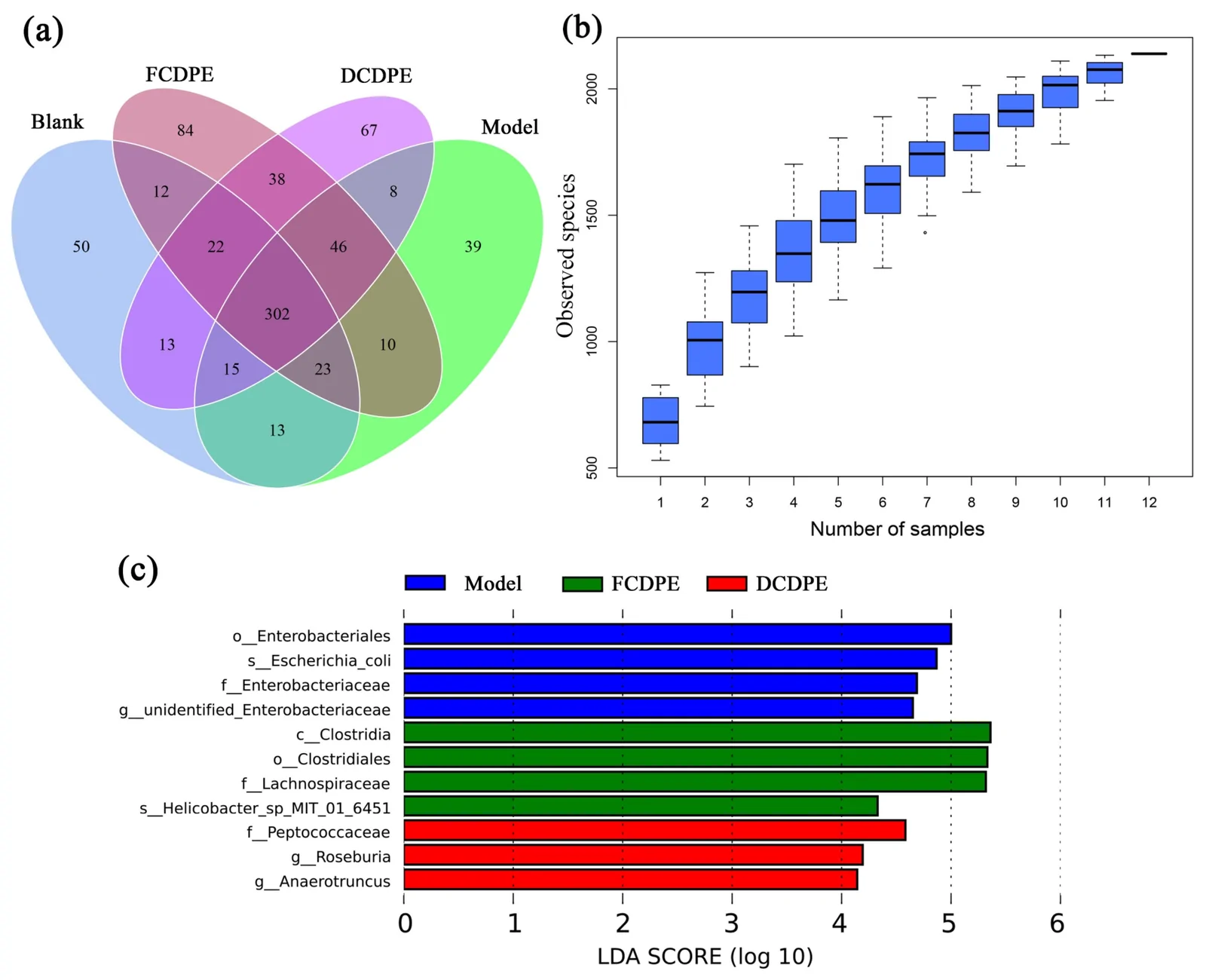
Venn diagram of shared and group-specific OTUs (**a**), sample-based observed-species accumulation curve (**b**), and LEfSe differential analysis (**c**) of the four groups (*n* = 3).

To assess the shared and group-specific bacterial communities, a four-set Venn diagram was constructed from the OTUs of the blank, model, FCDPE and DCDPE groups (Figure 7a) [34]. A core set of 302 OTUs was shared by all four groups, indicating that the basal community structure was largely consistent across groups. Beyond this common core, each group harboured unique OTUs (Blank, 50; Model, 39; FCDPE, 84; DCDPE, 67), with the largest number detected in the FCDPE group. This suggests that the extract interventions were accompanied by group-specific shifts in community composition; however, the number of unique OTUs alone should not be taken to be an index of community richness or health, and the OTU counts shown here are not directly comparable with the species counts of the accumulation curve below. The two panels summarise different properties of the data—presence/absence of OTUs across groups versus the accumulation of observed species with increasing sample number—so their totals are not expected to coincide [34].

A sample-based accumulation curve of observed species was plotted to describe how observed diversity accumulated with the number of samples (Figure 7b) [35]; this curve is a sample-accumulation plot and is not, by itself, evidence of per-sample sequencing-depth adequacy. As the number of samples increased from 1 to 12, the observed species rose steeply at first and then gradually levelled off, approaching a plateau at approximately 8–12 samples (about 2000–2100 observed species). The flattening of the curve indicates that additional sampling would reveal only a limited number of new species. Per-sample sequencing depth was assessed separately by Good’s coverage estimates, which confirmed that depth was sufficient for all samples.

To identify taxa that differed among the groups, LEfSe was performed and the results are shown in Figure 7c. At ranks above and below the family level, the order Enterobacterales and *Escherichia coli* were enriched in the model group, the class Clostridia and the order Clostridiales were enriched in the FCDPE group, and the genera *Roseburia* and *Anaerotruncus* were enriched in the DCDPE group. A *Helicobacter*-related annotation was also enriched in the FCDPE group (Figure 7c); because *Helicobacter* includes pathogenic members and the V3–V4 region affords limited resolution below the genus level, this annotation is reported without functional interpretation, and species-level annotations such as *Escherichia coli* should likewise be interpreted with caution. At the family level, Enterobacteriaceae, including the unclassified members annotated as unidentified_Enterobacteriaceae, was the most prominent biomarker of the model group. Members of this family frequently expand during gut microbial dysbiosis and accompany intestinal inflammation [36]; the enrichment observed in the immunosuppressed mice therefore points to a disturbed community structure in the model group. In the FCDPE group, Lachnospiraceae was the family-level biomarker, together with the class Clostridia and the order Clostridiales. Lachnospiraceae are obligate anaerobes, many of which produce substantial amounts of SCFAs, notably butyrate, and are generally regarded as commensals of a healthy gut [37]. The enrichment may thus reflect a community retaining SCFA-producing commensals. Peptococcaceae was the family-level marker of the DCDPE group, accompanied by the genera *Roseburia* and *Anaerotruncus*. Peptococcaceae is a family of strictly anaerobic, fermentative bacteria that occupy specialised niches in the gut [38], and the functional significance of this family in the present context therefore remains to be defined.

### 3.7. Differential Metabolites

**Figure 8:**
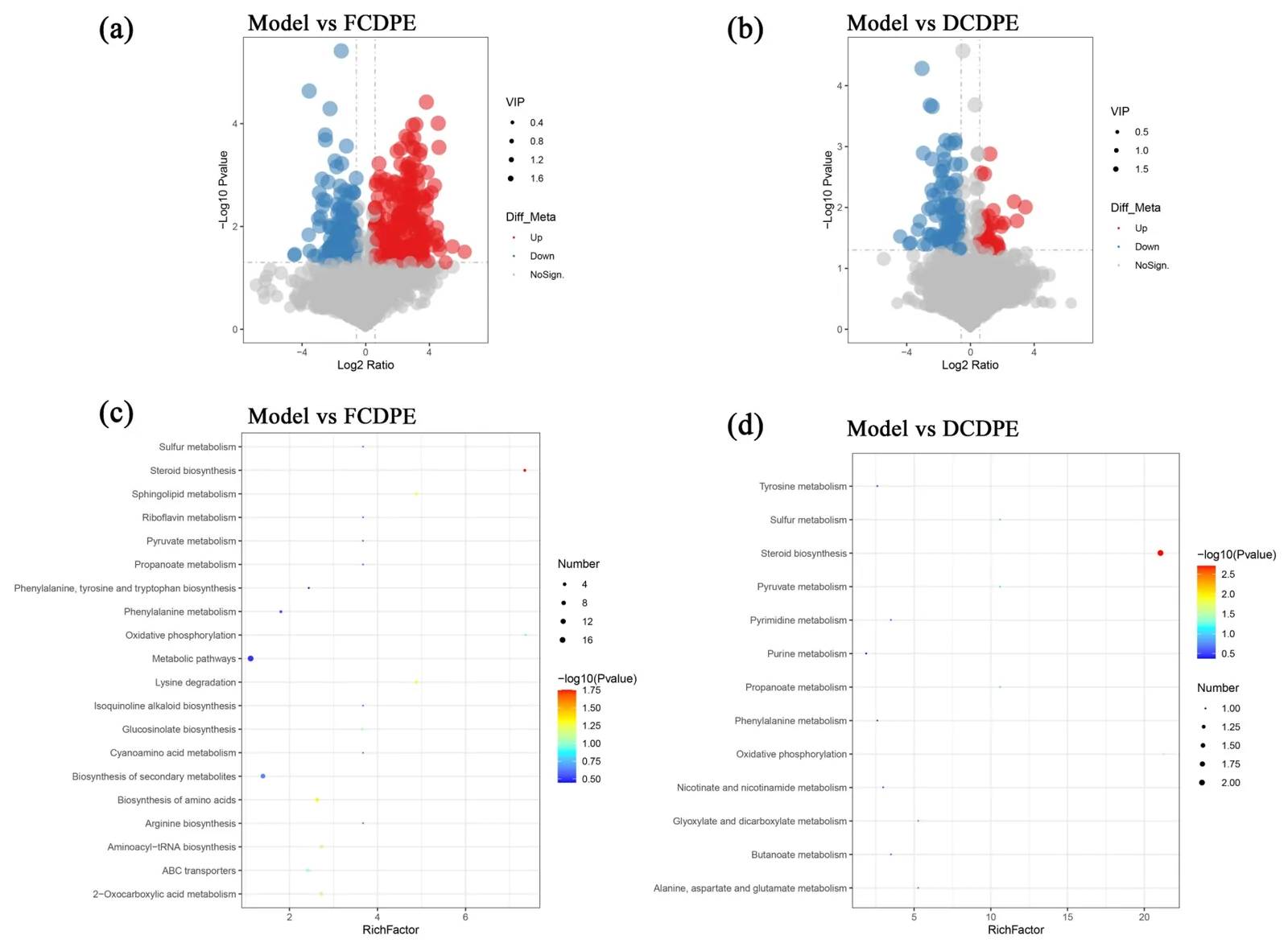
Volcano plots and KEGG pathway enrichment for FCDPE (**a**,**c**) and DCDPE (**b**,**d**). The *x*-axis gives the enrichment ratio of each pathway, point size the number of mapped differential metabolites and colour −log10(*p*) (*n* = 3).

Metabolites differing between the model group and each preparation were visualised as volcano plots (Figure 8a,b); these differential lists are exploratory, as they were defined at *p* < 0.05 and the annotations are database-based. A total of 434 differential metabolites (284 up-regulated, 150 down-regulated) were identified in the FCDPE group compared with the model group (Figure 8a; Table S1), and 138 (36 up-regulated, 102 down-regulated) in the DCDPE group (Figure 8b; Table S2). The two comparisons shared 82 differential metabolites, all changing in the same direction (70 commonly down-regulated, 12 commonly up-regulated), with none responding oppositely between the preparations. When grouped by chemical class, amino acids, oligopeptides and their derivatives formed a major group, in which most peptide features were elevated in the FCDPE group together with several aromatic amino acid derivatives, whereas only a few peptide features were affected in the DCDPE group. Amino acids and their derivatives, and aromatic amino acid metabolites in particular, act as substrates and signalling molecules that shape dendritic-cell, T-cell and B-cell responses [39]; the broader elevation of this class in the FCDPE group indicates a greater availability of substrates for lymphocyte activation relative to the model group, whereas the limited response in the DCDPE group suggests that this class was little affected by that preparation. Short-chain acylcarnitines, such as tiglylcarnitine, were also elevated in the FCDPE group. The carnitine/acylcarnitine pool reflects mitochondrial fatty-acid oxidation and energy homeostasis and participates in the regulation of inflammation and immune function [40]. This increase is consistent with a more favourable energy-metabolic state in the FCDPE group, with no comparable elevation in the DCDPE group. By contrast, the five lysophospholipid features shared by the two comparisons, including lysophosphatidylcholine (LysoPC) and lysophosphatidylethanolamine (LPE) species, were decreased in both groups, which is consistent with weakened bioactive lipid-mediated inflammatory signalling [41], as were ceramides (Cer) and related sphingolipids, a decrease consistent with lower sphingolipid-linked stress and inflammatory tone [42]. Succinic acid was among the organic acids commonly decreased; since succinate accumulates during inflammation and stabilises hypoxia-inducible factor-1α to amplify inflammatory responses, the decrease in both groups points to a dampened inflammatory metabolic state [43]. Among nucleosides and bases, hypoxanthine was decreased in both groups, whereas uridine was elevated in the DCDPE group; given that gut bacteria are major drivers of host purine homeostasis and that nucleotide availability is linked to immune cell proliferation, these shifts point to altered nucleotide salvage and turnover, which differed between the two preparations [44]. Sulfated bile acid and sterols were decreased in both preparations, suggesting modulation of the microbial–host bile acid and sterol axis, whose metabolites act as immune signalling molecules, as exemplified by the recognition of a sulfated bile acid by MAIT cells [45]. Therefore, both extract interventions were associated with a partial reversal of immunosuppression-associated metabolic changes in this model, in line with the immune recovery detected in the spleen, colon and circulating lymphocyte subsets.

### 3.8. Pathway Enrichment of Differential Metabolites

The differential metabolites were further mapped onto KEGG pathways (Figure 8c,d), and pathway enrichment was evaluated by a hypergeometric test against a background of the 192 metabolites that were detected in this study and annotated in KEGG, with Benjamini–Hochberg-adjusted *p* values; in Figure 8c,d the *x*-axis gives the enrichment ratio for each pathway, the point size encodes the number of differential metabolites mapped to that pathway and the colour encodes −log10(*p*). Steroid biosynthesis (map00100) was the most significantly enriched pathway in both comparisons, showing the highest −log10(*p*) among the 20 enriched pathways in the FCDPE group (Figure 8c; *p* = 0.018, adjusted *p* = 0.44) and among the 13 in the DCDPE group (Figure 8d; *p* = 0.0020, adjusted *p* = 0.029, the only pathway that remained significant after Benjamini–Hochberg correction). In both groups, the enrichment of steroid biosynthesis was driven mainly by a coordinated decrease in sterol-related metabolites relative to the model group, including cholest-4-en-3-one, stigmasterol and ergocalciferol, with 7-oxocholesterol additionally decreased in the DCDPE group. Sterols and their oxidised derivatives are established regulators of immune cell function: cholesterol is a key component of the membrane lipid rafts that support T- and B-cell receptor signalling, and oxysterols act as endogenous lipid mediators that modulate immune cell trafficking and intestinal inflammation, in part through receptors such as GPR183 [46]. In this context, the lower sterol-related metabolite levels are consistent with a reduced sterol-associated inflammatory lipid tone, in line with the decreases in lysophospholipids, ceramides and succinate described above. Notably, several steroid-hormone-related features changed in the opposite direction—estrone and β-estradiol were markedly higher in the FCDPE group, and corticosterone was higher in the DCDPE group—indicating that sterol-related metabolism was redistributed rather than uniformly suppressed. Because only 26 (FCDPE) and 9 (DCDPE) of the differential metabolites could be assigned to KEGG pathways, most of the displayed pathways rest on only one or two mapped metabolites—for example, map00100 is supported by stigmasterol and ergocalciferol—and the enrichment results should therefore be read as exploratory.

## 4. Discussion

The compositional differences between FCDPE and DCDPE point to the drying step rather than to the plant itself, since the two materials originated from the same harvested batch. Both extracts were predominantly carbohydrates (total carbohydrate 80.82% vs. 82.24%) with only minor amounts of protein, uronic acid and reducing sugars, so the differences discussed below are unlikely to reflect differences in overall purity or in the administered carbohydrate load. The two extracts showed comparable main-peak apparent molecular weights and FT-IR absorptions common to both. The fresh extract contained markedly more arabinose than the dried extract (20.24% vs. 6.54%), with a correspondingly lower glucose proportion (54.74% vs. 74.87%); the three minor high-molecular-weight fractions of DCDPE were systematically smaller than those of FCDPE, and the band near 764 cm^−1^ was displaced by approximately 14 cm^−1^. These differences are consistent with the loss, degradation or poorer extractability of arabinose-rich material after drying; because the main-peak molecular weight was essentially unchanged (1.55 vs. 1.62 × 10^4^ Da), the more parsimonious interpretation is that arabinose resides in separate arabinan/arabinogalactan-type populations rather than in side chains of a single backbone (Figure 1, Figure 2 and Figure 3). Drying conditions are known to alter the molecular weight, fine structure and biological potency of food polysaccharides [47], and controlled degradation of an arabinogalactan has been shown to change both its structural characteristics and its immunomodulatory activity [48]. The immunomodulatory potency of a polysaccharide is now understood to depend on fine structural features such as molecular weight, branching and monosaccharide composition [49]; definitive statements about branching, linearity and side-chain topology would require methylation/linkage analysis and NMR on purified fractions, which were not performed in this study.

Because both preparations acted on mice whose community had first been disturbed by cyclophosphamide, the signatures they left are best read as different routes back to a less dysbiotic state. The disturbance itself was the expected pattern: Muribaculaceae remained the most abundant family in all groups, whereas Enterobacteriaceae was the most strongly LEfSe-enriched family-level biomarker of the model group, with the order Enterobacterales and *Escherichia coli* contributing at other ranks—a pattern typical of the facultative-anaerobe expansion that follows chemotherapy-induced barrier damage; LEfSe enrichment is thus distinguished from numerical dominance. Within that context, the two preparations diverged. The fresh preparation was marked by Lachnospiraceae, together with the class Clostridia and the order Clostridiales; because members of this family are obligate anaerobes and established butyrate producers generally regarded as commensals of a healthy gut, and because Lachnospiraceae have been shown to contribute directly to the anti-colitis effects of a bacterial exopolysaccharide [50], this association is mechanistically attractive. Studies of processed herbal and microbial polysaccharides in cyclophosphamide-treated mice likewise report that immune recovery is accompanied by a restoration of saccharolytic taxa [51,52]. The dried preparation, by contrast, was marked by Peptococcaceae, together with the genera *Roseburia* and *Anaerotruncus*; the functional role of this family under the present conditions remains to be defined, and its enrichment cannot yet be assigned a direction.

The faecal metabolome provides the functional link between the community shifts described above and the recovery observed at the cellular level. The FCDPE group showed a broader expansion of oligopeptides, amino acids and their aromatic derivatives, together with short-chain acylcarnitines, whereas both preparations lowered ceramides and succinic acid, together with the five lysophospholipid features shared by the two comparisons; KEGG enrichment placed steroid biosynthesis at the top of both comparisons, driven by lower sterol-related metabolites (Figure 8). These directions are consistent with—though they do not prove the existence of—a less inflammatory and more substrate-replete metabolic environment: amino acids and their derivatives serve both as biosynthetic substrates and as signals that shape dendritic-cell, T-cell and B-cell responses, and colonic microbial amino-acid metabolism is increasingly recognised as a central regulator of gut health [53]. Microbiota- and metabolite-driven control of immune cell dynamics is well documented [54], and the decrease in succinic acid is of particular interest, because this tricarboxylic-acid-cycle intermediate accumulates during inflammation and stabilises hypoxia-inducible factor-1α, thereby amplifying inflammatory signalling; its reduction is therefore consistent with a dampened inflammatory metabolic state [55]. The lower sterol-related metabolite levels point in the same direction, as sterols and their oxidised derivatives modulate immune cell trafficking and intestinal inflammation. Importantly, these metabolic changes ran in parallel with the cytological recovery, in which circulating CD3^+^ and CD19^+^ proportions rose relative to the model group and the splenic and colonic architecture improved (Figure 4 and Figure 5); a recent multi-omics study of a cyclophosphamide model similarly reported that immune alleviation was accompanied by coordinated shifts in the gut microbiota and the faecal metabolome [56]. Taken together, the metabolic reorganisation we observed offers one plausible—but untested—link between the compositional and community signatures of the two extracts and their convergent, though incomplete, immune recovery. The present dataset nevertheless describes co-variation only and cannot establish the direction of causality among structure, community and host response.

Several limitations temper the conclusions above. First, the material studied is a pair of crude, ethanol-precipitated extracts rather than purified polysaccharides. The phenol–sulfuric acid assay also detects free sugars and oligosaccharides, so the 80–82% total-carbohydrate values should be read as an upper estimate of the polysaccharide content. Total phenolics, including phenylethanoid glycosides, and free sugars were not measured, and their absence from the extracts cannot be assumed: phenolic glycosides are partly co-precipitated by 75% (*v*/*v*) ethanol, and the partition of these constituents between precipitate and supernatant was not determined. Because phenolics and free sugars are themselves immunomodulatory and are fermented by the gut microbiota, they may contribute to the observed effects. Second, the structural evidence is of limited resolution. The pattern of glycosidic linkages governs how a polysaccharide is fermented by the gut microbiota [57]; linkage-resolved analysis by methylation-GC-MS together with 1D/2D NMR on homogeneous fractions will be required before the present structural description can be converted into a mechanistic structure–activity account. Third, the untargeted metabolomic and 16S rRNA analyses were exploratory in design. Annotation was performed against public databases without MS/MS confirmation, and differential metabolites were screened using an unadjusted threshold of *p* < 0.05, so some identified metabolites may represent false positives or exogenous contaminants; every feature in Tables S1 and S2 is labelled either “Confident compound” or “Suspected contaminant”, and features labelled as suspected contaminants were excluded from all pathway-level interpretation. The omics analyses were based on three animals per group, without pooled quality-control samples, internal standards or any assessment of analytical drift, and no formal power calculation was performed. In addition, the 82 metabolites shared by the two comparisons all changed in the same direction (70 down and 12 up, with none opposing), which may reflect a systematic technical effect that the present data cannot exclude. Accordingly, all metabolomic and microbiome inferences reported here are exploratory and hypothesis-generating. Fourth, the 16S rRNA V3–V4 amplicon sequencing provided limited taxonomic resolution and no direct functional information, and the relative abundance data were compositional [58]. Fifth, only correlative evidence was obtained in this study. Germ-free, antibiotic depletion or faecal microbiota transplantation assays were not conducted so the causal contribution of gut microbiota to the observed immune recovery remained unproven [59]. These validation approaches were regarded as the clearest direction for follow-up investigations. Sixth, only female mice and a single dosage were adopted, and no positive controls were included. Histological assessment was restricted to preliminary qualitative morphological observation without quantitative measurement.

## 5. Conclusions

In this study, differences in the preliminary structural characteristics of crude polysaccharide extracts from fresh (FCDPE) and dried (DCDPE) *C. deserticola* were compared and linked to immunological, microbiome and metabolic indicators in mice with immunosuppression induced by cyclophosphamide. Similar apparent molecular weights of the major peaks were observed for both extracts, whereas a markedly higher proportion of arabinose and greater apparent molecular weights of the minor high-molecular-weight fractions were detected in the fresh extract. Both extracts were associated with partial recovery from immunosuppression, as increased proportions of circulating CD3^+^ and CD19^+^ cells and improved structural statuses of the spleen and colon were demonstrated. Each extract was associated with a distinct signature in the perturbed gut microbiota, with Lachnospiraceae for FCDPE and Peptococcaceae for DCDPE, and these microbial differences were accompanied by metabolic changes consistent with a reduced inflammatory status; after Benjamini–Hochberg correction, however, only steroid biosynthesis in the DCDPE comparison remained significant (adjusted *p* = 0.029; adjusted *p* = 0.44 in the FCDPE comparison). Accordingly, the drying step during primary processing altered the composition of the extracts. The two preparations showed distinct microbial and metabolic profiles alongside convergent, though partial, recovery of the measured immune readouts. These microbial and metabolic findings are exploratory and hypothesis-generating and require confirmation in larger, independently validated datasets.

## Data Availability

All data supporting the findings of this study are available from the corresponding authors upon reasonable request.

## Supplementary Materials

The following supporting information can be downloaded at: https://www.mdpi.com/article/10.3390/foods15213955/s1. Table S1: Differential metabolites in the FCDPE group compared with the model group; Table S2: Differential metabolites in the DCDPE group compared with the model group.
