# Supplementary material for: Crude Polysaccharide Extracts from Fresh and Dried Cistanche deserticola Attenuate Cyclophosphamide-Induced Immunosuppression in Mice
Source: Foods. 2026 Nov 4;15(21):3955. doi: 10.3390/foods15213955 (PMC13762638; doi:10.3390/foods15213955)
Supplement: Supplementary file 1 [file foods-15-03955-s001.zip › foods-4658239-supplementary.pdf]

Supplementary Table S1. Differential metabolites in the FCDPE group compared with the model group.

ID	Description	Formula	RT (min)	m/z	Model1	Model2	Model3	FCDPE1	FCDPE2	FCDPE3	FC	log2FC	P value	ROC	VIP	Up/Down	Annotation category
C8	Choline	$C_5H_{13}NO$	0.52	103.10	5.31×10^{8}	5.05×10^{8}	3.63×10^{8}	2.27×10^{8}	3.56×10^{8}	2.43×10^{8}	0.59	-0.76	4.47×10^{-2}	1.00	1.35	down	Confident compound
C41	1,4-dihydroxyheptadec-16-en-2-yl acetate	$C_{19}H_{36}O_4$	6.93	328.26	1.62×10^{8}	1.64×10^{8}	1.74×10^{8}	1.05×10^{8}	9.74×10^{7}	6.02×10^{7}	0.53	-0.93	5.43×10^{-3}	1.00	1.54	down	Confident compound
C44	Palmitoyl ethanolamide	$C_{18}H_{37}NO_2$	8.29	299.28	2.07×10^{8}	2.80×10^{8}	1.39×10^{8}	7.69×10^{7}	5.22×10^{7}	4.25×10^{7}	0.27	-1.86	2.26×10^{-2}	1.00	1.45	down	Confident compound
C45	Emetine	$C_{29}H_{40}N_2O_4$	6.74	480.30	1.53×10^{8}	1.99×10^{8}	1.62×10^{8}	6.66×10^{7}	1.20×10^{8}	1.07×10^{8}	0.57	-0.81	2.61×10^{-2}	1.00	1.41	down	Suspected contaminant
C101	Stearoyl Ethanolamide	$C_{20}H_{41}NO_2$	9.07	327.31	1.45×10^{8}	1.65×10^{8}	6.88×10^{7}	3.40×10^{7}	3.87×10^{7}	3.99×10^{7}	0.30	-1.75	3.91×10^{-2}	1.00	1.38	down	Confident compound
C104	Dicyclomine	$C_{19}H_{35}NO_2$	6.87	309.27	8.85×10^{7}	1.47×10^{8}	1.24×10^{8}	1.50×10^{7}	1.52×10^{7}	2.44×10^{7}	0.15	-2.72	4.10×10^{-3}	1.00	1.55	down	Suspected contaminant
C107	Stigmasterol	$C_{29}H_{48}O$	12.97	412.37	1.23×10^{8}	1.33×10^{8}	1.08×10^{8}	8.18×10^{7}	5.29×10^{7}	4.20×10^{7}	0.48	-1.05	1.08×10^{-2}	1.00	1.51	down	Confident compound
C134	Hypoxanthine	$C_5H_4N_4O$	0.56	136.04	5.26×10^{8}	1.01×10^{9}	1.28×10^{9}	2.06×10^{8}	1.32×10^{8}	2.17×10^{8}	0.20	-2.34	2.75×10^{-2}	1.00	1.40	down	Confident compound
C148	Pinolenic acid	$C_{18}H_{30}O_2$	6.94	278.22	3.70×10^{7}	3.79×10^{7}	4.14×10^{7}	2.79×10^{7}	2.50×10^{7}	1.55×10^{7}	0.59	-0.76	1.61×10^{-2}	1.00	1.47	down	Confident compound
C164	D-Sphingosine	$C_{18}H_{37}NO_2$	6.96	299.28	9.12×10^{7}	5.55×10^{7}	5.15×10^{7}	2.14×10^{7}	1.26×10^{7}	8.49×10^{6}	0.21	-2.22	1.69×10^{-2}	1.00	1.48	down	Confident compound
C165	LysoPC(O-18:0/0:0)	$C_{26}H_{56}NO_6P$	8.30	509.39	8.04×10^{7}	7.12×10^{7}	6.37×10^{7}	4.41×10^{7}	2.12×10^{7}	1.61×10^{7}	0.38	-1.40	1.06×10^{-2}	1.00	1.52	down	Confident compound
C168	Aliskiren	$C_{30}H_{53}N_3O_6$	8.92	551.40	3.70×10^{7}	4.54×10^{7}	3.29×10^{7}	1.84×10^{7}	1.43×10^{7}	1.37×10^{7}	0.40	-1.31	4.47×10^{-3}	1.00	1.56	down	Suspected contaminant
C183	Pyrogallol	$C_6H_6O_3$	2.14	126.03	2.66×10^{7}	7.60×10^{7}	7.87×10^{7}	9.99×10^{6}	9.31×10^{6}	1.16×10^{7}	0.17	-2.55	4.15×10^{-2}	1.00	1.34	down	Confident compound
C197	1,1-Bi(cyclohexyl)	$C_{12}H_{22}$	8.10	166.17	5.80×10^{7}	7.60×10^{7}	5.60×10^{7}	6.49×10^{6}	1.19×10^{7}	1.00×10^{7}	0.15	-2.74	1.20×10^{-3}	1.00	1.60	down	Suspected contaminant
C200	Tiamulin	$C_{28}H_{47}NO_4S$	6.70	493.32	5.94×10^{6}	8.90×10^{6}	7.93×10^{6}	2.34×10^{6}	2.34×10^{6}	1.64×10^{6}	0.28	-1.85	3.71×10^{-3}	1.00	1.55	down	Suspected contaminant
C233	Ethyl 4-acetyl-3,5-dimethyl-1H-pyrrole-2-carboxylate	$C_{11}H_{15}NO_3$	3.81	209.11	4.04×10^{6}	4.95×10^{6}	5.75×10^{6}	3.66×10^{6}	2.03×10^{6}	1.95×10^{6}	0.52	-0.95	3.32×10^{-2}	1.00	1.39	down	Suspected contaminant
C253	NAGly 15:0/16:0	$C_{33}H_{63}NO_5$	12.88	553.47	4.79×10^{7}	3.70×10^{7}	4.75×10^{7}	1.49×10^{7}	3.45×10^{7}	2.06×10^{7}	0.53	-0.92	3.78×10^{-2}	1.00	1.36	down	Confident compound
C290	7-(Diethylamino)-4-methyl-2H-chromen-2-one	$C_{14}H_{17}NO_2$	6.63	231.13	3.51×10^{7}	3.92×10^{7}	3.47×10^{7}	1.65×10^{7}	1.16×10^{7}	1.01×10^{7}	0.35	-1.51	5.99×10^{-4}	1.00	1.61	down	Suspected contaminant
C295	Stearoylcarnitine	$C_{25}H_{49}NO_4$	8.49	427.37	1.56×10^{7}	2.87×10^{7}	2.53×10^{7}	1.01×10^{7}	1.27×10^{7}	1.14×10^{7}	0.49	-1.02	4.28×10^{-2}	1.00	1.34	down	Confident compound
C312	BSALBDFYJLSIOZ-HOUCIZQNSA-N	$C_{29}H_{48}O_3$	12.07	444.36	2.32×10^{7}	4.21×10^{7}	3.29×10^{7}	1.34×10^{7}	1.44×10^{7}	1.27×10^{7}	0.41	-1.28	2.48×10^{-2}	1.00	1.42	down	Confident compound
C313	PI-Cer 35:0;3O	$C_{41}H_{82}NO_{12}P$	13.08	811.56	3.70×10^{7}	2.45×10^{7}	2.43×10^{7}	9.43×10^{6}	9.19×10^{6}	9.87×10^{6}	0.33	-1.59	1.04×10^{-2}	1.00	1.52	down	Confident compound
C376	N-(2-Hydroxyethyl)-9-octadecenamide	$C_{20}H_{39}NO_2$	8.80	325.30	3.19×10^{7}	2.63×10^{7}	1.03×10^{7}	2.73×10^{6}	5.57×10^{6}	5.71×10^{6}	0.20	-2.29	4.98×10^{-2}	1.00	1.35	down	Confident compound
C412	Methyl isoferulate	$C_{11}H_{12}O_4$	4.94	208.07	1.18×10^{7}	1.27×10^{7}	1.69×10^{7}	9.81×10^{6}	5.96×10^{6}	4.99×10^{6}	0.50	-1.00	3.26×10^{-2}	1.00	1.39	down	Confident compound
C422	4-hydroxy-6-[2-(2-methyl-1,2,4a,5,6,7,8,8a-octahydronaphthalen-1-yl)ethyl]oxan-2-one	$C_{18}H_{28}O_3$	9.33	292.20	2.38×10^{7}	2.17×10^{7}	2.50×10^{7}	1.48×10^{7}	1.02×10^{7}	8.00×10^{6}	0.47	-1.10	4.79×10^{-3}	1.00	1.55	down	Confident compound
C443	Cer 18:2;2O/2:0	$C_{20}H_{37}NO_3$	7.03	339.28	1.13×10^{7}	1.12×10^{7}	1.09×10^{7}	8.23×10^{6}	6.85×10^{6}	7.01×10^{6}	0.66	-0.60	1.15×10^{-3}	1.00	1.61	down	Confident compound

ID	Description	Formula	RT (min)	m/z	Model1	Model2	Model3	FCDPE1	FCDPE2	FCDPE3	FC	log2FC	P value	ROC	VIP	Up/Down	Annotation category
C472	QOLRLLFJMZLYQJ-KVERTZGMSA-N	$C_{27}H_{42}O_4$	7.18	430.30	2.01×10^{7}	1.53×10^{7}	1.94×10^{7}	6.37×10^{6}	6.78×10^{6}	4.82×10^{6}	0.33	-1.61	1.63×10^{-3}	1.00	1.59	down	Confident compound
C481	Cer 17:1;2O/16:0	$C_{33}H_{65}NO_3$	12.80	523.50	2.21×10^{7}	1.83×10^{7}	1.10×10^{7}	4.56×10^{6}	5.74×10^{6}	5.06×10^{6}	0.30	-1.74	2.17×10^{-2}	1.00	1.46	down	Confident compound
C501	N,N-Dimethylsphingosine	$C_{20}H_{41}NO_2$	7.75	327.31	1.58×10^{7}	9.61×10^{6}	7.89×10^{6}	5.23×10^{6}	3.31×10^{6}	3.10×10^{6}	0.35	-1.52	4.40×10^{-2}	1.00	1.38	down	Confident compound
C511	Ethoxyquin	$C_{14}H_{19}NO$	6.03	217.15	7.41×10^{6}	6.22×10^{6}	5.47×10^{6}	3.92×10^{6}	1.59×10^{6}	1.50×10^{6}	0.37	-1.45	1.44×10^{-2}	1.00	1.50	down	Suspected contaminant
C521	PI-Cer 33:0;3O	$C_{39}H_{78}NO_{12}P$	13.09	783.53	2.15×10^{7}	1.21×10^{7}	1.08×10^{7}	3.84×10^{6}	2.60×10^{6}	2.74×10^{6}	0.21	-2.28	2.53×10^{-2}	1.00	1.44	down	Confident compound
C532	Irganox 1010 degradation product C19H30O3	$C_{19}H_{30}O_3$	9.84	306.22	2.05×10^{7}	1.28×10^{7}	1.05×10^{7}	5.84×10^{6}	6.55×10^{6}	4.13×10^{6}	0.38	-1.41	4.21×10^{-2}	1.00	1.38	down	Suspected contaminant
C538	LysoPI(18:0/0:0)	$C_{27}H_{53}O_{12}P$	7.41	600.33	1.36×10^{7}	9.37×10^{6}	1.32×10^{7}	4.41×10^{6}	4.65×10^{6}	4.26×10^{6}	0.37	-1.44	4.98×10^{-3}	1.00	1.55	down	Confident compound
C547	Arg-Ile-Lys	$C_{18}H_{37}N_7O_4$	5.54	415.29	1.22×10^{7}	1.19×10^{7}	1.04×10^{7}	6.97×10^{6}	8.40×10^{6}	7.49×10^{6}	0.66	-0.60	4.75×10^{-3}	1.00	1.55	down	Confident compound
C564	NP-008952	$C_{12}H_{20}O_4$	4.94	228.14	1.51×10^{7}	1.02×10^{7}	1.47×10^{7}	4.52×10^{6}	4.18×10^{6}	3.68×10^{6}	0.31	-1.69	4.54×10^{-3}	1.00	1.56	down	Confident compound
C580	Cer 20:0;2O/2:0	$C_{22}H_{45}NO_3$	8.34	371.34	9.75×10^{6}	9.12×10^{6}	9.21×10^{6}	3.26×10^{6}	4.57×10^{6}	4.28×10^{6}	0.43	-1.21	2.72×10^{-4}	1.00	1.62	down	Confident compound
C648	LysoPC(18:2(9Z,12Z)/0:0)	$C_{26}H_{50}NO_7P$	7.89	519.33	1.02×10^{7}	1.63×10^{7}	5.61×10^{6}	3.21×10^{5}	1.75×10^{6}	1.28×10^{6}	0.10	-3.26	3.73×10^{-2}	1.00	1.38	down	Confident compound
C649	Cholest-4-en-3-one	$C_{27}H_{44}O$	11.88	384.34	1.56×10^{7}	1.31×10^{7}	9.14×10^{6}	4.92×10^{6}	4.01×10^{6}	3.72×10^{6}	0.33	-1.58	1.19×10^{-2}	1.00	1.51	down	Confident compound
C651	Phytosphingosine	$C_{18}H_{39}NO_3$	6.21	317.29	4.66×10^{6}	3.71×10^{6}	4.72×10^{6}	1.35×10^{6}	3.04×10^{6}	3.07×10^{6}	0.57	-0.81	4.54×10^{-2}	1.00	1.34	down	Confident compound
C755	LysoPC(18:1(9Z)/0:0)	$C_{26}H_{52}NO_7P$	8.61	521.35	8.40×10^{6}	1.31×10^{7}	8.24×10^{6}	5.50×10^{5}	1.85×10^{6}	1.76×10^{6}	0.14	-2.84	6.46×10^{-3}	1.00	1.53	down	Confident compound
C769	Hexadecyl ferulate	$C_{26}H_{42}O_4$	8.14	418.31	6.49×10^{6}	5.37×10^{6}	3.38×10^{6}	2.37×10^{6}	2.49×10^{6}	2.13×10^{6}	0.46	-1.12	3.98×10^{-2}	1.00	1.39	down	Confident compound
C816	Riboflavin	$C_{17}H_{20}N_4O_6$	3.38	376.14	3.77×10^{6}	3.72×10^{6}	3.85×10^{6}	2.13×10^{6}	3.19×10^{6}	1.91×10^{6}	0.64	-0.65	2.60×10^{-2}	1.00	1.40	down	Confident compound
C841	Bis(4-methylpentyl) phthalate	$C_{20}H_{30}O_4$	4.51	334.21	8.62×10^{6}	7.07×10^{6}	8.92×10^{6}	6.50×10^{6}	4.72×10^{6}	3.64×10^{6}	0.60	-0.73	3.25×10^{-2}	1.00	1.40	down	Suspected contaminant
C856	Alpha-Linolenic acid	$C_{18}H_{30}O_2$	9.02	278.22	4.08×10^{6}	3.75×10^{6}	3.51×10^{6}	2.83×10^{6}	2.55×10^{6}	1.90×10^{6}	0.64	-0.64	1.33×10^{-2}	1.00	1.50	down	Confident compound
C867	Cer 18:0;2O/18:2	$C_{36}H_{69}NO_3$	12.73	563.53	4.76×10^{6}	7.19×10^{6}	4.54×10^{6}	2.37×10^{6}	1.26×10^{6}	1.43×10^{6}	0.31	-1.70	1.43×10^{-2}	1.00	1.49	down	Confident compound
C902	Pentadecanoyl Ethanolamide	$C_{17}H_{35}NO_2$	7.86	285.27	5.08×10^{6}	4.40×10^{6}	2.75×10^{6}	6.66×10^{5}	2.89×10^{5}	5.98×10^{5}	0.13	-2.98	7.06×10^{-3}	1.00	1.54	down	Confident compound
C968	Diisodecyl phthalate	$C_{28}H_{46}O_4$	8.98	446.34	1.76×10^{7}	7.02×10^{6}	9.84×10^{6}	6.23×10^{5}	1.28×10^{6}	1.09×10^{6}	0.09	-3.52	2.99×10^{-2}	1.00	1.42	down	Suspected contaminant
C972	2-Arachidonoyl glycerol	$C_{23}H_{38}O_4$	9.26	378.28	6.98×10^{6}	7.93×10^{6}	8.02×10^{6}	5.71×10^{6}	3.45×10^{6}	2.37×10^{6}	0.50	-0.99	2.16×10^{-2}	1.00	1.45	down	Confident compound
C1013	Mesitaldehyde	$C_{10}H_{12}O$	3.78	148.09	6.55×10^{6}	5.18×10^{6}	5.01×10^{6}	3.75×10^{6}	5.95×10^{5}	2.01×10^{6}	0.38	-1.40	2.86×10^{-2}	1.00	1.44	down	Confident compound
C1017	Cer 17:0;2O/2:0	$C_{19}H_{39}NO_3$	7.17	329.29	6.50×10^{6}	5.85×10^{6}	2.04×10^{6}	4.69×10^{5}	7.30×10^{5}	1.15×10^{6}	0.16	-2.62	4.60×10^{-2}	1.00	1.36	down	Confident compound
C1024	PE(14:0/24:1(15Z))	$C_{43}H_{84}NO_8P$	9.23	773.59	6.97×10^{5}	8.52×10^{5}	6.28×10^{5}	4.58×10^{5}	4.18×10^{5}	4.37×10^{5}	0.60	-0.73	1.28×10^{-2}	1.00	1.50	down	Confident compound
C1043	NP-015559	$C_{17}H_{14}O_7$	4.93	330.07	2.03×10^{6}	1.66×10^{6}	1.66×10^{6}	9.21×10^{5}	1.05×10^{6}	6.08×10^{5}	0.48	-1.05	6.85×10^{-3}	1.00	1.53	down	Confident compound

ID	Description	Formula	RT (min)	m/z	Model1	Model2	Model3	FCDPE1	FCDPE2	FCDPE3	FC	log2FC	P value	ROC	VIP	Up/Down	Annotation category
C1061	MG(0:0/14:1(9Z)/0:0)	$C_{17}H_{32}O_4$	6.25	300.23	5.09×10^{6}	6.18×10^{6}	6.81×10^{6}	2.89×10^{6}	1.86×10^{6}	1.32×10^{6}	0.34	-1.57	4.22×10^{-3}	1.00	1.55	down	Confident compound
C1079	Phenylalanylthreonine	$C_{13}H_{18}N_2O_4$	3.55	266.13	2.03×10^{6}	3.32×10^{6}	3.80×10^{6}	1.34×10^{6}	3.24×10^{5}	5.91×10^{5}	0.25	-2.02	1.96×10^{-2}	1.00	1.45	down	Confident compound
C1086	Pro-Arg-Ala	$C_{14}H_{26}N_6O_4$	3.90	342.20	4.52×10^{6}	3.70×10^{6}	5.03×10^{6}	2.27×10^{6}	2.97×10^{5}	2.88×10^{5}	0.22	-2.21	1.05×10^{-2}	1.00	1.51	down	Confident compound
C1110	N,N-Diphenylguanidine	$C_{13}H_{13}N_3$	3.74	211.11	3.50×10^{5}	3.70×10^{5}	2.82×10^{5}	1.28×10^{5}	1.48×10^{5}	1.88×10^{5}	0.46	-1.11	4.90×10^{-3}	1.00	1.56	down	Suspected contaminant
C1127	2,2-Methylenebis[4-methyl-6-(1-methylcyclohexyl)phenol]	$C_{29}H_{40}O_2$	8.08	420.30	4.02×10^{6}	4.09×10^{6}	3.02×10^{6}	1.94×10^{6}	1.73×10^{6}	1.24×10^{6}	0.44	-1.18	6.85×10^{-3}	1.00	1.54	down	Suspected contaminant
C1145	AUDA	$C_{23}H_{40}N_2O_3$	10.96	392.31	6.00×10^{6}	4.71×10^{6}	4.42×10^{6}	3.05×10^{6}	2.98×10^{6}	2.46×10^{6}	0.56	-0.83	1.33×10^{-2}	1.00	1.50	down	Suspected contaminant
C1149	2,5-Bis(tert-butylperoxy)-2,5-dimethylhexane	$C_{16}H_{34}O_4$	7.26	290.25	4.20×10^{6}	3.11×10^{6}	3.85×10^{6}	2.09×10^{6}	2.78×10^{6}	2.53×10^{6}	0.66	-0.59	2.99×10^{-2}	1.00	1.40	down	Suspected contaminant
C1155	3-(3,5-di-tert-butyl-4-hydroxyphenyl)-N-[3-(3,5-di-tert-butyl-4-hydroxyphenyl)propanoyl]propanehydrazide	$C_{34}H_{52}N_2O_4$	8.09	552.39	5.81×10^{6}	5.34×10^{6}	3.04×10^{6}	1.89×10^{6}	1.99×10^{6}	1.82×10^{6}	0.40	-1.31	3.00×10^{-2}	1.00	1.42	down	Suspected contaminant
C1172	Pyrraline	$C_{12}H_{18}N_2O_4$	2.99	254.13	1.81×10^{6}	1.27×10^{6}	1.34×10^{6}	5.92×10^{5}	9.04×10^{5}	7.47×10^{5}	0.51	-0.98	1.88×10^{-2}	1.00	1.46	down	Confident compound
C1200	4,8-Dihydroxy-7-(hydroxymethyl)-6-methoxy-3,4-dihydro-1(2H)-naphthalenone	$C_{12}H_{14}O_5$	4.65	238.08	7.22×10^{5}	7.66×10^{5}	9.07×10^{5}	2.72×10^{5}	9.52×10^{4}	9.51×10^{4}	0.19	-2.37	1.37×10^{-3}	1.00	1.59	down	Confident compound
C1203	Tyr-Ala	$C_{12}H_{16}N_2O_4$	3.22	252.11	3.95×10^{6}	5.57×10^{6}	2.82×10^{6}	1.17×10^{6}	1.23×10^{6}	7.27×10^{5}	0.25	-1.98	1.95×10^{-2}	1.00	1.46	down	Confident compound
C1213	2-{5-[2-({2-[5-(2-Hydroxypropyl)tetrahydro-2-furanyl]propanoyl}oxy)propyl]tetrahydro-2-furanyl}propanoic acid	$C_{20}H_{34}O_7$	4.33	386.23	1.63×10^{6}	1.46×10^{6}	1.43×10^{6}	3.52×10^{5}	3.29×10^{5}	2.74×10^{5}	0.21	-2.24	5.11×10^{-5}	1.00	1.64	down	Confident compound
C1221	4-Acetylphenetole	$C_{10}H_{12}O_2$	8.93	164.08	3.52×10^{6}	3.16×10^{6}	4.28×10^{6}	1.26×10^{6}	1.39×10^{6}	9.98×10^{5}	0.33	-1.58	2.31×10^{-3}	1.00	1.57	down	Confident compound
C1235	4-fluoro-3-methyl-alpha-Pyrrolidinovalerophenone	$C_{16}H_{22}FNO$	3.04	263.17	4.51×10^{6}	5.27×10^{6}	3.02×10^{6}	9.21×10^{5}	4.03×10^{5}	1.40×10^{6}	0.21	-2.23	9.57×10^{-3}	1.00	1.52	down	Suspected contaminant
C1252	NP-015980	$C_{14}H_{24}O_6$	4.08	288.16	7.30×10^{5}	1.57×10^{6}	1.75×10^{6}	4.09×10^{5}	2.64×10^{5}	1.44×10^{5}	0.20	-2.31	2.92×10^{-2}	1.00	1.39	down	Confident compound
C1262	HexCer 18:1;2O/14:0	$C_{38}H_{73}NO_8$	9.57	671.53	2.68×10^{6}	3.74×10^{6}	3.11×10^{6}	2.12×10^{6}	1.80×10^{6}	1.14×10^{6}	0.53	-0.92	2.41×10^{-2}	1.00	1.43	down	Confident compound
C1281	diethyl 3-amino-6-methylthieno[2,3-b]pyridine-2,5-dicarboxylate	$C_{14}H_{16}N_2O_4S$	4.63	308.08	4.61×10^{6}	3.76×10^{6}	3.37×10^{6}	2.56×10^{6}	2.34×10^{6}	2.03×10^{6}	0.59	-0.76	1.59×10^{-2}	1.00	1.49	down	Suspected contaminant
C1293	NP-017176	$C_{27}H_{40}O_5$	9.32	444.28	2.07×10^{6}	1.69×10^{6}	1.57×10^{6}	9.33×10^{5}	6.08×10^{5}	3.77×10^{5}	0.36	-1.47	6.64×10^{-3}	1.00	1.55	down	Confident compound
C1305	Mebeverine	$C_{25}H_{35}NO_5$	6.82	429.25	1.49×10^{6}	1.81×10^{6}	1.44×10^{6}	1.06×10^{6}	7.61×10^{5}	4.44×10^{5}	0.48	-1.06	1.82×10^{-2}	1.00	1.46	down	Suspected contaminant
C1316	XZDFYPAYHVIUMX-UHFFFAOYSA-N	$C_{22}H_{43}NO_6$	4.50	417.31	2.37×10^{6}	2.94×10^{6}	1.74×10^{6}	1.44×10^{6}	1.25×10^{6}	5.81×10^{5}	0.46	-1.11	4.41×10^{-2}	1.00	1.36	down	Confident compound
C1335	MG(0:0/20:3(5Z,8Z,11Z)/0:0)	$C_{23}H_{40}O_4$	8.17	380.29	2.54×10^{6}	4.41×10^{6}	2.66×10^{6}	1.35×10^{6}	1.48×10^{6}	1.25×10^{6}	0.42	-1.24	3.89×10^{-2}	1.00	1.37	down	Confident compound
C1356	Methyl N-{[(2S)-1-(cyclohexylcarbamoyl)-4-(4-piperidinylcarbonyl)-2-piperazinyl]carbonyl}-D-alaninate	$C_{22}H_{37}N_5O_5$	7.40	451.28	2.86×10^{6}	3.10×10^{6}	2.99×10^{6}	1.30×10^{6}	1.90×10^{6}	1.67×10^{6}	0.54	-0.88	2.04×10^{-3}	1.00	1.57	down	Suspected contaminant
C1375	2,2-ETHYLIDENEBIS(4,6-DI-TERT-BUTYLPHENOL)	$C_{30}H_{46}O_2$	4.01	438.35	1.28×10^{6}	1.82×10^{6}	1.13×10^{6}	5.45×10^{5}	4.00×10^{5}	4.79×10^{5}	0.34	-1.57	1.22×10^{-2}	1.00	1.50	down	Suspected contaminant
C1382	Ethylbenzoate	$C_9H_{10}O_2$	9.23	150.07	2.77×10^{6}	1.20×10^{6}	2.29×10^{6}	1.82×10^{5}	1.67×10^{5}	1.74×10^{5}	0.08	-3.58	1.45×10^{-2}	1.00	1.49	down	Confident compound

ID	Description	Formula	RT (min)	m/z	Model1	Model2	Model3	FCDPE1	FCDPE2	FCDPE3	FC	log2FC	P value	ROC	VIP	Up/Down	Annotation category
C1384	11-(2,5-Dioxo-2,5-dihydro-1H-pyrrol-1-yl)undecanoic acid	$C_{15}H_{23}NO_4$	4.23	281.16	3.84×10^{6}	2.60×10^{6}	3.14×10^{6}	2.18×10^{6}	1.94×10^{6}	1.36×10^{6}	0.57	-0.81	3.38×10^{-2}	1.00	1.40	down	Suspected contaminant
C1404	Tris(2-butoxyethyl) phosphate	$C_{18}H_{39}O_7P$	7.57	398.24	1.74×10^{6}	2.29×10^{6}	1.54×10^{6}	2.74×10^{5}	4.98×10^{5}	2.02×10^{5}	0.17	-2.52	3.12×10^{-3}	1.00	1.57	down	Suspected contaminant
C1423	NP-021844	$C_{12}H_{24}O_5$	3.68	248.16	1.22×10^{6}	2.21×10^{6}	3.50×10^{6}	3.95×10^{5}	2.32×10^{5}	1.79×10^{5}	0.12	-3.10	3.69×10^{-2}	1.00	1.36	down	Confident compound
C1439	N-Formylphenylalanine	$C_{10}H_{11}NO_3$	3.67	193.07	5.34×10^{5}	9.64×10^{5}	9.87×10^{5}	4.31×10^{5}	2.15×10^{5}	2.30×10^{5}	0.35	-1.50	3.02×10^{-2}	1.00	1.40	down	Confident compound
C1468	Isoleucyl-Tyrosine	$C_{15}H_{22}N_2O_4$	2.83	294.16	1.97×10^{6}	1.74×10^{6}	1.27×10^{6}	9.84×10^{5}	1.04×10^{6}	3.96×10^{5}	0.49	-1.04	4.38×10^{-2}	1.00	1.36	down	Confident compound
C1478	Ergocalciferol	$C_{28}H_{44}O$	11.86	396.34	3.05×10^{6}	3.22×10^{6}	2.77×10^{6}	1.81×10^{6}	1.16×10^{6}	1.30×10^{6}	0.47	-1.08	2.65×10^{-3}	1.00	1.59	down	Confident compound
C1487	2,2-Methylenebis(6-cyclohexyl-4-methylphenol)	$C_{27}H_{36}O_2$	7.91	392.27	1.36×10^{6}	1.12×10^{6}	1.27×10^{6}	1.05×10^{6}	5.47×10^{5}	7.70×10^{5}	0.63	-0.67	4.58×10^{-2}	1.00	1.37	down	Suspected contaminant
C1504	6-Amino-5,6,7,8-tetrahydronaphthalene-2,3-diyl diacetate	$C_{14}H_{17}NO_4$	3.87	263.12	3.19×10^{5}	6.26×10^{5}	4.03×10^{5}	1.24×10^{5}	1.88×10^{5}	1.82×10^{5}	0.37	-1.45	3.86×10^{-2}	1.00	1.36	down	Suspected contaminant
C1560	KM 233	$C_{25}H_{30}O_2$	4.33	362.23	2.15×10^{6}	1.45×10^{6}	2.14×10^{6}	1.14×10^{6}	9.43×10^{5}	6.80×10^{5}	0.48	-1.06	2.04×10^{-2}	1.00	1.45	down	Suspected contaminant
C1566	Allyl 3-phenylpropanoate	$C_{12}H_{14}O_2$	4.38	190.10	1.62×10^{6}	2.48×10^{6}	2.55×10^{6}	1.50×10^{6}	9.78×10^{5}	8.72×10^{5}	0.50	-0.99	3.73×10^{-2}	1.00	1.37	down	Confident compound
C1576	Cer 18:1;2O/20:3	$C_{38}H_{69}NO_3$	12.31	587.53	1.60×10^{6}	1.27×10^{6}	8.23×10^{5}	2.51×10^{5}	1.21×10^{5}	1.22×10^{5}	0.13	-2.90	9.84×10^{-3}	1.00	1.53	down	Confident compound
C1613	3,9-Bis[(11-methyldodecyl)oxy]-2,4,8,10-tetraoxa-3,9-diphosphaspiro[5.5]undecane	$C_{31}H_{62}O_6P_2$	8.20	592.40	2.35×10^{6}	1.51×10^{6}	2.03×10^{6}	4.53×10^{5}	2.78×10^{5}	1.75×10^{5}	0.15	-2.70	2.96×10^{-3}	1.00	1.58	down	Suspected contaminant
C1618	N-(2-Furylmethyl)-3-{[5-(2-methyl-2-propanyl)-1,2-oxazol-3-yl]methyl}-3-oxetanamine	$C_{16}H_{22}N_2O_3$	3.90	290.16	1.53×10^{6}	1.41×10^{6}	2.31×10^{6}	1.17×10^{6}	5.67×10^{5}	4.91×10^{5}	0.42	-1.24	4.62×10^{-2}	1.00	1.34	down	Suspected contaminant
C1627	2-(1H-indol-3-yl)acetic acid	$C_{10}H_9NO_2$	2.89	175.06	8.12×10^{5}	1.53×10^{6}	1.04×10^{6}	4.92×10^{5}	3.92×10^{5}	3.68×10^{5}	0.37	-1.43	2.97×10^{-2}	1.00	1.40	down	Confident compound
C1651	Ethyl-2-hydroxyethyl nonanedioic acid	$C_{13}H_{24}O_6$	3.86	276.16	2.16×10^{6}	2.02×10^{6}	2.41×10^{6}	1.35×10^{6}	4.47×10^{5}	6.65×10^{5}	0.37	-1.42	9.44×10^{-3}	1.00	1.52	down	Confident compound
C1652	Diisononyl cyclohexane-1,2-dicarboxylate	$C_{26}H_{48}O_4$	9.25	424.36	8.78×10^{5}	1.07×10^{6}	7.92×10^{5}	5.77×10^{5}	5.27×10^{5}	5.51×10^{5}	0.60	-0.73	1.28×10^{-2}	1.00	1.50	down	Suspected contaminant
C1656	2,2-Iminodibenzoic acid	$C_{14}H_{11}NO_4$	4.95	257.07	6.31×10^{5}	7.95×10^{5}	8.83×10^{5}	4.77×10^{5}	3.84×10^{5}	4.29×10^{5}	0.56	-0.84	1.24×10^{-2}	1.00	1.48	down	Confident compound
C1686	Lys-Gly-Arg	$C_{14}H_{29}N_7O_4$	3.56	359.23	1.28×10^{6}	1.10×10^{6}	1.92×10^{6}	2.89×10^{5}	3.10×10^{5}	2.83×10^{5}	0.21	-2.28	1.04×10^{-2}	1.00	1.49	down	Confident compound
C1716	Sulfolithocholic acid	$C_{24}H_{40}O_6S$	10.66	456.25	2.83×10^{7}	2.50×10^{7}	1.42×10^{7}	8.78×10^{6}	3.73×10^{6}	4.97×10^{6}	0.26	-1.95	2.09×10^{-2}	1.00	1.47	down	Confident compound
C1735	5-hydroxy-4-[3-(2-hydroxypropan-2-yl)oxiran-2-yl]-1-methyl-7-oxabicyclo[4.1.0]hept-3-en-2-one	$C_{12}H_{16}O_5$	3.65	240.10	6.40×10^{5}	1.05×10^{6}	1.71×10^{6}	3.34×10^{5}	1.95×10^{5}	1.76×10^{5}	0.21	-2.27	4.69×10^{-2}	1.00	1.33	down	Confident compound
C1747	5-[5-(methoxycarbonyl)-5,8a-dimethyl-2-methylidene-decahydronaphthalen-1-yl]-3-methylpentanoic acid	$C_{21}H_{34}O_4$	5.41	350.25	4.67×10^{5}	4.54×10^{5}	4.71×10^{5}	1.73×10^{5}	1.57×10^{5}	1.50×10^{5}	0.34	-1.54	3.84×10^{-6}	1.00	1.64	down	Confident compound
C1767	NLK	$C_{16}H_{31}N_5O_5$	3.43	373.23	1.57×10^{6}	1.07×10^{6}	1.05×10^{6}	8.02×10^{5}	3.63×10^{5}	5.77×10^{5}	0.47	-1.08	3.66×10^{-2}	1.00	1.41	down	Confident compound
C1833	PE(20:1(11Z)/P-18:1(11Z))	$C_{43}H_{82}NO_7P$	9.19	755.59	7.79×10^{5}	9.53×10^{5}	7.03×10^{5}	5.10×10^{5}	4.68×10^{5}	4.89×10^{5}	0.60	-0.73	1.26×10^{-2}	1.00	1.50	down	Confident compound
C1864	Irganox 1010 degradation product C16H22O2	$C_{16}H_{22}O_2$	4.89	246.16	4.20×10^{5}	1.00×10^{6}	1.01×10^{6}	3.35×10^{5}	2.21×10^{5}	2.14×10^{5}	0.32	-1.66	4.99×10^{-2}	1.00	1.32	down	Suspected contaminant
C1865	Aniline Yellow	$C_{12}H_{11}N_3$	3.71	197.10	4.93×10^{5}	6.49×10^{5}	8.57×10^{5}	3.75×10^{5}	1.27×10^{5}	1.30×10^{5}	0.32	-1.66	2.71×10^{-2}	1.00	1.41	down	Suspected contaminant

ID	Description	Formula	RT (min)	m/z	Model1	Model2	Model3	FCDPE1	FCDPE2	FCDPE3	FC	log2FC	P value	ROC	VIP	Up/Down	Annotation category
C1869	1,2-Dihydroxyheptadec-16-yn-4-one	$C_{17}H_{30}O_3$	7.93	282.22	5.20×10^{5}	7.81×10^{5}	6.49×10^{5}	3.91×10^{5}	3.96×10^{5}	3.77×10^{5}	0.60	-0.74	2.60×10^{-2}	1.00	1.41	down	Confident compound
C1877	SVEGOFXIARNYNN-VOTSOKGWSA-N	$C_{16}H_{26}O_5$	4.42	298.18	9.40×10^{5}	6.13×10^{5}	9.77×10^{5}	5.54×10^{5}	2.36×10^{5}	2.23×10^{5}	0.40	-1.32	3.31×10^{-2}	1.00	1.40	down	Confident compound
C1889	1,4-Anhydro-5-[(cyclopentylcarbonyl)amino]-2,5-dideoxy-2-[(1-methyl-4-piperidinyl)amino]-D-arabinitol	$C_{17}H_{31}N_3O_3$	6.80	325.23	4.88×10^{5}	6.13×10^{5}	9.30×10^{5}	2.97×10^{5}	2.28×10^{5}	1.57×10^{5}	0.34	-1.58	3.07×10^{-2}	1.00	1.39	down	Suspected contaminant
C1975	Glu-Val-Asn	$C_{14}H_{24}N_4O_7$	3.58	360.17	3.64×10^{5}	4.00×10^{5}	1.88×10^{5}	6.06×10^{4}	1.00×10^{5}	9.87×10^{4}	0.27	-1.88	2.57×10^{-2}	1.00	1.43	down	Confident compound
C2011	Diisononyl adipate	$C_{24}H_{46}O_4$	9.85	398.34	4.10×10^{7}	3.45×10^{7}	2.15×10^{7}	1.51×10^{7}	7.82×10^{6}	6.90×10^{6}	0.31	-1.71	2.35×10^{-2}	1.00	1.45	down	Suspected contaminant
C2017	Succinic acid	$C_4H_6O_4$	0.83	118.03	7.41×10^{8}	1.14×10^{9}	1.77×10^{9}	3.36×10^{8}	1.29×10^{8}	1.69×10^{8}	0.17	-2.52	3.05×10^{-2}	1.00	1.39	down	Confident compound
C2076	Azelaic acid	$C_9H_{16}O_4$	4.21	188.10	1.04×10^{8}	1.23×10^{8}	1.55×10^{8}	9.19×10^{7}	6.74×10^{7}	6.09×10^{7}	0.58	-0.79	3.74×10^{-2}	1.00	1.37	down	Confident compound
C2077	11(E)-Eicosenoic Acid	$C_{20}H_{38}O_2$	9.82	310.29	1.10×10^{8}	9.52×10^{7}	1.22×10^{8}	8.63×10^{7}	6.57×10^{7}	6.56×10^{7}	0.66	-0.59	2.43×10^{-2}	1.00	1.43	down	Confident compound
C2078	2-Butoxyethyl (9E)-9-octadecenoate	$C_{24}H_{46}O_3$	10.57	382.34	9.42×10^{7}	1.20×10^{8}	9.73×10^{7}	6.41×10^{7}	4.29×10^{7}	4.88×10^{7}	0.50	-1.00	7.29×10^{-3}	1.00	1.53	down	Suspected contaminant
C2091	PI-Cer 12:0;2O/23:0;O	$C_{41}H_{82}NO_{12}P$	13.07	811.56	1.23×10^{8}	1.22×10^{8}	9.68×10^{7}	4.02×10^{7}	4.79×10^{7}	5.27×10^{7}	0.41	-1.28	1.96×10^{-3}	1.00	1.59	down	Confident compound
C2121	1,3-Dioxolane	$C_3H_6O_2$	0.84	74.04	1.79×10^{7}	2.40×10^{7}	3.78×10^{7}	8.06×10^{6}	3.49×10^{6}	4.98×10^{6}	0.21	-2.27	2.52×10^{-2}	1.00	1.41	down	Suspected contaminant
C2163	D&C Red Dye 27	$C_{20}H_4Br_4Cl_4O_5$	11.37	779.56	2.70×10^{7}	4.13×10^{7}	2.52×10^{7}	6.93×10^{6}	1.09×10^{7}	8.78×10^{6}	0.29	-1.81	1.29×10^{-2}	1.00	1.49	down	Suspected contaminant
C2170	PI-Cer 12:0;2O/21:0;O	$C_{39}H_{78}NO_{12}P$	13.08	783.53	6.12×10^{7}	4.54×10^{7}	3.93×10^{7}	1.57×10^{7}	1.70×10^{7}	1.72×10^{7}	0.34	-1.55	8.04×10^{-3}	1.00	1.54	down	Confident compound
C2205	4-Hydroxy-3-methoxyphenylglycol sulfate	$C_9H_{12}O_7S$	3.28	264.03	1.97×10^{6}	6.09×10^{5}	1.26×10^{6}	6.81×10^{4}	5.83×10^{4}	4.49×10^{4}	0.04	-4.49	3.60×10^{-2}	1.00	1.39	down	Confident compound
C2206	3-[3-(Sulfooxy)phenyl]propanoic acid	$C_9H_{10}O_6S$	3.28	246.02	1.97×10^{6}	6.26×10^{5}	1.26×10^{6}	6.81×10^{4}	5.45×10^{4}	5.28×10^{4}	0.05	-4.46	3.44×10^{-2}	1.00	1.40	down	Confident compound
C2245	NAGlySer 15:0/17:0	$C_{37}H_{70}N_2O_7$	12.51	654.52	3.22×10^{7}	3.02×10^{7}	2.00×10^{7}	6.72×10^{6}	1.03×10^{7}	7.87×10^{6}	0.30	-1.72	8.18×10^{-3}	1.00	1.53	down	Confident compound
C2249	NP-018716	$C_{11}H_{20}O_4$	4.98	216.14	2.12×10^{7}	1.38×10^{7}	1.63×10^{7}	6.04×10^{6}	4.34×10^{6}	4.69×10^{6}	0.29	-1.77	5.60×10^{-3}	1.00	1.55	down	Confident compound
C2266	NAGlySer 15:0/16:0	$C_{36}H_{68}N_2O_7$	12.01	640.50	3.63×10^{7}	2.71×10^{7}	2.47×10^{7}	7.16×10^{6}	1.47×10^{7}	1.03×10^{7}	0.37	-1.45	1.11×10^{-2}	1.00	1.50	down	Confident compound
C2283	IPIKCTWGPWLVBN-FGYSCDCISA-N	$C_{30}H_{44}O_5$	6.94	484.32	6.56×10^{6}	1.26×10^{7}	1.52×10^{7}	5.27×10^{6}	2.64×10^{6}	1.62×10^{6}	0.28	-1.85	4.11×10^{-2}	1.00	1.35	down	Confident compound
C2319	13Z,16Z-Docosadienoic Acid	$C_{22}H_{40}O_2$	9.88	336.30	7.78×10^{6}	5.92×10^{6}	5.86×10^{6}	3.33×10^{6}	3.27×10^{6}	3.76×10^{6}	0.53	-0.92	9.12×10^{-3}	1.00	1.53	down	Confident compound
C2336	PI-Cer 12:0;2O/24:0;O	$C_{42}H_{84}NO_{12}P$	13.06	825.57	1.80×10^{7}	2.16×10^{7}	1.86×10^{7}	6.87×10^{6}	5.91×10^{6}	3.67×10^{6}	0.28	-1.82	6.93×10^{-4}	1.00	1.61	down	Confident compound
C2391	LNAPE 22:0/N-2:0	$C_{29}H_{58}NO_8P$	9.26	579.39	4.28×10^{6}	2.80×10^{6}	4.69×10^{6}	2.61×10^{6}	7.79×10^{5}	7.64×10^{5}	0.35	-1.50	3.89×10^{-2}	1.00	1.38	down	Confident compound
C2454	SL 18:1;O/15:0	$C_{33}H_{65}NO_5S$	9.66	587.46	5.29×10^{6}	4.94×10^{6}	3.65×10^{6}	3.50×10^{6}	2.16×10^{6}	1.55×10^{6}	0.52	-0.95	4.35×10^{-2}	1.00	1.38	down	Confident compound
C2468	5-Methoxy-2-{[(2S)-2-{3-[2-(2-methoxyethoxy)-3-pyridinyl]-1,2,4-oxadiazol-5-yl}-1-pyrrolidinyl]methyl}phenol	$C_{22}H_{26}N_4O_5$	4.23	426.19	6.53×10^{6}	7.48×10^{6}	1.06×10^{7}	4.36×10^{6}	1.86×10^{6}	7.96×10^{5}	0.28	-1.81	2.25×10^{-2}	1.00	1.43	down	Suspected contaminant
C2509	LNAPE 15:0/N-2:0	$C_{22}H_{44}NO_8P$	6.66	481.28	7.39×10^{6}	5.13×10^{6}	7.72×10^{6}	2.62×10^{6}	3.44×10^{6}	2.31×10^{6}	0.41	-1.28	1.08×10^{-2}	1.00	1.50	down	Confident compound

ID	Description	Formula	RT (min)	m/z	Model1	Model2	Model3	FCDPE1	FCDPE2	FCDPE3	FC	log2FC	P value	ROC	VIP	Up/Down	Annotation category
C2519	MG(0:0/20:0/0:0)	$C_{23}H_{46}O_4$	10.17	386.34	7.84×10^{6}	5.68×10^{6}	5.29×10^{6}	3.46×10^{6}	2.52×10^{6}	2.42×10^{6}	0.45	-1.16	1.57×10^{-2}	1.00	1.49	down	Confident compound
C2590	KQGVEZGOGSMUMX-KTKRTIGZSA-N	$C_{25}H_{42}O_2$	9.82	374.32	7.42×10^{6}	9.35×10^{6}	5.57×10^{6}	4.57×10^{6}	2.77×10^{6}	2.41×10^{6}	0.44	-1.20	3.03×10^{-2}	1.00	1.42	down	Confident compound
C2661	NAGlySer 14:0/16:0	$C_{35}H_{66}N_2O_7$	11.58	626.49	7.58×10^{6}	5.34×10^{6}	3.86×10^{6}	1.03×10^{6}	2.46×10^{6}	2.53×10^{6}	0.36	-1.48	3.90×10^{-2}	1.00	1.38	down	Confident compound
C2701	Gly-Asp-Gln	$C_{11}H_{18}N_4O_7$	4.01	318.11	1.94×10^{6}	1.78×10^{6}	2.06×10^{6}	1.29×10^{6}	1.08×10^{6}	1.04×10^{6}	0.59	-0.76	2.22×10^{-3}	1.00	1.58	down	Confident compound
C2708	LPE O-18:1	$C_{23}H_{48}NO_6P$	8.26	465.32	2.27×10^{6}	3.26×10^{6}	2.49×10^{6}	1.23×10^{5}	6.17×10^{5}	3.25×10^{5}	0.13	-2.91	2.22×10^{-3}	1.00	1.57	down	Confident compound
C2709	NFPZOORPDJBGME-UMSAECHCSA-N	$C_{30}H_{48}O_6$	5.17	504.35	6.56×10^{6}	3.74×10^{6}	2.97×10^{6}	9.62×10^{5}	1.21×10^{6}	1.44×10^{6}	0.27	-1.88	4.28×10^{-2}	1.00	1.38	down	Confident compound
C2726	NP-017624	$C_{23}H_{32}O_8$	4.31	436.21	2.14×10^{6}	2.16×10^{6}	2.90×10^{6}	1.41×10^{6}	1.08×10^{6}	7.63×10^{5}	0.45	-1.14	1.34×10^{-2}	1.00	1.48	down	Confident compound
C2739	N-(4-Fluorobenzyl)-2-[(1S,4S,5S)-5-isopropyl-2-methyl-4-{[(propylsulfonyl)amino]methyl}-2-cyclohexen-1-yl]acetamide	$C_{23}H_{35}FN_2O_3S$	4.16	438.23	1.35×10^{6}	1.08×10^{6}	1.28×10^{6}	8.08×10^{5}	6.16×10^{5}	6.02×10^{5}	0.54	-0.88	5.67×10^{-3}	1.00	1.55	down	Suspected contaminant
C2779	Acoric acid	$C_{15}H_{24}O_4$	5.47	268.17	2.37×10^{6}	2.21×10^{6}	1.99×10^{6}	1.24×10^{6}	7.30×10^{5}	6.90×10^{5}	0.40	-1.31	3.26×10^{-3}	1.00	1.58	down	Confident compound
C2877	Myristic acid alkyne	$C_{14}H_{24}O_2$	6.25	224.18	3.54×10^{6}	2.43×10^{6}	1.51×10^{6}	6.24×10^{5}	6.91×10^{5}	5.17×10^{5}	0.24	-2.03	3.28×10^{-2}	1.00	1.41	down	Suspected contaminant
C2895	PE O-16:4_15:0	$C_{36}H_{66}NO_7P$	12.41	655.45	2.23×10^{6}	1.84×10^{6}	2.07×10^{6}	2.71×10^{5}	4.28×10^{5}	3.54×10^{5}	0.17	-2.54	1.66×10^{-4}	1.00	1.63	down	Confident compound
C2904	OBDMHMQEFHPZHD-QPJJXVBHSA-N	$C_{15}H_{16}O_6$	4.13	292.10	1.29×10^{6}	8.23×10^{5}	1.25×10^{6}	3.57×10^{5}	6.04×10^{5}	4.49×10^{5}	0.42	-1.25	1.72×10^{-2}	1.00	1.46	down	Confident compound
C2978	15-Crown-5,1,4,7,10,13-Pentaoxa-cyclo-pentadecane	$C_{10}H_{20}O_5$	3.86	220.13	2.12×10^{6}	1.39×10^{6}	1.79×10^{6}	9.98×10^{5}	7.31×10^{5}	8.19×10^{5}	0.48	-1.06	1.48×10^{-2}	1.00	1.49	down	Suspected contaminant
C2981	SHexCer 42:0;3O	$C_{48}H_{95}NO_{12}S$	11.34	909.66	2.14×10^{6}	2.25×10^{6}	2.50×10^{6}	3.48×10^{5}	6.93×10^{5}	7.71×10^{5}	0.26	-1.93	5.30×10^{-4}	1.00	1.61	down	Confident compound
C2987	3,5,7-Trihydroxy-4-methoxyflavanone	$C_{16}H_{14}O_6$	5.02	302.08	8.26×10^{5}	1.09×10^{6}	1.31×10^{6}	4.92×10^{5}	4.81×10^{5}	5.03×10^{5}	0.46	-1.13	1.43×10^{-2}	1.00	1.47	down	Confident compound
C3006	SHexCer 34:0;3O	$C_{40}H_{79}NO_{12}S$	9.74	797.53	1.24×10^{6}	1.40×10^{6}	1.49×10^{6}	7.83×10^{5}	8.81×10^{5}	7.70×10^{5}	0.59	-0.76	2.18×10^{-3}	1.00	1.57	down	Confident compound
C3037	2,4,8,10-Tetraoxaspiro[5.5]undecane-3,9-diylbis-2-methyl-2,1-propanediyl bis{3-[4-hydroxy-3-methyl-5-(2-methyl-2-propanyl)phenyl]propanoate}	$C_{43}H_{64}O_{10}$	9.50	740.45	2.36×10^{6}	2.18×10^{6}	1.90×10^{6}	3.76×10^{5}	3.25×10^{5}	4.17×10^{5}	0.17	-2.52	2.07×10^{-4}	1.00	1.63	down	Suspected contaminant
C3055	SHexCer 40:0;3O	$C_{46}H_{91}NO_{12}S$	10.74	881.63	1.27×10^{6}	1.40×10^{6}	1.95×10^{6}	3.72×10^{5}	8.41×10^{5}	8.25×10^{5}	0.44	-1.18	2.95×10^{-2}	1.00	1.38	down	Confident compound
C3056	LPI 20:3	$C_{29}H_{51}O_{12}P$	6.68	622.31	9.42×10^{5}	6.76×10^{5}	8.46×10^{5}	3.36×10^{5}	5.78×10^{5}	3.83×10^{5}	0.53	-0.93	2.24×10^{-2}	1.00	1.43	down	Confident compound
C3082	Cer 17:0;2O/16:0;(3OH)	$C_{33}H_{67}NO_4$	11.71	541.51	1.40×10^{6}	8.50×10^{5}	8.95×10^{5}	2.54×10^{5}	3.11×10^{5}	3.29×10^{5}	0.28	-1.81	1.31×10^{-2}	1.00	1.50	down	Confident compound
C3144	3-[(4,6-dimethylpyrimidin-2-yl)amino]-2-[(2-methyl-3-furyl)carbonyl]acrylonitrile	$C_{15}H_{14}N_4O_2$	3.35	282.11	7.77×10^{5}	7.68×10^{5}	1.06×10^{6}	5.75×10^{5}	3.40×10^{5}	2.02×10^{5}	0.43	-1.22	2.69×10^{-2}	1.00	1.42	down	Suspected contaminant
C3204	Cer 17:0;2O/15:0;(3OH)	$C_{32}H_{65}NO_4$	11.29	527.49	8.34×10^{5}	5.31×10^{5}	5.89×10^{5}	9.84×10^{4}	2.83×10^{5}	3.47×10^{5}	0.37	-1.42	2.64×10^{-2}	1.00	1.43	down	Confident compound
C3225	SHexCer 40:1;3O	$C_{46}H_{89}NO_{12}S$	10.83	879.61	5.65×10^{5}	6.76×10^{5}	7.54×10^{5}	1.39×10^{5}	3.23×10^{5}	3.29×10^{5}	0.40	-1.34	8.45×10^{-3}	1.00	1.50	down	Confident compound
C3277	Irganox 1010 degradation product C41H60O10	$C_{41}H_{60}O_{10}$	8.96	712.42	7.17×10^{5}	7.59×10^{5}	6.62×10^{5}	4.85×10^{4}	6.47×10^{4}	6.93×10^{4}	0.09	-3.55	2.31×10^{-5}	1.00	1.64	down	Suspected contaminant
C3309	SHexCer 44:0;3O	$C_{50}H_{99}NO_{12}S$	12.20	937.69	2.52×10^{5}	3.69×10^{5}	2.82×10^{5}	4.97×10^{4}	1.55×10^{5}	1.44×10^{5}	0.39	-1.37	1.87×10^{-2}	1.00	1.44	down	Confident compound

ID	Description	Formula	RT (min)	m/z	Model1	Model2	Model3	FCDPE1	FCDPE2	FCDPE3	FC	log2FC	P value	ROC	VIP	Up/Down	Annotation category
C1	1-(4-nitrophenyl)piperidine	$C_{11}H_{14}N_2O_2$	2.88	206.11	2.07×10^{9}	1.09×10^{9}	1.07×10^{9}	4.19×10^{9}	2.82×10^{9}	3.03×10^{9}	2.37	1.24	2.31×10^{-2}	1.00	1.41	up	Suspected contaminant
C17	3-methyl-3,4-dihydro-1H-1,4-benzodiazepine-2,5-dione	$C_{10}H_{10}N_2O_2$	0.96	190.08	4.37×10^{8}	1.54×10^{8}	1.80×10^{8}	6.64×10^{8}	5.75×10^{8}	9.43×10^{8}	2.83	1.50	3.01×10^{-2}	1.00	1.39	up	Suspected contaminant
C26	3,4-Tetramethylene-α-pyrrolidinobutiophenone	$C_{18}H_{25}NO$	3.36	271.19	6.68×10^{7}	3.08×10^{7}	4.22×10^{7}	4.63×10^{8}	7.16×10^{8}	7.70×10^{8}	13.94	3.80	3.15×10^{-3}	1.00	1.57	up	Suspected contaminant
C28	N6-Acetyl-L-lysine	$C_8H_{16}N_2O_3$	0.77	188.12	2.67×10^{8}	7.16×10^{7}	7.99×10^{7}	3.51×10^{8}	2.99×10^{8}	4.90×10^{8}	2.73	1.45	4.82×10^{-2}	1.00	1.32	up	Confident compound
C35	2-{2-[2-(Octanoyloxy)ethoxy]ethoxy}ethyl decanoate	$C_{24}H_{46}O_6$	7.53	430.33	4.65×10^{7}	2.67×10^{7}	3.36×10^{7}	7.58×10^{7}	1.30×10^{8}	1.70×10^{8}	3.52	1.82	3.25×10^{-2}	1.00	1.40	up	Suspected contaminant
C66	Tryptophyl-Valine	$C_{16}H_{21}N_3O_3$	3.44	303.16	3.20×10^{7}	5.78×10^{7}	4.96×10^{7}	1.24×10^{8}	8.33×10^{7}	9.46×10^{7}	2.16	1.11	1.90×10^{-2}	1.00	1.45	up	Confident compound
C79	Ultranox 626 degradation product C33H50O8P2	$C_{33}H_{50}O_8P_2$	8.54	636.30	8.09×10^{7}	5.29×10^{7}	4.46×10^{7}	1.24×10^{8}	1.45×10^{8}	1.33×10^{8}	2.26	1.17	4.01×10^{-3}	1.00	1.55	up	Suspected contaminant
C83	1-(4-{[(1S,4S,6S)-6-Isopropyl-3-methyl-4-{[5-(4-pyridinyl)-1,3,4-oxadiazol-2-yl]methyl}-2-cyclohexen-1-yl]methyl}-1-piperazinyl)ethanone	$C_{25}H_{35}N_5O_2$	6.57	437.28	6.76×10^{7}	7.39×10^{7}	5.00×10^{7}	9.19×10^{7}	1.22×10^{8}	1.13×10^{8}	1.71	0.77	1.71×10^{-2}	1.00	1.47	up	Suspected contaminant
C87	2-Amino-1,3-octadecanediol	$C_{18}H_{39}NO_2$	6.68	301.30	7.78×10^{7}	5.13×10^{7}	5.01×10^{7}	9.47×10^{7}	1.47×10^{8}	1.16×10^{8}	2.00	1.00	2.83×10^{-2}	1.00	1.42	up	Confident compound
C102	Lysylproline	$C_{11}H_{21}N_3O_3$	2.91	243.16	1.79×10^{7}	6.24×10^{6}	1.29×10^{7}	7.87×10^{7}	1.85×10^{8}	1.65×10^{8}	11.58	3.53	1.64×10^{-2}	1.00	1.48	up	Confident compound
C103	3-Vinylaniline	C_8H_9N	2.88	119.07	3.00×10^{7}	1.50×10^{7}	1.83×10^{7}	6.45×10^{7}	3.79×10^{7}	4.80×10^{7}	2.38	1.25	3.18×10^{-2}	1.00	1.37	up	Suspected contaminant
C116	2-(Cyclohexylmethylidene)-1,2,3,4-tetrahydronaphthalen-1-one	$C_{17}H_{20}O$	3.74	240.15	2.76×10^{7}	1.64×10^{7}	1.91×10^{7}	4.18×10^{7}	6.64×10^{7}	5.52×10^{7}	2.59	1.37	1.32×10^{-2}	1.00	1.50	up	Confident compound
C118	Phenyltoloxamine	$C_{17}H_{21}NO$	2.06	255.16	2.83×10^{7}	1.65×10^{7}	2.03×10^{7}	6.20×10^{7}	9.76×10^{7}	1.07×10^{8}	4.10	2.03	9.04×10^{-3}	1.00	1.52	up	Suspected contaminant
C119	Glu-Leu	$C_{11}H_{20}N_2O_5$	2.90	260.14	3.09×10^{7}	8.84×10^{6}	1.88×10^{7}	4.28×10^{7}	1.03×10^{8}	8.59×10^{7}	3.95	1.98	3.85×10^{-2}	1.00	1.39	up	Confident compound
C126	Rhodamine 6G	$C_{28}H_{30}N_2O_3$	6.26	442.23	1.28×10^{5}	7.83×10^{4}	3.10×10^{5}	3.93×10^{5}	3.94×10^{5}	3.40×10^{5}	2.18	1.13	4.84×10^{-2}	1.00	1.35	up	Suspected contaminant
C130	PPK	$C_{16}H_{28}N_4O_4$	3.20	340.21	2.65×10^{7}	8.49×10^{6}	1.53×10^{7}	7.15×10^{7}	1.20×10^{8}	1.03×10^{8}	5.85	2.55	5.78×10^{-3}	1.00	1.55	up	Confident compound
C137	1,4-Anhydro-2,5-dideoxy-5-[(3,3-dimethylbutanoyl)amino]-2-(3-oxetanylamino)-D-arabinitol	$C_{14}H_{26}N_2O_4$	4.45	286.19	1.16×10^{7}	1.08×10^{6}	7.45×10^{6}	2.94×10^{7}	1.76×10^{7}	4.04×10^{7}	4.34	2.12	3.67×10^{-2}	1.00	1.36	up	Suspected contaminant
C143	Difenzoquat	$C_{17}H_{16}N_2$	0.96	248.13	5.67×10^{7}	1.99×10^{7}	1.36×10^{7}	8.31×10^{7}	8.45×10^{7}	6.31×10^{7}	2.56	1.35	3.64×10^{-2}	1.00	1.37	up	Suspected contaminant
C156	N-Arachidonoyl taurine	$C_{22}H_{37}NO_4S$	3.25	411.25	8.46×10^{6}	1.29×10^{6}	8.39×10^{6}	4.73×10^{7}	1.11×10^{8}	9.20×10^{7}	13.81	3.79	1.52×10^{-2}	1.00	1.49	up	Confident compound
C170	(4aS,9aR)-2-[(4-Methoxyphenyl)acetyl]-7-[2-(4-morpholinyl)ethyl]decahydro-6H-pyrido[3,4-d]azepin-6-one	$C_{24}H_{35}N_3O_4$	3.23	429.26	6.49×10^{6}	3.30×10^{6}	5.08×10^{6}	3.76×10^{7}	9.27×10^{7}	6.29×10^{7}	12.99	3.70	2.04×10^{-2}	1.00	1.47	up	Suspected contaminant
C202	Ethyltriglycol	$C_8H_{18}O_4$	2.07	178.12	1.31×10^{6}	2.23×10^{5}	2.67×10^{5}	3.97×10^{6}	4.47×10^{6}	4.88×10^{6}	7.38	2.88	9.84×10^{-4}	1.00	1.60	up	Suspected contaminant
C209	6β-Hydrocodol	$C_{18}H_{23}NO_3$	3.10	301.16	2.14×10^{7}	1.24×10^{7}	2.71×10^{7}	4.86×10^{7}	6.32×10^{7}	4.44×10^{7}	2.57	1.36	1.12×10^{-2}	1.00	1.51	up	Suspected contaminant
C210	NP-020439	$C_{11}H_{18}N_2O_4$	3.54	242.13	1.77×10^{7}	8.35×10^{6}	1.21×10^{7}	3.12×10^{7}	5.70×10^{7}	4.38×10^{7}	3.46	1.79	1.69×10^{-2}	1.00	1.48	up	Confident compound
C234	LPC 20:4-SN1	$C_{28}H_{50}NO_7P$	6.89	543.33	1.09×10^{7}	5.58×10^{6}	6.87×10^{6}	2.64×10^{7}	2.02×10^{7}	1.61×10^{7}	2.69	1.43	1.80×10^{-2}	1.00	1.45	up	Confident compound
C245	Pro-Pro	$C_{10}H_{16}N_2O_3$	3.09	212.12	1.66×10^{7}	1.07×10^{7}	1.24×10^{7}	2.64×10^{7}	4.43×10^{7}	3.58×10^{7}	2.68	1.42	1.50×10^{-2}	1.00	1.49	up	Confident compound

ID	Description	Formula	RT (min)	m/z	Model1	Model2	Model3	FCDPE1	FCDPE2	FCDPE3	FC	log2FC	P value	ROC	VIP	Up/Down	Annotation category
C274	SLK	$C_{15}H_{30}N_4O_5$	3.45	346.22	2.94×10^{7}	2.59×10^{6}	7.30×10^{6}	3.57×10^{7}	4.12×10^{7}	3.76×10^{7}	2.92	1.54	4.07×10^{-2}	1.00	1.35	up	Confident compound
C277	(S)-[(1S,2R,4S,5R)-5-ethyl-1-azabicyclo[2.2.2]octan-2-yl](6-methoxyquinolin-4-yl)methanol	$C_{20}H_{26}N_2O_2$	1.48	326.20	8.55×10^{6}	3.96×10^{6}	4.15×10^{6}	1.34×10^{7}	2.49×10^{7}	1.49×10^{7}	3.19	1.67	3.61×10^{-2}	1.00	1.39	up	Suspected contaminant
C289	LPK	$C_{17}H_{32}N_4O_4$	3.83	356.24	7.81×10^{6}	1.28×10^{6}	8.73×10^{6}	3.35×10^{7}	4.49×10^{7}	3.99×10^{7}	6.63	2.73	1.17×10^{-3}	1.00	1.61	up	Confident compound
C293	Ajmaline	$C_{20}H_{26}N_2O_2$	3.37	326.20	1.22×10^{7}	6.14×10^{6}	3.80×10^{6}	2.63×10^{7}	1.82×10^{7}	2.25×10^{7}	3.02	1.60	1.22×10^{-2}	1.00	1.47	up	Suspected contaminant
C308	Glu-Val-Leu	$C_{16}H_{29}N_3O_6$	3.58	359.21	6.06×10^{6}	2.23×10^{6}	4.58×10^{6}	2.29×10^{7}	3.39×10^{7}	2.74×10^{7}	6.54	2.71	2.20×10^{-3}	1.00	1.59	up	Confident compound
C314	N1-[4-(2-nitrovinyl)phenyl]acetamide	$C_{10}H_{10}N_2O_3$	1.10	206.07	1.11×10^{7}	6.00×10^{6}	5.33×10^{6}	2.02×10^{7}	2.14×10^{7}	2.38×10^{7}	2.92	1.55	2.35×10^{-3}	1.00	1.57	up	Suspected contaminant
C316	N-Acetyl-DL-tryptophan	$C_{13}H_{14}N_2O_3$	3.06	246.10	6.09×10^{6}	1.66×10^{6}	3.32×10^{6}	2.96×10^{7}	2.86×10^{7}	2.46×10^{7}	7.48	2.90	2.83×10^{-4}	1.00	1.62	up	Confident compound
C331	CAY10401	$C_{24}H_{34}N_2O_2$	3.96	382.26	3.15×10^{6}	7.31×10^{5}	9.81×10^{5}	1.81×10^{7}	4.47×10^{7}	4.59×10^{7}	22.35	4.48	1.90×10^{-2}	1.00	1.47	up	Suspected contaminant
C333	Norbuprenorphine	$C_{25}H_{35}NO_4$	3.80	413.26	3.18×10^{6}	4.66×10^{5}	5.50×10^{6}	2.81×10^{7}	3.57×10^{7}	3.14×10^{7}	10.41	3.38	3.95×10^{-4}	1.00	1.62	up	Suspected contaminant
C334	GPK	$C_{13}H_{24}N_4O_4$	2.83	300.18	6.21×10^{6}	1.62×10^{6}	3.42×10^{6}	1.29×10^{7}	4.43×10^{7}	3.79×10^{7}	8.45	3.08	4.47×10^{-2}	1.00	1.37	up	Confident compound
C335	Tri-o-Cresyl phosphate	$C_{21}H_{21}O_4P$	7.87	368.12	6.38×10^{6}	7.42×10^{6}	7.83×10^{6}	2.81×10^{7}	1.53×10^{7}	1.59×10^{7}	2.74	1.45	4.04×10^{-2}	1.00	1.35	up	Suspected contaminant
C341	Gly-Met	$C_7H_{14}N_2O_3S$	0.55	206.07	9.24×10^{6}	5.92×10^{6}	2.93×10^{6}	2.33×10^{7}	2.03×10^{7}	2.28×10^{7}	3.67	1.88	1.41×10^{-3}	1.00	1.58	up	Confident compound
C346	5-octadecyloxyisophthalic acid	$C_{26}H_{42}O_5$	6.55	434.30	8.54×10^{6}	1.41×10^{7}	8.14×10^{6}	1.74×10^{7}	2.30×10^{7}	1.94×10^{7}	1.94	0.96	1.87×10^{-2}	1.00	1.47	up	Suspected contaminant
C353	NP-023108	$C_{15}H_{17}NO_2$	2.97	243.13	7.60×10^{6}	1.77×10^{6}	4.46×10^{6}	1.54×10^{7}	3.42×10^{7}	2.90×10^{7}	5.68	2.51	2.09×10^{-2}	1.00	1.46	up	Confident compound
C357	SPK	$C_{14}H_{26}N_4O_5$	1.36	330.19	1.04×10^{6}	1.16×10^{6}	1.23×10^{6}	1.25×10^{7}	3.91×10^{7}	3.45×10^{7}	25.09	4.65	2.86×10^{-2}	1.00	1.43	up	Confident compound
C367	{(3R,4S)-1-(Cyclopropylcarbonyl)-3-[2-(4-phenyl-1-piperazinyl)ethyl]-4-piperidinyl}acetic acid	$C_{23}H_{33}N_3O_3$	3.44	399.25	3.59×10^{6}	8.52×10^{5}	3.83×10^{6}	1.25×10^{7}	2.92×10^{7}	2.71×10^{7}	8.32	3.06	1.93×10^{-2}	1.00	1.47	up	Suspected contaminant
C387	Lys-Val-Ser	$C_{14}H_{28}N_4O_5$	3.12	332.21	4.04×10^{6}	9.34×10^{5}	2.21×10^{6}	2.18×10^{7}	3.49×10^{7}	3.27×10^{7}	12.45	3.64	2.74×10^{-3}	1.00	1.58	up	Confident compound
C390	Valylvaline	$C_{10}H_{20}N_2O_3$	3.04	216.15	7.65×10^{6}	5.00×10^{6}	4.46×10^{6}	9.82×10^{6}	2.39×10^{7}	1.92×10^{7}	3.09	1.63	4.84×10^{-2}	1.00	1.35	up	Confident compound
C400	RNK	$C_{16}H_{32}N_8O_5$	4.15	416.24	1.58×10^{6}	4.67×10^{5}	3.12×10^{6}	1.33×10^{7}	1.91×10^{7}	1.78×10^{7}	9.71	3.28	1.46×10^{-3}	1.00	1.60	up	Confident compound
C423	{(3R,4S)-1-(Cyclobutylcarbonyl)-3-[2-(4-phenyl-1-piperazinyl)ethyl]-4-piperidinyl}acetic acid	$C_{24}H_{35}N_3O_3$	3.03	413.26	2.46×10^{6}	4.88×10^{5}	1.96×10^{6}	1.18×10^{7}	1.64×10^{7}	2.84×10^{7}	11.54	3.53	2.60×10^{-2}	1.00	1.42	up	Suspected contaminant
C424	NP-016141	$C_{22}H_{29}NO_3$	3.71	355.21	3.74×10^{6}	1.25×10^{6}	4.12×10^{6}	8.21×10^{6}	1.61×10^{7}	8.95×10^{6}	3.65	1.87	3.96×10^{-2}	1.00	1.39	up	Confident compound
C431	Cyclo(Ile-Ala)	$C_9H_{16}N_2O_2$	3.48	184.12	1.15×10^{7}	5.96×10^{6}	1.21×10^{7}	1.49×10^{7}	1.90×10^{7}	1.56×10^{7}	1.67	0.74	4.70×10^{-2}	1.00	1.36	up	Confident compound
C432	2-[2-(2-acetamido-4-methylpentanamido)propanamido]-3-hydroxypropanoic acid	$C_{14}H_{25}N_3O_6$	3.34	331.17	4.15×10^{5}	1.38×10^{6}	3.08×10^{6}	4.76×10^{6}	1.33×10^{7}	1.17×10^{7}	6.11	2.61	3.86×10^{-2}	1.00	1.40	up	Confident compound
C433	5-[3-(Butylamino)-2-hydroxypropoxy]-1,2,3,4-tetrahydro-2,3-naphthalenediol	$C_{17}H_{27}NO_4$	3.35	309.19	4.15×10^{5}	4.45×10^{5}	1.92×10^{6}	4.76×10^{6}	1.33×10^{7}	1.17×10^{7}	10.71	3.42	2.80×10^{-2}	1.00	1.43	up	Suspected contaminant
C456	Indoline	C_8H_9N	3.21	119.07	5.01×10^{6}	4.21×10^{6}	6.39×10^{6}	1.29×10^{7}	1.12×10^{7}	1.30×10^{7}	2.38	1.25	1.22×10^{-3}	1.00	1.59	up	Confident compound

ID	Description	Formula	RT (min)	m/z	Model1	Model2	Model3	FCDPE1	FCDPE2	FCDPE3	FC	log2FC	P value	ROC	VIP	Up/Down	Annotation category
C470	1-[(1S,2S,3S,4R,5R)-4-(4-Benzyl-1-piperidinyl)-3-hydroxy-6,8-dioxabicyclo[3.2.1]oct-2-yl]-3-cyclohexylurea	$C_{25}H_{37}N_3O_4$	2.75	443.27	7.47×10^5	3.72×10^5	5.89×10^5	6.96×10^6	1.96×10^7	1.50×10^7	24.32	4.60	2.27×10^{-2}	1.00	1.45	up	Suspected contaminant
C476	Leucine	$C_6H_{13}NO_2$	3.65	131.09	8.83×10^6	5.70×10^6	7.12×10^6	1.43×10^7	1.51×10^7	1.42×10^7	2.01	1.01	1.53×10^{-3}	1.00	1.59	up	Confident compound
C487	2-[(3S)-1-Benzyl-3-pyrrolidinyl]-1,3-benzothiazole	$C_{18}H_{18}N_2S$	3.22	294.12	3.47×10^6	2.16×10^6	4.41×10^6	1.08×10^7	1.71×10^7	1.36×10^7	4.13	2.05	5.85×10^{-3}	1.00	1.55	up	Suspected contaminant
C491	L-Phenylalanine	$C_9H_{11}NO_2$	2.88	165.08	7.84×10^6	7.70×10^6	7.41×10^6	1.31×10^7	1.17×10^7	1.12×10^7	1.57	0.65	1.54×10^{-3}	1.00	1.58	up	Confident compound
C503	VMK	$C_{16}H_{32}N_4O_4S$	3.97	376.21	5.14×10^6	1.55×10^6	5.49×10^6	1.28×10^7	2.00×10^7	1.74×10^7	4.13	2.05	6.43×10^{-3}	1.00	1.55	up	Confident compound
C517	2-(2-amino-3-methylbutanamido)-3-phenylpropanoic acid	$C_{14}H_{20}N_2O_3$	3.36	264.15	5.81×10^6	3.27×10^6	3.52×10^6	8.55×10^6	1.36×10^7	1.04×10^7	2.58	1.37	1.68×10^{-2}	1.00	1.48	up	Confident compound
C518	Pyroglutamylvaline	$C_{10}H_{16}N_2O_4$	1.96	228.11	6.36×10^6	3.38×10^6	4.34×10^6	1.10×10^7	2.54×10^7	2.02×10^7	4.02	2.01	3.00×10^{-2}	1.00	1.42	up	Confident compound
C520	(1S,2S,3S,4R,5R)-2-Amino-4-[4-(2-methoxyphenyl)-1-piperazinyl]-6,8-dioxabicyclo[3.2.1]octan-3-ol	$C_{17}H_{25}N_3O_4$	3.60	335.18	4.68×10^6	1.44×10^6	2.76×10^6	1.62×10^7	1.77×10^7	1.72×10^7	5.75	2.52	1.76×10^{-4}	1.00	1.63	up	Suspected contaminant
C530	Thr-Ala-Lys	$C_{13}H_{26}N_4O_5$	2.83	318.19	5.56×10^5	4.59×10^5	1.15×10^6	6.50×10^6	1.46×10^7	1.26×10^7	15.61	3.96	1.28×10^{-2}	1.00	1.51	up	Confident compound
C535	Desthiobiotin	$C_{10}H_{18}N_2O_3$	3.00	214.13	6.11×10^6	7.48×10^6	7.75×10^6	9.03×10^6	1.14×10^7	1.34×10^7	1.59	0.67	3.74×10^{-2}	1.00	1.39	up	Confident compound
C544	Nalbuphine	$C_{21}H_{27}NO_4$	4.45	357.19	1.39×10^6	2.93×10^5	8.76×10^5	4.02×10^6	1.48×10^7	1.41×10^7	12.86	3.68	4.42×10^{-2}	1.00	1.37	up	Suspected contaminant
C546	β-Estradiol	$C_{18}H_{24}O_2$	4.36	272.17	1.09×10^6	9.71×10^4	1.56×10^5	1.95×10^6	6.42×10^6	5.51×10^6	10.32	3.37	4.05×10^{-2}	1.00	1.38	up	Confident compound
C558	nylon oligomers amide and group L2	$C_{13}H_{24}O_4N_4$	0.58	300.18	2.57×10^6	8.37×10^5	9.09×10^5	6.12×10^6	1.76×10^7	1.51×10^7	8.97	3.16	3.10×10^{-2}	1.00	1.42	up	Suspected contaminant
C559	Pholcodine	$C_{23}H_{30}N_2O_4$	2.96	398.22	2.92×10^6	2.32×10^6	3.69×10^6	1.07×10^7	1.79×10^7	1.49×10^7	4.87	2.28	5.43×10^{-3}	1.00	1.56	up	Suspected contaminant
C568	Rauwolscine	$C_{21}H_{26}N_2O_3$	3.38	354.19	7.41×10^6	1.49×10^6	3.05×10^6	1.06×10^7	2.10×10^7	2.31×10^7	4.58	2.19	2.87×10^{-2}	1.00	1.42	up	Suspected contaminant
C574	Asn-Val-Leu	$C_{15}H_{28}N_4O_5$	3.38	344.21	1.65×10^6	6.22×10^5	4.53×10^5	5.50×10^6	6.45×10^6	5.32×10^6	6.35	2.67	6.91×10^{-4}	1.00	1.61	up	Confident compound
C576	APK	$C_{14}H_{26}N_4O_4$	2.82	314.20	1.65×10^6	4.83×10^5	1.47×10^6	5.83×10^6	9.03×10^6	9.29×10^6	6.71	2.75	4.27×10^{-3}	1.00	1.56	up	Confident compound
C583	Glu-Pro-Leu	$C_{16}H_{27}N_3O_6$	3.30	357.19	6.65×10^6	3.74×10^6	4.68×10^6	7.87×10^6	1.56×10^7	1.41×10^7	2.49	1.32	4.06×10^{-2}	1.00	1.38	up	Confident compound
C623	Alimemazine	$C_{18}H_{22}N_2S$	5.16	298.15	2.18×10^6	2.92×10^5	1.76×10^6	5.88×10^6	8.85×10^6	6.97×10^6	5.12	2.36	4.97×10^{-3}	1.00	1.56	up	Suspected contaminant
C633	1-Cyclohexyl-3-[(1S,2S,3S,4R,5R)-4-{[2-(dimethylamino)ethyl](methyl)amino}-3-hydroxy-6,8-dioxabicyclo[3.2.1]oct-2-yl]urea	$C_{18}H_{34}N_4O_4$	3.77	370.26	5.96×10^5	3.78×10^5	7.66×10^5	1.20×10^7	1.59×10^7	1.43×10^7	24.23	4.60	2.89×10^{-4}	1.00	1.63	up	Suspected contaminant
C638	Leu-Glu-Thr	$C_{15}H_{27}N_3O_7$	3.32	361.19	1.54×10^6	3.43×10^5	8.36×10^5	4.16×10^6	1.11×10^7	9.90×10^6	9.25	3.21	2.60×10^{-2}	1.00	1.44	up	Confident compound
C639	Gly-Pro-Pro	$C_{12}H_{19}N_3O_4$	2.84	269.14	4.30×10^6	2.27×10^6	3.05×10^6	6.79×10^6	1.64×10^7	1.26×10^7	3.72	1.90	3.81×10^{-2}	1.00	1.39	up	Confident compound
C645	MK-812	$C_{24}H_{34}F_3N_3O_3$	3.35	469.25	1.84×10^6	3.91×10^5	1.36×10^6	6.35×10^6	1.84×10^7	1.35×10^7	10.63	3.41	3.04×10^{-2}	1.00	1.42	up	Suspected contaminant
C666	Isoleucyl-Threonine	$C_{10}H_{20}N_2O_4$	2.04	232.14	3.76×10^6	1.19×10^6	2.13×10^6	4.78×10^6	6.97×10^6	5.27×10^6	2.40	1.26	3.01×10^{-2}	1.00	1.41	up	Confident compound
C691	Ala-Leu-Glu	$C_{14}H_{25}N_3O_6$	2.85	331.17	2.21×10^6	1.26×10^6	1.83×10^6	5.71×10^6	1.42×10^7	1.18×10^7	5.97	2.58	2.56×10^{-2}	1.00	1.44	up	Confident compound

ID	Description	Formula	RT (min)	m/z	Model1	Model2	Model3	FCDPE1	FCDPE2	FCDPE3	FC	log2FC	P value	ROC	VIP	Up/Down	Annotation category
C708	Val-Phe-Leu	$C_{20}H_{31}N_3O_4$	4.08	377.23	1.76×10^{6}	8.80×10^{5}	1.76×10^{6}	3.48×10^{6}	2.99×10^{6}	2.67×10^{6}	2.08	1.05	1.38×10^{-2}	1.00	1.48	up	Confident compound
C709	Ala-Ala-Ile	$C_{12}H_{23}N_3O_4$	1.96	273.17	8.26×10^{5}	3.41×10^{5}	5.90×10^{5}	6.76×10^{6}	1.61×10^{7}	1.46×10^{7}	21.34	4.42	1.48×10^{-2}	1.00	1.49	up	Confident compound
C746	PTI-1	$C_{21}H_{29}N_3S$	4.89	355.21	1.10×10^{6}	5.29×10^{5}	1.62×10^{6}	3.43×10^{6}	9.04×10^{6}	5.95×10^{6}	5.66	2.50	3.78×10^{-2}	1.00	1.40	up	Confident compound
C756	Leu-Gln-Leu	$C_{17}H_{32}N_4O_5$	3.24	372.24	1.69×10^{6}	7.72×10^{5}	9.10×10^{5}	6.83×10^{6}	1.05×10^{7}	9.94×10^{6}	8.08	3.01	2.44×10^{-3}	1.00	1.58	up	Confident compound
C761	2-{2-oxo-2-[(2-oxo-3-azepanyl)amino]ethoxy}acetic acid	$C_{10}H_{16}N_2O_5$	0.58	244.11	5.87×10^{6}	3.18×10^{6}	2.28×10^{6}	8.06×10^{6}	2.04×10^{7}	1.91×10^{7}	4.20	2.07	4.10×10^{-2}	1.00	1.38	up	Suspected contaminant
C762	(E)-3-(4-acetyloxy-2,3-dihydroxy-2,5,5,8a-tetramethyl-3,4,4a,6,7,8-hexahydro-1H-naphthalen-1-yl)prop-2-enoic acid	$C_{19}H_{30}O_6$	4.89	354.20	1.14×10^{6}	7.85×10^{5}	2.05×10^{6}	3.60×10^{6}	9.90×10^{6}	6.22×10^{6}	4.97	2.31	4.82×10^{-2}	1.00	1.36	up	Confident compound
C767	YLK	$C_{21}H_{34}N_4O_5$	4.39	422.25	3.37×10^{5}	1.75×10^{5}	2.68×10^{5}	3.54×10^{6}	4.47×10^{6}	1.46×10^{6}	12.13	3.60	3.12×10^{-2}	1.00	1.41	up	Confident compound
C773	Indolelactic acid	$C_{11}H_{11}NO_3$	2.83	205.07	4.17×10^{6}	3.13×10^{6}	3.70×10^{6}	5.51×10^{6}	7.58×10^{6}	6.68×10^{6}	1.80	0.85	1.20×10^{-2}	1.00	1.50	up	Confident compound
C781	MPK	$C_{16}H_{30}N_4O_4S$	3.58	374.20	2.26×10^{6}	7.48×10^{5}	8.62×10^{5}	7.65×10^{6}	4.35×10^{6}	4.05×10^{6}	4.14	2.05	3.14×10^{-2}	1.00	1.38	up	Confident compound
C786	MG(0:0/22:5(4Z,7Z,10Z,13Z,16Z)/0:0)	$C_{25}H_{40}O_4$	7.87	404.29	1.69×10^{5}	2.65×10^{5}	1.78×10^{6}	3.88×10^{6}	5.24×10^{6}	5.34×10^{6}	6.54	2.71	4.36×10^{-3}	1.00	1.57	up	Confident compound
C789	ethyl 1-(1,3-dimethyl-2,6-dioxo-1,2,3,6-tetrahydropyrimidin-4-yl)piperidine-4-carboxylate	$C_{14}H_{21}N_3O_4$	4.01	295.15	2.73×10^{6}	1.56×10^{6}	4.26×10^{6}	7.29×10^{6}	1.35×10^{7}	8.88×10^{6}	3.47	1.80	2.55×10^{-2}	1.00	1.45	up	Suspected contaminant
C810	Hexazinone	$C_{12}H_{20}N_4O_2$	1.01	252.16	3.79×10^{5}	2.56×10^{5}	2.42×10^{5}	1.01×10^{6}	2.51×10^{6}	1.59×10^{6}	5.84	2.55	3.26×10^{-2}	1.00	1.41	up	Suspected contaminant
C814	PC O-20:4	$C_{28}H_{50}NO_7P$	6.78	543.33	2.37×10^{6}	1.49×10^{6}	1.53×10^{6}	4.68×10^{6}	3.80×10^{6}	2.93×10^{6}	2.11	1.08	2.62×10^{-2}	1.00	1.41	up	Confident compound
C824	6-{[(6-Methoxy-2-morpholinopyridin-3-yl)amino]carbonyl}-3,4-dimethylcyclohex-3-ene-1-carboxylic acid	$C_{20}H_{27}N_3O_5$	4.14	389.19	7.49×10^{5}	6.31×10^{5}	1.11×10^{6}	4.29×10^{6}	1.19×10^{7}	1.37×10^{7}	12.01	3.59	3.43×10^{-2}	1.00	1.40	up	Suspected contaminant
C835	Pro-Glu-Arg	$C_{16}H_{28}N_6O_6$	3.75	400.21	1.40×10^{6}	4.49×10^{5}	1.06×10^{6}	3.95×10^{6}	9.00×10^{6}	1.35×10^{7}	9.10	3.19	4.73×10^{-2}	1.00	1.35	up	Confident compound
C878	Glu-Ala-Arg	$C_{14}H_{26}N_6O_6$	4.06	374.20	1.89×10^{6}	4.49×10^{5}	2.06×10^{6}	6.33×10^{6}	8.75×10^{6}	7.37×10^{6}	5.10	2.35	2.27×10^{-3}	1.00	1.59	up	Confident compound
C896	Irganox 565 degradation product C19H34O1N4S2	$C_{19}H_{34}O_1N_4S_2$	3.97	398.22	9.87×10^{5}	4.61×10^{5}	2.40×10^{6}	4.22×10^{6}	7.05×10^{6}	5.52×10^{6}	4.37	2.13	1.26×10^{-2}	1.00	1.51	up	Suspected contaminant
C905	Octaethylene glycol	$C_{16}H_{34}O_9$	3.10	370.22	4.02×10^{6}	1.03×10^{6}	1.40×10^{6}	6.06×10^{6}	7.57×10^{6}	7.45×10^{6}	3.27	1.71	9.98×10^{-3}	1.00	1.50	up	Suspected contaminant
C916	Val-Ile-Gln	$C_{16}H_{30}N_4O_5$	3.30	358.22	1.33×10^{6}	3.53×10^{5}	1.23×10^{6}	4.91×10^{6}	8.41×10^{6}	6.85×10^{6}	6.92	2.79	5.54×10^{-3}	1.00	1.55	up	Confident compound
C935	EPK	$C_{16}H_{28}N_4O_6$	3.23	372.20	1.56×10^{6}	1.40×10^{5}	1.17×10^{6}	6.57×10^{6}	8.04×10^{6}	5.76×10^{6}	7.10	2.83	1.79×10^{-3}	1.00	1.59	up	Confident compound
C970	1,4-Anhydro-2,5-dideoxy-5-[(isopropylcarbamoyl)amino]-2-(3-oxetanylamino)-D-arabinitol	$C_{12}H_{23}N_3O_4$	2.31	273.17	1.23×10^{6}	2.81×10^{5}	5.84×10^{5}	4.61×10^{6}	9.69×10^{6}	8.02×10^{6}	10.64	3.41	1.14×10^{-2}	1.00	1.51	up	Suspected contaminant
C990	HET0016	$C_{12}H_{18}N_2O$	1.32	206.14	2.11×10^{5}	2.20×10^{5}	3.58×10^{5}	3.38×10^{6}	6.80×10^{6}	7.21×10^{6}	22.05	4.46	1.04×10^{-2}	1.00	1.52	up	Suspected contaminant
C998	Leu-Gly-Leu	$C_{14}H_{27}N_3O_4$	3.71	301.20	1.80×10^{6}	1.20×10^{6}	1.86×10^{6}	2.64×10^{6}	2.56×10^{6}	2.69×10^{6}	1.62	0.70	9.39×10^{-3}	1.00	1.51	up	Confident compound
C1002	Sphingosine	$C_{18}H_{37}NO_2$	6.14	299.28	3.37×10^{6}	2.45×10^{6}	2.08×10^{6}	3.44×10^{6}	4.71×10^{6}	5.07×10^{6}	1.68	0.75	4.65×10^{-2}	1.00	1.34	up	Confident compound
C1037	PZMGZXATJGSIDQ-ZRDIBKRKSA-N	$C_{21}H_{38}O_5$	7.20	370.27	1.83×10^{6}	2.29×10^{6}	1.13×10^{6}	3.25×10^{6}	2.49×10^{6}	3.14×10^{6}	1.69	0.76	4.24×10^{-2}	1.00	1.34	up	Confident compound

ID	Description	Formula	RT (min)	m/z	Model1	Model2	Model3	FCDPE1	FCDPE2	FCDPE3	FC	log2FC	P value	ROC	VIP	Up/Down	Annotation category
C1049	N-Acetyl-L-methionine	$C_7H_{13}NO_3S$	0.60	191.06	1.61×10^{7}	5.14×10^{6}	6.65×10^{6}	2.07×10^{7}	3.76×10^{7}	5.29×10^{7}	3.98	1.99	4.87×10^{-2}	1.00	1.34	up	Confident compound
C1053	2-Ethoxyethyl acrylate	$C_7H_{12}O_3$	7.99	144.08	1.69×10^{6}	5.11×10^{5}	3.88×10^{5}	1.68×10^{6}	3.22×10^{6}	3.10×10^{6}	3.09	1.63	4.93×10^{-2}	0.89	1.34	up	Suspected contaminant
C1059	MQENNBZSORVHQZ-XPNMZNARSA-N	$C_{19}H_{34}O_8$	4.07	390.23	1.13×10^{6}	4.50×10^{5}	1.72×10^{6}	5.12×10^{6}	7.16×10^{6}	3.10×10^{6}	4.67	2.22	3.05×10^{-2}	1.00	1.42	up	Confident compound
C1065	tween X8	$C_{36}H_{70}O_{14}$	9.49	726.48	7.67×10^{5}	2.15×10^{5}	1.96×10^{5}	1.17×10^{6}	1.41×10^{6}	1.20×10^{6}	3.21	1.68	1.26×10^{-2}	1.00	1.48	up	Suspected contaminant
C1080	2-[(2R,4aR,8R,8aR)-8-hydroxy-4a,8-dimethyl-decahydronaphthalen-2-yl]prop-2-enoic acid	$C_{15}H_{24}O_3$	3.88	252.17	5.72×10^{5}	7.30×10^{5}	1.58×10^{6}	3.33×10^{6}	4.40×10^{6}	2.36×10^{6}	3.50	1.81	2.27×10^{-2}	1.00	1.46	up	Confident compound
C1101	Piperanine	$C_{17}H_{21}NO_3$	2.84	287.15	2.14×10^{6}	6.65×10^{5}	1.45×10^{6}	3.14×10^{6}	7.63×10^{6}	6.40×10^{6}	4.04	2.01	3.76×10^{-2}	1.00	1.39	up	Confident compound
C1104	Tributyl trimellitate	$C_{21}H_{30}O_6$	4.83	378.20	2.90×10^{5}	4.55×10^{5}	4.54×10^{5}	1.86×10^{6}	5.30×10^{6}	5.26×10^{6}	10.36	3.37	3.07×10^{-2}	1.00	1.42	up	Suspected contaminant
C1111	Leu-Gly-Gln	$C_{13}H_{24}N_4O_5$	1.25	316.17	5.60×10^{5}	3.95×10^{5}	4.02×10^{5}	1.63×10^{6}	3.79×10^{6}	3.82×10^{6}	6.81	2.77	2.24×10^{-2}	1.00	1.45	up	Confident compound
C1124	A-796260 Degradant	$C_{22}H_{30}N_2O_2$	3.26	354.23	7.78×10^{5}	4.82×10^{5}	9.41×10^{5}	3.59×10^{6}	6.06×10^{6}	5.37×10^{6}	6.83	2.77	4.63×10^{-3}	1.00	1.56	up	Suspected contaminant
C1132	3,3-Dimethyl-1,5-dioxacyclopentadecane-6,15-dione	$C_{15}H_{26}O_4$	4.54	270.18	1.37×10^{6}	1.12×10^{6}	1.19×10^{6}	1.94×10^{6}	1.96×10^{6}	1.63×10^{6}	1.50	0.59	9.82×10^{-3}	1.00	1.51	up	Suspected contaminant
C1138	1,4-Anhydro-5-[(cyclopropylcarbonyl)amino]-2,5-dideoxy-2-(4-piperidinylamino)-D-arabinitol	$C_{14}H_{25}N_3O_3$	3.44	283.19	2.87×10^{6}	6.11×10^{5}	6.30×10^{5}	3.36×10^{6}	4.25×10^{6}	4.52×10^{6}	2.95	1.56	3.21×10^{-2}	1.00	1.39	up	Suspected contaminant
C1166	1-{[(2R,3S,4R,5S)-3,4-Dihydroxy-5-{2-[(2R)-2-(methoxymethyl)-1-pyrrolidinyl]-2-oxoethyl}tetrahydro-2-furanyl]methyl}-3-isopropylurea	$C_{17}H_{31}N_3O_6$	3.61	373.22	4.07×10^{5}	1.57×10^{5}	4.29×10^{5}	2.04×10^{6}	3.15×10^{6}	2.47×10^{6}	7.71	2.95	2.70×10^{-3}	1.00	1.58	up	Suspected contaminant
C1229	Capsaicin	$C_{18}H_{27}NO_3$	2.77	305.20	6.12×10^{5}	3.41×10^{5}	5.65×10^{5}	1.13×10^{6}	3.03×10^{6}	2.72×10^{6}	4.53	2.18	3.98×10^{-2}	1.00	1.39	up	Confident compound
C1237	5,7-dihydroxy-3-(4-hydroxyphenyl)-4H-chromen-4-one	$C_{15}H_{10}O_5$	3.85	270.05	2.13×10^{5}	1.90×10^{5}	2.12×10^{5}	2.44×10^{5}	3.68×10^{5}	3.57×10^{5}	1.58	0.66	4.29×10^{-2}	1.00	1.37	up	Confident compound
C1246	Palmitamide	$C_{16}H_{33}NO$	6.25	255.26	1.87×10^{6}	1.11×10^{6}	1.51×10^{6}	2.42×10^{6}	2.49×10^{6}	3.01×10^{6}	1.76	0.82	1.66×10^{-2}	1.00	1.46	up	Confident compound
C1255	N-{[(2R,4S,5S)-5-(1-Piperidinylmethyl)-1-azabicyclo[2.2.2]oct-2-yl]methyl}-4-(trifluoromethyl)benzamide	$C_{22}H_{30}F_3N_3O$	3.51	409.23	2.96×10^{5}	2.41×10^{5}	1.75×10^{5}	1.74×10^{6}	2.53×10^{6}	2.20×10^{6}	9.09	3.18	1.16×10^{-3}	1.00	1.61	up	Suspected contaminant
C1266	Arg-Ser-Asn	$C_{13}H_{25}N_7O_6$	3.81	375.18	3.37×10^{5}	9.58×10^{5}	1.14×10^{6}	2.34×10^{6}	5.55×10^{6}	4.58×10^{6}	5.13	2.36	2.70×10^{-2}	1.00	1.44	up	Confident compound
C1334	Testosterone 17-phenylpropionate	$C_{28}H_{36}O_3$	7.97	420.26	3.86×10^{5}	3.12×10^{5}	3.06×10^{5}	2.81×10^{6}	3.34×10^{6}	5.44×10^{6}	11.54	3.53	1.18×10^{-2}	1.00	1.49	up	Suspected contaminant
C1337	N,N-Bis(2-hydroxyethyl)dodecanamide	$C_{16}H_{33}NO_3$	6.93	287.25	2.18×10^{6}	1.43×10^{6}	1.63×10^{6}	2.64×10^{6}	2.47×10^{6}	2.81×10^{6}	1.51	0.59	2.21×10^{-2}	1.00	1.42	up	Suspected contaminant
C1370	Glutamyltryptophan	$C_{16}H_{19}N_3O_5$	3.47	333.13	7.19×10^{5}	4.22×10^{5}	1.41×10^{6}	1.94×10^{6}	2.80×10^{6}	2.27×10^{6}	2.75	1.46	1.81×10^{-2}	1.00	1.48	up	Confident compound
C1388	JWH 073 6-methoxyindole analog	$C_{24}H_{23}NO_2$	5.32	357.17	8.06×10^{4}	6.42×10^{4}	7.88×10^{4}	1.77×10^{6}	1.59×10^{6}	1.14×10^{6}	20.09	4.33	1.60×10^{-3}	1.00	1.59	up	Suspected contaminant
C1389	ADBICA	$C_{20}H_{29}N_3O_2$	3.24	343.23	1.00×10^{6}	1.23×10^{5}	2.60×10^{5}	1.21×10^{6}	3.25×10^{6}	3.24×10^{6}	5.56	2.48	4.48×10^{-2}	1.00	1.36	up	Suspected contaminant
C1390	N,N-bis(1,4-Dimethylpentyl)-p-phenylenediamine	$C_{20}H_{36}N_2$	7.92	304.29	6.22×10^{5}	5.58×10^{5}	5.57×10^{5}	8.07×10^{5}	1.60×10^{6}	1.53×10^{6}	2.26	1.18	4.47×10^{-2}	1.00	1.37	up	Suspected contaminant
C1419	octhilinone [ANSI]	$C_{11}H_{19}NOS$	3.03	213.12	8.83×10^{5}	2.66×10^{5}	4.52×10^{5}	1.39×10^{6}	2.75×10^{6}	2.02×10^{6}	3.85	1.94	2.48×10^{-2}	1.00	1.44	up	Suspected contaminant
C1422	2-(1-Adamantyl)-6-{[(5-hydroxy-4-pentyl-4H-1,2,4-triazol-3-yl)thio]methyl}pyrimidin-4-ol	$C_{22}H_{31}N_5O_2S$	3.08	429.22	7.78×10^{5}	8.50×10^{4}	3.32×10^{5}	2.59×10^{6}	4.18×10^{6}	3.19×10^{6}	8.34	3.06	4.48×10^{-3}	1.00	1.56	up	Suspected contaminant

ID	Description	Formula	RT (min)	m/z	Model1	Model2	Model3	FCDPE1	FCDPE2	FCDPE3	FC	log2FC	P value	ROC	VIP	Up/Down	Annotation category
C1451	Tiglylcarnitine	$C_{12}H_{21}NO_4$	3.27	243.15	7.09×10^{4}	7.17×10^{4}	7.51×10^{4}	1.42×10^{6}	3.91×10^{6}	4.23×10^{6}	43.92	5.46	2.46×10^{-2}	1.00	1.44	up	Confident compound
C1452	N-Acetylhistidine	$C_8H_{11}N_3O_3$	0.54	197.08	9.40×10^{6}	5.05×10^{6}	8.11×10^{6}	1.21×10^{7}	1.03×10^{7}	1.25×10^{7}	1.54	0.63	4.90×10^{-2}	1.00	1.32	up	Confident compound
C1476	Ethoxylated trimethylolpropane triacrylate	$C_{21}H_{32}O_9$	4.14	428.21	3.57×10^{5}	9.85×10^{4}	1.76×10^{5}	8.82×10^{5}	1.64×10^{6}	1.24×10^{6}	5.95	2.57	1.09×10^{-2}	1.00	1.51	up	Suspected contaminant
C1479	Dodecyltrimethylammonium	$C_{15}H_{33}N$	6.37	227.26	7.84×10^{5}	7.36×10^{5}	5.37×10^{5}	2.34×10^{6}	1.43×10^{6}	1.70×10^{6}	2.66	1.41	1.52×10^{-2}	1.00	1.46	up	Suspected contaminant
C1490	Estrone	$C_{18}H_{22}O_2$	4.35	270.16	1.11×10^{6}	5.76×10^{5}	9.97×10^{5}	7.49×10^{6}	1.12×10^{7}	7.45×10^{6}	9.75	3.29	3.39×10^{-3}	1.00	1.58	up	Confident compound
C1496	Imidazolelactic acid	$C_6H_8N_2O_3$	0.54	156.05	4.32×10^{6}	3.16×10^{6}	4.13×10^{6}	7.61×10^{6}	8.66×10^{6}	1.09×10^{7}	2.33	1.22	7.18×10^{-3}	1.00	1.53	up	Confident compound
C1502	tween X15	$C_{44}H_{86}O_{21}$	3.83	950.56	9.57×10^{4}	8.53×10^{4}	9.52×10^{4}	8.15×10^{5}	2.19×10^{6}	2.19×10^{6}	18.81	4.23	2.33×10^{-2}	1.00	1.45	up	Suspected contaminant
C1505	3-(propan-2-yl)-octahydropyrrolo[1,2-a]pyrazine-1,4-dione	$C_{10}H_{16}N_2O_2$	2.61	196.12	7.59×10^{5}	1.02×10^{6}	9.66×10^{5}	1.45×10^{6}	2.19×10^{6}	1.74×10^{6}	1.96	0.97	1.86×10^{-2}	1.00	1.48	up	Confident compound
C1510	3,6,9,12,15,18-Hexaoxaicosane-1,20-diol	$C_{14}H_{30}O_8$	3.30	326.20	4.96×10^{5}	4.42×10^{5}	3.74×10^{5}	2.18×10^{6}	1.63×10^{6}	1.55×10^{6}	4.08	2.03	2.53×10^{-3}	1.00	1.57	up	Suspected contaminant
C1514	4-(1-Phenylethyl)-N-[4-(1-phenylethyl)phenyl]benzenamine	$C_{28}H_{27}N$	4.00	377.22	3.84×10^{5}	9.02×10^{4}	3.65×10^{5}	1.61×10^{6}	2.22×10^{6}	1.60×10^{6}	6.47	2.69	2.48×10^{-3}	1.00	1.59	up	Suspected contaminant
C1524	UJRMJTIXXKZFGB-KWEBYEANSA-N	$C_{32}H_{52}O_5$	7.84	516.38	6.61×10^{5}	9.66×10^{5}	7.79×10^{5}	1.86×10^{6}	2.91×10^{6}	3.70×10^{6}	3.52	1.82	2.00×10^{-2}	1.00	1.46	up	Confident compound
C1541	Bis[2,4-bis(2-methyl-2-propanyl)phenyl] phosphonate	$C_{28}H_{43}O_3P$	3.71	458.29	2.36×10^{5}	1.81×10^{5}	3.20×10^{5}	1.23×10^{6}	1.71×10^{6}	1.56×10^{6}	6.09	2.61	1.06×10^{-3}	1.00	1.61	up	Suspected contaminant
C1572	Arg-Arg-Asn	$C_{16}H_{32}N_{10}O_5$	3.72	444.26	1.56×10^{5}	1.20×10^{5}	2.97×10^{5}	8.39×10^{5}	1.50×10^{6}	1.28×10^{6}	6.31	2.66	7.32×10^{-3}	1.00	1.54	up	Confident compound
C1634	PS(14:0/20:3(8Z,11Z,14Z))	$C_{40}H_{72}NO_{10}P$	3.91	757.49	3.70×10^{5}	4.41×10^{5}	2.85×10^{5}	7.49×10^{5}	1.04×10^{6}	1.51×10^{6}	3.01	1.59	3.12×10^{-2}	1.00	1.40	up	Confident compound
C1637	3-Hydroxy-5-(hydroxymethyl)-2-methylisonicotinaldehyde oxime	$C_8H_{10}N_2O_3$	10.29	182.07	1.32×10^{6}	6.62×10^{5}	5.44×10^{5}	1.68×10^{6}	1.67×10^{6}	1.70×10^{6}	2.00	1.00	2.51×10^{-2}	1.00	1.41	up	Confident compound
C1640	DUNFAZHEVVYXGG-XTYQBWKXSA-N	$C_{25}H_{30}O_{12}$	3.86	522.17	1.40×10^{5}	1.24×10^{5}	1.39×10^{5}	1.60×10^{5}	2.42×10^{5}	2.34×10^{5}	1.58	0.66	4.29×10^{-2}	1.00	1.37	up	Confident compound
C1642	JWH-307	$C_{26}H_{24}FNO$	4.15	385.19	5.16×10^{5}	1.37×10^{5}	1.82×10^{5}	8.14×10^{5}	1.75×10^{6}	1.45×10^{6}	4.81	2.26	2.43×10^{-2}	1.00	1.44	up	Suspected contaminant
C1643	Methoxyacetyl fentanyl-d5	$C_{22}H_{23}D_5N_2O_2$	3.90	357.25	3.61×10^{5}	6.01×10^{4}	4.32×10^{5}	1.65×10^{6}	2.24×10^{6}	1.85×10^{6}	6.74	2.75	1.42×10^{-3}	1.00	1.60	up	Suspected contaminant
C1645	2,5,8,11,14,17-Hexaoxanonadecan-19-ol	$C_{13}H_{28}O_7$	3.17	296.18	2.61×10^{5}	2.51×10^{5}	3.06×10^{5}	1.23×10^{6}	2.12×10^{6}	2.46×10^{6}	7.09	2.83	1.05×10^{-2}	1.00	1.51	up	Suspected contaminant
C1653	DNK	$C_{14}H_{25}N_5O_7$	3.41	375.18	3.59×10^{5}	1.76×10^{5}	1.47×10^{5}	7.21×10^{5}	5.08×10^{5}	6.33×10^{5}	2.73	1.45	1.23×10^{-2}	1.00	1.47	up	Confident compound
C1658	2-{[4-(2,2-Dicyanovinyl)-3-methylphenyl](ethyl)amino}ethyl phenylcarbamate	$C_{22}H_{22}N_4O_2$	4.70	374.17	1.89×10^{5}	1.64×10^{5}	3.78×10^{5}	9.80×10^{5}	2.49×10^{6}	1.71×10^{6}	7.08	2.82	2.83×10^{-2}	1.00	1.43	up	Suspected contaminant
C1689	SDB-006 N-phenyl analog	$C_{20}H_{22}N_2O$	3.28	306.18	6.71×10^{5}	2.19×10^{5}	4.69×10^{5}	7.95×10^{5}	9.52×10^{5}	1.17×10^{6}	2.15	1.10	3.82×10^{-2}	1.00	1.37	up	Suspected contaminant
C1701	Dioctylamine	$C_{16}H_{35}N$	7.10	241.28	5.63×10^{5}	4.21×10^{5}	4.51×10^{5}	1.42×10^{6}	8.28×10^{5}	9.66×10^{5}	2.24	1.16	3.23×10^{-2}	1.00	1.38	up	Suspected contaminant
C1711	Ile-Val-Leu	$C_{17}H_{33}N_3O_4$	4.26	343.25	2.06×10^{5}	1.09×10^{5}	1.33×10^{5}	2.93×10^{5}	6.04×10^{5}	3.95×10^{5}	2.89	1.53	4.30×10^{-2}	1.00	1.37	up	Confident compound
C1714	Methionyl-Phenylalanine	$C_{14}H_{20}N_2O_3S$	3.44	296.12	2.49×10^{5}	1.24×10^{5}	1.92×10^{5}	4.90×10^{5}	7.44×10^{5}	4.89×10^{5}	3.05	1.61	1.39×10^{-2}	1.00	1.49	up	Confident compound
C1715	1,2,2,6,6-Pentamethyl-4-piperidinyl acrylate	$C_{13}H_{23}NO_2$	4.50	225.17	5.90×10^{5}	6.82×10^{5}	4.53×10^{5}	1.06×10^{6}	7.65×10^{5}	8.39×10^{5}	1.55	0.63	4.84×10^{-2}	1.00	1.32	up	Suspected contaminant

ID	Description	Formula	RT (min)	m/z	Model1	Model2	Model3	FCDPE1	FCDPE2	FCDPE3	FC	log2FC	P value	ROC	VIP	Up/Down	Annotation category
C1730	1-(2-Pyridinyl)-2-butanone	$C_9H_{11}NO$	3.73	149.08	4.47×10^5	2.33×10^5	3.02×10^5	5.87×10^5	4.79×10^5	6.48×10^5	1.74	0.80	3.82×10^{-2}	1.00	1.35	up	Confident compound
C1731	Val-Arg-Trp	$C_{22}H_{33}N_7O_4$	3.40	459.26	7.53×10^4	6.44×10^4	6.69×10^4	2.09×10^5	4.82×10^5	5.34×10^5	5.93	2.57	2.83×10^{-2}	1.00	1.42	up	Confident compound
C1733	gamma-Glutamylphenylalanine	$C_{14}H_{18}N_2O_5$	3.45	294.12	2.97×10^5	9.40×10^4	2.41×10^5	1.12×10^6	1.41×10^6	1.16×10^6	5.82	2.54	7.41×10^{-4}	1.00	1.61	up	Confident compound
C1740	N-{4-[(2R,3R)-3-(Hydroxymethyl)-4-methyl-5-oxo-2-morpholinyl]phenyl}cyclopentanecarboxamide	$C_{18}H_{24}N_2O_4$	5.43	332.17	1.24×10^5	8.80×10^4	3.50×10^5	1.23×10^6	1.05×10^6	5.12×10^5	4.97	2.31	3.23×10^{-2}	1.00	1.40	up	Suspected contaminant
C1744	Triphenyl phosphate	$C_{18}H_{15}O_4P$	7.00	326.07	5.60×10^4	1.45×10^5	5.30×10^4	1.69×10^5	2.39×10^5	2.03×10^5	2.40	1.26	3.11×10^{-2}	1.00	1.41	up	Suspected contaminant
C1756	Panthenol	$C_9H_{19}NO_4$	1.63	205.13	2.62×10^5	2.54×10^5	3.32×10^5	1.42×10^6	1.14×10^6	1.34×10^6	4.59	2.20	3.06×10^{-4}	1.00	1.62	up	Confident compound
C1794	gamma-Glutamyltyrosine	$C_{14}H_{18}N_2O_6$	1.46	310.12	3.82×10^5	6.58×10^4	5.02×10^5	4.67×10^6	4.01×10^6	2.90×10^6	12.19	3.61	2.66×10^{-3}	1.00	1.57	up	Confident compound
C1820	3-Methacryloxypropylmethyldiethoxysilane	$C_{12}H_{24}O_4Si$	3.73	260.15	5.46×10^5	1.75×10^5	1.09×10^5	6.83×10^5	9.90×10^5	1.13×10^6	3.38	1.76	2.55×10^{-2}	1.00	1.42	up	Suspected contaminant
C1823	1-[(1S,2S,3S,4R,5R)-4-(4-Benzyl-1-piperidinyl)-3-hydroxy-6,8-dioxabicyclo[3.2.1]oct-2-yl]-3-ethylurea	$C_{21}H_{31}N_3O_4$	4.48	389.23	8.49×10^4	3.40×10^5	1.40×10^5	5.86×10^5	5.09×10^5	4.52×10^5	2.73	1.45	1.96×10^{-2}	1.00	1.45	up	Suspected contaminant
C1845	L-Acetylcarnitine	$C_9H_{18}NO_4$	3.20	204.12	1.46×10^5	1.40×10^5	1.71×10^5	2.22×10^5	2.73×10^5	2.40×10^5	1.61	0.69	6.28×10^{-3}	1.00	1.55	up	Confident compound
C1860	4-Hydroxynonenal	$C_9H_{16}O_2$	4.44	156.12	5.84×10^5	9.81×10^5	1.08×10^6	1.45×10^6	1.38×10^6	1.24×10^6	1.54	0.62	4.37×10^{-2}	1.00	1.37	up	Confident compound
C1903	(2R,3R,4S,5S,6R)-2-{[(2E)-4-ethenyl-2,5-dimethylhexa-2,5-dien-1-yl]oxy}-6-(hydroxymethyl)oxane-3,4,5-triol	$C_{16}H_{26}O_6$	6.51	314.17	3.05×10^5	2.18×10^5	2.18×10^5	6.66×10^5	5.92×10^5	3.66×10^5	2.19	1.13	3.58×10^{-2}	1.00	1.38	up	Confident compound
C1919	Nervonyl carnitine	$C_{31}H_{60}NO_4$	8.37	510.45	4.07×10^5	1.91×10^5	2.16×10^5	5.32×10^5	8.40×10^5	8.95×10^5	2.78	1.48	2.14×10^{-2}	1.00	1.45	up	Confident compound
C1973	Isoleucyl-Serine	$C_9H_{18}N_2O_4$	0.90	218.13	3.08×10^6	5.73×10^5	1.22×10^6	5.88×10^6	8.94×10^6	7.70×10^6	4.62	2.21	7.21×10^{-3}	1.00	1.53	up	Confident compound
C1977	Arg-Met-His	$C_{17}H_{30}N_8O_4S$	3.50	442.21	1.06×10^5	1.02×10^5	8.82×10^4	2.28×10^5	4.86×10^5	5.12×10^5	4.13	2.05	2.69×10^{-2}	1.00	1.43	up	Confident compound
C1993	β-Hydroxyfentanyl	$C_{22}H_{28}N_2O_2$	4.07	352.21	2.19×10^5	7.74×10^4	9.74×10^4	3.33×10^5	2.46×10^5	4.23×10^5	2.54	1.35	3.99×10^{-2}	1.00	1.35	up	Suspected contaminant
C2024	LPG O-18:2	$C_{24}H_{47}O_8P$	7.37	494.30	1.71×10^8	7.35×10^7	5.57×10^7	1.95×10^8	2.53×10^8	1.95×10^8	2.14	1.10	4.78×10^{-2}	1.00	1.33	up	Confident compound
C2053	2-(Acetylamino)hexanoic acid	$C_8H_{15}NO_3$	3.66	173.11	5.02×10^7	1.26×10^7	1.86×10^7	1.44×10^8	7.56×10^7	8.91×10^7	3.79	1.92	3.42×10^{-2}	1.00	1.37	up	Confident compound
C2066	N-Acetyl-DL-norvaline	$C_7H_{13}NO_3$	0.61	159.09	3.14×10^7	1.82×10^7	4.11×10^7	7.89×10^7	4.63×10^7	6.84×10^7	2.13	1.09	4.25×10^{-2}	1.00	1.34	up	Confident compound
C2081	4-Hydroxybenzaldehyde	$C_7H_6O_2$	3.47	122.04	5.16×10^7	6.75×10^7	4.32×10^7	1.23×10^8	9.20×10^7	1.45×10^8	2.22	1.15	1.79×10^{-2}	1.00	1.45	up	Confident compound
C2082	2-methoxyhydroquinone	$C_7H_8O_3$	3.47	140.05	5.16×10^7	6.75×10^7	4.32×10^7	1.23×10^8	1.52×10^8	1.45×10^8	2.59	1.37	1.64×10^{-3}	1.00	1.59	up	Confident compound
C2083	LPG O-18:3	$C_{24}H_{45}O_8P$	6.90	492.29	2.94×10^7	1.51×10^7	1.71×10^7	3.88×10^7	5.38×10^7	4.30×10^7	2.20	1.14	1.77×10^{-2}	1.00	1.46	up	Confident compound
C2109	2-(acetylamino)-4-(methylthio)butanoic acid	$C_7H_{13}NO_3S$	2.79	191.06	1.42×10^7	3.48×10^6	5.86×10^6	4.01×10^7	2.46×10^7	3.32×10^7	4.15	2.05	1.11×10^{-2}	1.00	1.48	up	Confident compound
C2139	nylon oligomers C4	$C_{24}H_{42}N_4O_4$	7.28	450.32	7.95×10^6	3.08×10^6	3.62×10^6	1.43×10^7	2.48×10^7	3.25×10^7	4.89	2.29	2.58×10^{-2}	1.00	1.43	up	Suspected contaminant
C2149	2-[(2S,3R,4S,5R)-5-(Acetamidomethyl)-3,4-dihydroxytetrahydro-2-furanyl]-N-isopropylacetamide	$C_{12}H_{22}N_2O_5$	3.10	274.15	9.23×10^6	2.98×10^6	8.10×10^6	3.66×10^7	7.94×10^7	6.76×10^7	9.04	3.18	1.35×10^{-2}	1.00	1.50	up	Suspected contaminant

ID	Description	Formula	RT (min)	m/z	Model1	Model2	Model3	FCDPE1	FCDPE2	FCDPE3	FC	log2FC	P value	ROC	VIP	Up/Down	Annotation category
C2150	N-α-Acetyl-L-asparagine	$C_6H_{10}N_2O_4$	0.54	174.06	3.01×10^{7}	6.19×10^{6}	1.14×10^{7}	5.36×10^{7}	3.01×10^{7}	5.61×10^{7}	2.93	1.55	4.92×10^{-2}	1.00	1.31	up	Confident compound
C2166	4-Dodecylbenzenesulfonic acid	$C_{18}H_{30}O_3S$	6.99	326.19	3.17×10^{7}	2.34×10^{7}	2.20×10^{7}	5.00×10^{7}	4.04×10^{7}	4.77×10^{7}	1.79	0.84	8.25×10^{-3}	1.00	1.50	up	Suspected contaminant
C2216	Dodecyl sulfate	$C_{12}H_{26}O_4S$	6.38	266.16	2.00×10^{7}	1.53×10^{7}	1.48×10^{7}	3.27×10^{7}	2.76×10^{7}	2.55×10^{7}	1.71	0.78	1.17×10^{-2}	1.00	1.48	up	Suspected contaminant
C2219	Monobutyl phthalate	$C_{12}H_{14}O_4$	5.47	222.09	3.15×10^{5}	3.35×10^{5}	2.61×10^{5}	4.14×10^{5}	5.01×10^{5}	4.77×10^{5}	1.53	0.61	9.37×10^{-3}	1.00	1.52	up	Suspected contaminant
C2224	N-Acetylvaline	$C_7H_{13}NO_3$	0.83	159.09	1.20×10^{7}	6.03×10^{6}	1.05×10^{7}	1.83×10^{7}	1.69×10^{7}	1.51×10^{7}	1.76	0.82	2.26×10^{-2}	1.00	1.43	up	Confident compound
C2237	PG 16:0_18:2	$C_{40}H_{75}O_{10}P$	10.09	746.51	6.61×10^{6}	8.80×10^{6}	6.47×10^{6}	1.38×10^{7}	1.37×10^{7}	1.35×10^{7}	1.88	0.91	1.09×10^{-3}	1.00	1.60	up	Confident compound
C2253	PI(16:0/18:2(9Z,12Z))	$C_{43}H_{79}O_{13}P$	13.03	834.53	1.37×10^{7}	8.07×10^{6}	1.27×10^{7}	2.85×10^{7}	2.19×10^{7}	2.14×10^{7}	2.08	1.06	1.23×10^{-2}	1.00	1.48	up	Confident compound
C2260	C12-AE1S (TENTATIVE)	$C_{14}H_{30}O_5S$	6.66	310.18	1.85×10^{7}	1.66×10^{7}	1.37×10^{7}	2.78×10^{7}	2.61×10^{7}	2.97×10^{7}	1.71	0.78	2.71×10^{-3}	1.00	1.56	up	Suspected contaminant
C2284	6-Aminocaproic acid	$C_6H_{13}NO_2$	3.51	131.09	4.48×10^{6}	1.77×10^{6}	2.46×10^{6}	1.71×10^{7}	7.66×10^{6}	1.22×10^{7}	4.24	2.09	2.93×10^{-2}	1.00	1.39	up	Confident compound
C2287	Ile-Asp	$C_{10}H_{18}N_2O_5$	1.10	246.12	6.95×10^{6}	1.98×10^{6}	2.28×10^{6}	1.64×10^{7}	2.24×10^{7}	2.16×10^{7}	5.38	2.43	2.74×10^{-3}	1.00	1.57	up	Confident compound
C2301	4-Toluenesulfonamide	$C_7H_9NO_2S$	5.51	171.04	1.16×10^{7}	1.38×10^{7}	1.18×10^{7}	2.25×10^{7}	1.64×10^{7}	1.91×10^{7}	1.56	0.64	2.14×10^{-2}	1.00	1.43	up	Suspected contaminant
C2305	NP-006255	$C_{17}H_{26}O_4$	7.11	294.19	1.30×10^{7}	1.00×10^{7}	1.01×10^{7}	2.16×10^{7}	1.78×10^{7}	1.87×10^{7}	1.75	0.81	5.36×10^{-3}	1.00	1.53	up	Confident compound
C2337	gamma-Glutamylvaline	$C_{10}H_{18}N_2O_5$	1.39	246.12	5.59×10^{6}	1.80×10^{6}	1.82×10^{6}	1.44×10^{7}	2.27×10^{7}	2.10×10^{7}	6.31	2.66	4.53×10^{-3}	1.00	1.56	up	Confident compound
C2348	5-Aminovaleric acid	$C_5H_{11}NO_2$	0.60	117.08	2.17×10^{6}	1.48×10^{6}	2.01×10^{6}	7.19×10^{6}	3.94×10^{6}	6.42×10^{6}	3.10	1.63	1.67×10^{-2}	1.00	1.45	up	Confident compound
C2366	gamma-Glutamylisoleucine	$C_{11}H_{20}N_2O_5$	1.36	260.14	4.59×10^{6}	1.35×10^{6}	1.76×10^{6}	1.60×10^{7}	1.70×10^{7}	1.64×10^{7}	6.41	2.68	2.01×10^{-4}	1.00	1.62	up	Confident compound
C2385	1-methoxy-1-trimethylsilyloxy-2-methylpropene	$C_8H_{18}O_2Si$	3.45	174.11	1.23×10^{6}	9.18×10^{5}	1.04×10^{6}	4.30×10^{6}	4.55×10^{6}	3.68×10^{6}	3.93	1.97	3.43×10^{-4}	1.00	1.62	up	Suspected contaminant
C2401	Ostruthin	$C_{19}H_{22}O_3$	5.16	298.15	2.98×10^{6}	2.18×10^{5}	2.19×10^{6}	6.76×10^{6}	9.60×10^{6}	8.03×10^{6}	4.52	2.18	5.51×10^{-3}	1.00	1.55	up	Confident compound
C2405	Ile-Phe	$C_{15}H_{22}N_2O_3$	3.79	278.16	2.07×10^{6}	6.51×10^{5}	3.55×10^{6}	5.77×10^{6}	7.57×10^{6}	4.99×10^{6}	2.93	1.55	2.37×10^{-2}	1.00	1.45	up	Confident compound
C2407	UR-144 5-pentanoic acid metabolite	$C_{21}H_{27}NO_3$	4.58	341.19	7.35×10^{5}	3.02×10^{5}	9.45×10^{5}	4.60×10^{6}	1.08×10^{7}	7.81×10^{6}	11.71	3.55	1.71×10^{-2}	1.00	1.48	up	Suspected contaminant
C2422	Ala-Ile	$C_9H_{18}N_2O_3$	2.06	202.13	1.47×10^{6}	8.34×10^{5}	1.43×10^{6}	4.44×10^{6}	1.20×10^{7}	8.35×10^{6}	6.64	2.73	3.28×10^{-2}	1.00	1.41	up	Confident compound
C2430	Psoromic acid	$C_{18}H_{14}O_8$	0.69	358.06	1.20×10^{5}	1.27×10^{5}	1.40×10^{5}	1.24×10^{7}	3.79×10^{6}	1.26×10^{7}	74.19	6.21	3.11×10^{-2}	1.00	1.38	up	Confident compound
C2431	Butabarbital	$C_{10}H_{16}N_2O_3$	1.78	212.12	2.07×10^{6}	1.32×10^{6}	2.11×10^{6}	7.47×10^{6}	1.05×10^{7}	8.82×10^{6}	4.87	2.29	1.53×10^{-3}	1.00	1.60	up	Suspected contaminant
C2436	CSRXCVLPMJBQIM-UHFFFAOYSA-N	$C_{11}H_{18}N_2O_4$	1.42	242.13	2.28×10^{6}	1.09×10^{6}	1.14×10^{6}	1.21×10^{7}	1.44×10^{7}	1.35×10^{7}	8.89	3.15	1.03×10^{-4}	1.00	1.63	up	Confident compound
C2437	Ile-Gly	$C_8H_{16}N_2O_3$	0.94	188.12	3.27×10^{6}	1.09×10^{6}	1.32×10^{6}	6.17×10^{6}	1.56×10^{7}	1.17×10^{7}	5.89	2.56	3.07×10^{-2}	1.00	1.42	up	Confident compound
C2442	LPG O-20:5	$C_{26}H_{45}O_8P$	6.90	516.29	8.16×10^{5}	3.28×10^{5}	6.29×10^{5}	2.05×10^{6}	5.28×10^{6}	3.93×10^{6}	6.35	2.67	2.90×10^{-2}	1.00	1.43	up	Confident compound
C2445	Ala-Glu	$C_8H_{14}N_2O_5$	0.57	218.09	1.22×10^{6}	1.20×10^{6}	9.23×10^{5}	4.39×10^{6}	5.52×10^{6}	2.50×10^{6}	3.70	1.89	2.72×10^{-2}	1.00	1.43	up	Confident compound

ID	Description	Formula	RT (min)	m/z	Model1	Model2	Model3	FCDPE1	FCDPE2	FCDPE3	FC	log2FC	P value	ROC	VIP	Up/Down	Annotation category
C2451	NP-005483	$C_{19}H_{18}O_3$	3.23	294.12	1.50×10^{6}	6.26×10^{5}	2.10×10^{6}	9.57×10^{6}	1.25×10^{7}	1.00×10^{7}	7.60	2.93	7.67×10^{-4}	1.00	1.62	up	Confident compound
C2456	(+)-Dihydrokavain	$C_{14}H_{16}O_3$	0.86	232.11	3.39×10^{6}	1.24×10^{6}	1.20×10^{6}	1.14×10^{7}	1.69×10^{7}	1.62×10^{7}	7.64	2.93	2.37×10^{-3}	1.00	1.58	up	Confident compound
C2465	LPG O-17:2	$C_{23}H_{45}O_8P$	7.02	480.29	1.82×10^{6}	3.02×10^{5}	7.84×10^{5}	2.50×10^{6}	3.58×10^{6}	2.77×10^{6}	3.04	1.61	2.33×10^{-2}	1.00	1.43	up	Confident compound
C2482	Glycyl-L-leucine	$C_8H_{16}N_2O_3$	2.06	188.12	2.26×10^{6}	8.28×10^{5}	1.86×10^{6}	6.11×10^{6}	8.85×10^{6}	6.73×10^{6}	4.38	2.13	3.92×10^{-3}	1.00	1.57	up	Confident compound
C2513	Ile-Thr	$C_{10}H_{20}N_2O_4$	0.90	232.14	1.97×10^{6}	4.14×10^{5}	5.42×10^{5}	3.63×10^{6}	9.89×10^{6}	7.38×10^{6}	7.14	2.84	3.37×10^{-2}	1.00	1.41	up	Confident compound
C2524	OIFCPZGZZQQDNO-UHFFFAOYSA-N	$C_{10}H_{16}O_4$	4.44	200.10	4.51×10^{6}	5.56×10^{6}	6.37×10^{6}	8.22×10^{6}	8.94×10^{6}	7.97×10^{6}	1.53	0.61	9.12×10^{-3}	1.00	1.53	up	Confident compound
C2530	PA 5:0_18:0	$C_{26}H_{51}O_8P$	8.01	522.34	1.54×10^{5}	1.61×10^{5}	2.71×10^{5}	3.47×10^{5}	3.80×10^{5}	3.28×10^{5}	1.80	0.85	1.88×10^{-2}	1.00	1.48	up	Confident compound
C2541	Ala-Glu-Ile	$C_{14}H_{25}N_3O_6$	3.36	331.17	8.04×10^{5}	1.07×10^{5}	9.04×10^{5}	4.38×10^{6}	8.44×10^{6}	9.93×10^{6}	12.53	3.65	1.42×10^{-2}	1.00	1.49	up	Confident compound
C2562	LPA 18:0	$C_{21}H_{43}O_7P$	7.98	438.27	4.18×10^{6}	1.06×10^{6}	9.06×10^{5}	6.96×10^{6}	7.77×10^{6}	1.31×10^{7}	4.54	2.18	3.06×10^{-2}	1.00	1.39	up	Confident compound
C2567	4-Acetamido-5-[2-(4-hydroxyphenyl)ethylamino]-5-oxopentanoic acid	$C_{15}H_{20}N_2O_5$	3.38	308.14	7.82×10^{5}	4.00×10^{5}	1.61×10^{6}	5.26×10^{6}	8.36×10^{6}	7.50×10^{6}	7.56	2.92	3.52×10^{-3}	1.00	1.58	up	Confident compound
C2579	Glu-Ile-Thr	$C_{15}H_{27}N_3O_7$	3.33	361.19	5.74×10^{5}	8.76×10^{4}	9.06×10^{5}	3.77×10^{6}	9.60×10^{6}	7.81×10^{6}	13.50	3.76	1.99×10^{-2}	1.00	1.47	up	Confident compound
C2591	LPG 18:2	$C_{24}H_{45}O_9P$	6.62	508.28	1.71×10^{6}	2.27×10^{6}	2.69×10^{6}	3.91×10^{6}	4.21×10^{6}	3.53×10^{6}	1.75	0.81	8.52×10^{-3}	1.00	1.54	up	Confident compound
C2598	1-[6-(benzyloxy)-3-(tert-butyl)-2-hydroxyphenyl]ethan-1-one	$C_{19}H_{22}O_3$	6.32	298.16	4.06×10^{6}	3.57×10^{6}	3.58×10^{6}	6.85×10^{6}	6.17×10^{6}	6.96×10^{6}	1.78	0.83	5.96×10^{-4}	1.00	1.60	up	Suspected contaminant
C2600	Aminocaproic acid	$C_6H_{13}NO_2$	2.06	131.09	2.98×10^{6}	2.35×10^{6}	2.43×10^{6}	6.81×10^{6}	4.28×10^{6}	4.58×10^{6}	2.02	1.01	3.28×10^{-2}	1.00	1.37	up	Confident compound
C2616	Cylindrol A1	$C_{25}H_{34}O_6$	3.76	430.24	8.15×10^{5}	8.17×10^{4}	3.71×10^{5}	3.03×10^{6}	4.96×10^{6}	5.64×10^{6}	10.75	3.43	7.04×10^{-3}	1.00	1.54	up	Confident compound
C2638	VANGVRQDFZGVJU-ZYUHXDNHSA-N	$C_{17}H_{22}O_3$	3.86	274.15	5.71×10^{5}	4.25×10^{5}	6.75×10^{5}	3.45×10^{6}	6.71×10^{6}	5.76×10^{6}	9.53	3.25	8.05×10^{-3}	1.00	1.54	up	Confident compound
C2640	Glu-Val	$C_{10}H_{18}N_2O_5$	2.05	246.12	1.16×10^{6}	2.45×10^{5}	6.29×10^{5}	5.34×10^{6}	6.25×10^{6}	5.22×10^{6}	8.27	3.05	3.00×10^{-4}	1.00	1.62	up	Confident compound
C2678	N-(1H-benzo[d]imidazol-2-yl)guanidine	$C_8H_9N_5$	0.80	175.08	2.98×10^{6}	3.23×10^{6}	3.62×10^{6}	5.36×10^{6}	4.85×10^{6}	4.62×10^{6}	1.51	0.59	4.38×10^{-3}	1.00	1.56	up	Suspected contaminant
C2680	Ala-Leu	$C_9H_{18}N_2O_3$	1.61	202.13	8.44×10^{5}	4.78×10^{5}	5.22×10^{5}	2.72×10^{6}	6.60×10^{6}	4.76×10^{6}	7.63	2.93	2.25×10^{-2}	1.00	1.46	up	Confident compound
C2683	Gly-Ala-Leu	$C_{11}H_{21}N_3O_4$	2.03	259.15	1.06×10^{6}	1.60×10^{5}	3.76×10^{5}	3.04×10^{6}	4.92×10^{6}	3.61×10^{6}	7.24	2.86	5.91×10^{-3}	1.00	1.55	up	Confident compound
C2695	Val-Phe	$C_{14}H_{20}N_2O_3$	3.37	264.15	7.77×10^{5}	2.46×10^{5}	8.07×10^{5}	2.57×10^{6}	3.91×10^{6}	3.13×10^{6}	5.25	2.39	3.83×10^{-3}	1.00	1.57	up	Confident compound
C2714	5-Methylcytidine	$C_{10}H_{15}N_3O_5$	0.76	257.10	3.16×10^{5}	1.94×10^{5}	2.87×10^{5}	9.42×10^{5}	7.97×10^{5}	1.06×10^{6}	3.51	1.81	1.34×10^{-3}	1.00	1.59	up	Confident compound
C2717	N-Acetylornithine	$C_7H_{14}N_2O_3$	0.54	174.10	2.30×10^{6}	1.44×10^{6}	1.35×10^{6}	2.83×10^{6}	3.53×10^{6}	3.73×10^{6}	1.98	0.99	1.51×10^{-2}	1.00	1.47	up	Confident compound
C2719	L-Methionine	$C_5H_{11}NO_2S$	0.58	149.05	9.85×10^{5}	1.47×10^{6}	2.25×10^{6}	4.83×10^{6}	4.29×10^{6}	4.95×10^{6}	2.99	1.58	1.77×10^{-3}	1.00	1.59	up	Confident compound
C2722	Met-Glu	$C_{10}H_{18}N_2O_5S$	1.39	278.09	8.49×10^{5}	2.67×10^{5}	3.17×10^{5}	2.53×10^{6}	7.24×10^{6}	6.73×10^{6}	11.51	3.53	2.88×10^{-2}	1.00	1.42	up	Confident compound

ID	Description	Formula	RT (min)	m/z	Model1	Model2	Model3	FCDPE1	FCDPE2	FCDPE3	FC	log2FC	P value	ROC	VIP	Up/Down	Annotation category
C2732	1-({(2R,3S,4R,5S)-3,4-Dihydroxy-5-[2-oxo-2-(1-pyrrolidinyl)ethyl]tetrahydro-2-furanyl}methyl)-3-isopropylurea	$C_{15}H_{27}N_3O_5$	3.85	329.20	1.53×10^6	1.51×10^5	5.10×10^5	3.82×10^6	4.09×10^6	4.35×10^6	5.60	2.48	1.58×10^{-3}	1.00	1.58	up	Suspected contaminant
C2735	LPG 17:1	$C_{23}H_{45}O_9P$	6.84	496.28	4.11×10^5	1.91×10^5	2.52×10^5	1.04×10^6	3.64×10^6	2.69×10^6	8.63	3.11	4.64×10^{-2}	1.00	1.36	up	Confident compound
C2741	1-O-[(3β,5ξ,9ξ,18ξ)-3,19,24-Trihydroxy-24,28-dioxours-12-en-28-yl]hexopyranose	$C_{36}H_{56}O_{11}$	6.97	664.37	1.60×10^6	2.76×10^5	2.88×10^5	1.75×10^6	2.01×10^6	2.39×10^6	2.84	1.51	4.97×10^{-2}	1.00	1.32	up	Confident compound
C2745	Glu-Gln-Ile	$C_{16}H_{28}N_4O_7$	3.30	388.20	4.44×10^5	3.53×10^5	7.19×10^5	2.41×10^6	6.27×10^6	4.66×10^6	8.80	3.14	2.49×10^{-2}	1.00	1.45	up	Confident compound
C2756	6β-Hydromorphol	$C_{17}H_{21}NO_3$	1.50	287.15	1.21×10^6	1.35×10^5	4.36×10^5	4.35×10^6	5.28×10^6	4.85×10^6	8.14	3.03	5.33×10^{-4}	1.00	1.61	up	Suspected contaminant
C2764	Alanylalanine	$C_6H_{12}N_2O_3$	0.75	160.08	1.79×10^6	9.09×10^5	5.48×10^5	2.35×10^6	4.19×10^6	4.13×10^6	3.29	1.72	2.49×10^{-2}	1.00	1.43	up	Confident compound
C2766	2-methyl-N-[4-(4-methylpiperazino)benzyl]benzenesulfonamide	$C_{19}H_{25}N_3O_2S$	3.11	359.17	6.85×10^5	3.59×10^5	3.04×10^5	1.95×10^6	4.52×10^6	3.61×10^6	7.48	2.90	1.88×10^{-2}	1.00	1.47	up	Suspected contaminant
C2769	Ala-Gln-Thr	$C_{12}H_{22}N_4O_6$	4.83	318.16	4.62×10^5	2.66×10^5	1.11×10^6	2.94×10^6	2.69×10^6	1.37×10^6	3.80	1.93	3.50×10^{-2}	1.00	1.39	up	Confident compound
C2775	Crotonic acid	$C_4H_6O_2$	0.80	86.04	1.80×10^6	1.92×10^6	1.36×10^6	4.10×10^6	2.96×10^6	3.04×10^6	1.99	0.99	1.44×10^{-2}	1.00	1.46	up	Confident compound
C2776	KQJGPGHQDDZVHJ-ZZXKWVIFSA-N	$C_{12}H_{20}O_5$	0.56	244.13	7.85×10^5	3.62×10^5	4.26×10^5	2.20×10^6	1.63×10^6	1.55×10^6	3.41	1.77	6.43×10^{-3}	1.00	1.52	up	Confident compound
C2784	SKLSOJOIYMIFGM-UHFFFAOYSA-N	$C_{16}H_{16}O_4$	1.47	272.10	1.75×10^6	9.68×10^5	8.88×10^5	2.08×10^6	3.14×10^6	3.14×10^6	2.32	1.21	2.39×10^{-2}	1.00	1.43	up	Confident compound
C2799	LEE	$C_{16}H_{27}N_3O_8$	2.86	389.18	5.33×10^5	8.85×10^4	2.23×10^5	1.37×10^6	4.13×10^6	4.32×10^6	11.62	3.54	3.58×10^{-2}	1.00	1.40	up	Confident compound
C2801	Val-Gly	$C_7H_{14}N_2O_3$	0.93	174.10	1.37×10^6	5.62×10^5	5.06×10^5	2.55×10^6	3.95×10^6	3.56×10^6	4.12	2.04	7.29×10^{-3}	1.00	1.53	up	Confident compound
C2805	Cyclo(Leu-Phe)	$C_{15}H_{20}N_2O_2$	4.22	260.15	3.65×10^5	1.13×10^5	3.08×10^5	2.16×10^6	2.32×10^6	1.91×10^6	8.12	3.02	1.89×10^{-4}	1.00	1.63	up	Confident compound
C2814	2-[(2-{[(Benzyloxy)carbonyl]amino}-3-methylpentanoyl)amino]acetic acid	$C_{16}H_{22}N_2O_5$	3.28	322.15	1.24×10^6	1.68×10^6	2.05×10^6	2.14×10^6	2.76×10^6	2.83×10^6	1.56	0.64	4.60×10^{-2}	1.00	1.37	up	Suspected contaminant
C2825	Isopropyl 4-hydroxybenzoate	$C_{10}H_{12}O_3$	4.38	180.08	3.86×10^5	2.11×10^5	1.33×10^5	6.47×10^5	4.86×10^5	5.99×10^5	2.38	1.25	1.96×10^{-2}	1.00	1.43	up	Suspected contaminant
C2839	Gln-Leu-Ile	$C_{17}H_{32}N_4O_5$	3.71	372.24	2.31×10^5	1.34×10^5	2.20×10^5	1.94×10^6	1.20×10^6	1.22×10^6	7.44	2.89	6.82×10^{-3}	1.00	1.52	up	Confident compound
C2860	L-Histidine	$C_6H_9N_3O_2$	0.53	155.07	5.03×10^5	4.18×10^5	3.98×10^5	1.45×10^6	2.34×10^6	1.68×10^6	4.15	2.05	6.75×10^{-3}	1.00	1.54	up	Confident compound
C2879	Guanine	$C_5H_5N_5O$	0.59	151.05	6.07×10^5	6.10×10^5	1.28×10^6	3.01×10^6	2.33×10^6	2.92×10^6	3.30	1.72	3.47×10^{-3}	1.00	1.56	up	Confident compound
C2891	Lys-Val-Pro	$C_{16}H_{30}N_4O_4$	3.25	342.23	6.34×10^5	2.51×10^5	2.13×10^5	2.37×10^6	4.49×10^6	4.10×10^6	9.97	3.32	7.83×10^{-3}	1.00	1.53	up	Confident compound
C2892	3-O-Methylcytidine	$C_{10}H_{15}N_3O_5$	1.04	257.10	1.77×10^5	1.35×10^5	1.55×10^5	5.93×10^5	8.85×10^5	8.28×10^5	4.94	2.31	2.43×10^{-3}	1.00	1.59	up	Confident compound
C2913	Leu-Thr-Leu	$C_{16}H_{31}N_3O_5$	3.73	345.23	2.96×10^5	8.70×10^4	2.12×10^5	1.07×10^6	8.47×10^5	4.88×10^5	4.04	2.01	2.83×10^{-2}	1.00	1.41	up	Confident compound
C2914	LPA 24:1	$C_{27}H_{53}O_7P$	8.64	520.36	4.23×10^5	1.55×10^5	1.48×10^5	5.10×10^5	6.49×10^5	9.47×10^5	2.90	1.54	4.34×10^{-2}	1.00	1.35	up	Confident compound
C2934	2-[(3S)-1-(Cyclohexylmethyl)-3-pyrrolidinyl]-5-fluoro-1H-benzimidazole	$C_{18}H_{24}FN_3$	2.90	301.20	4.09×10^5	1.14×10^5	1.23×10^5	9.81×10^5	1.75×10^6	1.41×10^6	6.40	2.68	8.55×10^{-3}	1.00	1.53	up	Suspected contaminant
C2947	Ala-Gln-Leu	$C_{14}H_{26}N_4O_5$	2.88	330.19	1.49×10^5	4.37×10^4	1.66×10^5	1.27×10^6	2.21×10^6	1.61×10^6	14.14	3.82	4.65×10^{-3}	1.00	1.56	up	Confident compound

ID	Description	Formula	RT (min)	m/z	Model1	Model2	Model3	FCDPE1	FCDPE2	FCDPE3	FC	log2FC	P value	ROC	VIP	Up/Down	Annotation category
C2957	Phe-Val	$C_{14}H_{20}N_2O_3$	3.15	264.15	4.15×10^{5}	1.62×10^{5}	3.41×10^{5}	1.44×10^{6}	1.68×10^{6}	8.57×10^{5}	4.34	2.12	1.63×10^{-2}	1.00	1.48	up	Confident compound
C2968	Ile-Tyr	$C_{15}H_{22}N_2O_4$	3.07	294.16	4.49×10^{5}	2.19×10^{5}	5.25×10^{5}	1.77×10^{6}	2.06×10^{6}	1.68×10^{6}	4.62	2.21	6.16×10^{-4}	1.00	1.62	up	Confident compound
C2977	Ile-Gly-Ile	$C_{14}H_{27}N_3O_4$	3.44	301.20	3.32×10^{5}	4.45×10^{4}	2.82×10^{5}	8.55×10^{5}	1.58×10^{6}	1.21×10^{6}	5.54	2.47	1.17×10^{-2}	1.00	1.51	up	Confident compound
C2979	Gln-Val-Ala	$C_{13}H_{24}N_4O_5$	0.80	316.17	1.49×10^{5}	1.02×10^{5}	1.28×10^{5}	1.61×10^{6}	2.57×10^{6}	2.40×10^{6}	17.34	4.12	2.20×10^{-3}	1.00	1.59	up	Confident compound
C2984	N1-(3-Chlorobenzyl)-2-(1-adamantyl)acetamide	$C_{19}H_{24}ClNO$	2.83	317.16	2.39×10^{5}	1.02×10^{5}	1.21×10^{5}	1.58×10^{6}	2.96×10^{6}	1.94×10^{6}	14.02	3.81	8.40×10^{-3}	1.00	1.53	up	Suspected contaminant
C2989	[(6Z,10Z)-6-(acetyloxymethyl)-10-(hydroperoxymethyl)-3-methylidene-2-oxo-3a,4,5,8,9,11a-hexahydrocyclodeca[b]furan-4-yl] 2-methylbutanoate	$C_{22}H_{30}O_8$	3.62	422.20	1.75×10^{5}	5.61×10^{4}	1.75×10^{5}	8.20×10^{5}	3.16×10^{6}	2.20×10^{6}	15.22	3.93	4.74×10^{-2}	1.00	1.36	up	Confident compound
C2991	Gln-Glu-Ile	$C_{16}H_{28}N_4O_7$	2.88	388.20	2.56×10^{5}	9.77×10^{4}	2.98×10^{5}	1.26×10^{6}	2.43×10^{6}	1.95×10^{6}	8.66	3.11	8.63×10^{-3}	1.00	1.53	up	Confident compound
C2993	Phe-Gly-Ile	$C_{17}H_{25}N_3O_4$	3.60	335.18	1.44×10^{5}	5.07×10^{4}	6.78×10^{4}	2.03×10^{6}	2.27×10^{6}	1.85×10^{6}	23.47	4.55	9.81×10^{-5}	1.00	1.63	up	Confident compound
C3010	Ile-Gln-Pro	$C_{16}H_{28}N_4O_5$	3.46	356.21	1.74×10^{5}	1.18×10^{5}	1.20×10^{5}	8.53×10^{5}	1.71×10^{6}	1.68×10^{6}	10.30	3.36	1.05×10^{-2}	1.00	1.52	up	Confident compound
C3068	Vinyl 6,6-dimethylheptanoate	$C_{11}H_{20}O_2$	5.10	184.15	2.93×10^{5}	3.93×10^{5}	3.23×10^{5}	6.72×10^{5}	1.48×10^{6}	1.29×10^{6}	3.41	1.77	3.01×10^{-2}	1.00	1.42	up	Suspected contaminant
C3086	Gly-Pro-Leu	$C_{13}H_{23}N_3O_4$	3.93	285.17	1.04×10^{5}	1.14×10^{5}	8.36×10^{4}	2.44×10^{5}	2.94×10^{5}	3.34×10^{5}	2.90	1.53	2.35×10^{-3}	1.00	1.58	up	Confident compound
C3099	Glu-Ile-Ile	$C_{17}H_{31}N_3O_6$	4.07	373.22	1.85×10^{5}	6.58×10^{4}	1.65×10^{5}	9.36×10^{5}	2.28×10^{6}	1.71×10^{6}	11.82	3.56	1.83×10^{-2}	1.00	1.48	up	Confident compound
C3100	Asn-Glu-Leu	$C_{15}H_{26}N_4O_7$	2.91	374.18	1.32×10^{5}	3.93×10^{4}	8.21×10^{4}	9.67×10^{5}	1.52×10^{6}	1.14×10^{6}	14.31	3.84	2.47×10^{-3}	1.00	1.59	up	Confident compound
C3121	LPA 17:0	$C_{20}H_{41}O_7P$	7.60	424.26	5.44×10^{5}	1.36×10^{5}	1.08×10^{5}	7.85×10^{5}	1.24×10^{6}	2.03×10^{6}	5.14	2.36	4.92×10^{-2}	1.00	1.33	up	Confident compound
C3128	Paracetamol sulfate	$C_8H_9NO_5S$	2.78	231.02	2.51×10^{4}	2.48×10^{4}	2.75×10^{4}	4.53×10^{4}	4.07×10^{4}	3.92×10^{4}	1.62	0.69	1.32×10^{-3}	1.00	1.59	up	Suspected contaminant
C3149	Leu-Leu-Ile	$C_{18}H_{35}N_3O_4$	4.09	357.26	2.00×10^{5}	4.39×10^{4}	6.36×10^{4}	3.72×10^{5}	9.16×10^{5}	8.24×10^{5}	6.87	2.78	2.63×10^{-2}	1.00	1.43	up	Confident compound
C3152	8-iso Prostaglandin F2α Ethanolamide	$C_{22}H_{39}NO_5$	8.06	397.28	5.99×10^{4}	3.69×10^{4}	3.67×10^{4}	6.56×10^{4}	6.91×10^{4}	7.32×10^{4}	1.56	0.64	3.60×10^{-2}	1.00	1.37	up	Confident compound
C3156	N4-(2-{[(cyclopropylmethyl)amino]carbonyl}phenyl)-5-methyl-2-phenyl-2H-1,2,3-triazole-4-carboxamide	$C_{21}H_{21}N_5O_2$	2.08	375.16	1.24×10^{5}	5.14×10^{4}	5.47×10^{4}	4.11×10^{5}	9.60×10^{5}	8.73×10^{5}	9.75	3.29	1.75×10^{-2}	1.00	1.48	up	Suspected contaminant
C3170	N-{4-[(R)-[(3R)-4-(4-Fluorobenzyl)-5-oxo-3-morpholinyl](hydroxy)methyl]phenyl}acetamide	$C_{20}H_{21}FN_2O_4$	4.57	372.16	6.19×10^{4}	5.28×10^{4}	5.81×10^{4}	3.53×10^{5}	4.32×10^{5}	3.26×10^{5}	6.42	2.68	6.08×10^{-4}	1.00	1.62	up	Suspected contaminant
C3171	Thr-Thr-Leu	$C_{14}H_{27}N_3O_6$	2.97	333.19	3.76×10^{4}	1.96×10^{4}	2.37×10^{4}	3.42×10^{5}	4.72×10^{5}	3.59×10^{5}	14.51	3.86	8.91×10^{-4}	1.00	1.61	up	Confident compound
C3175	Tiapride	$C_{15}H_{24}N_2O_4S$	3.53	328.14	6.01×10^{4}	4.07×10^{4}	1.91×10^{5}	4.87×10^{5}	6.05×10^{5}	3.78×10^{5}	5.04	2.33	8.27×10^{-3}	1.00	1.53	up	Suspected contaminant
C3185	Gln-Gly-Leu	$C_{13}H_{24}N_4O_5$	2.64	316.17	5.70×10^{4}	2.70×10^{4}	5.37×10^{4}	5.64×10^{5}	4.87×10^{5}	4.33×10^{5}	10.78	3.43	3.30×10^{-4}	1.00	1.62	up	Confident compound
C3188	Thr-Val-Ile	$C_{15}H_{29}N_3O_5$	3.48	331.21	1.01×10^{5}	3.56×10^{4}	9.16×10^{4}	2.62×10^{5}	8.50×10^{5}	6.31×10^{5}	7.64	2.93	4.32×10^{-2}	1.00	1.38	up	Confident compound
C3193	Oxydi-1,2-propanediyl tetraphenyl bis(phosphite)	$C_{30}H_{32}O_7P_2$	3.35	566.16	4.39×10^{4}	5.16×10^{4}	5.35×10^{4}	6.87×10^{4}	7.90×10^{4}	7.79×10^{4}	1.52	0.60	4.29×10^{-3}	1.00	1.57	up	Suspected contaminant
C3205	NP-024154	$C_{17}H_{18}O_5$	2.87	302.11	1.10×10^{5}	2.29×10^{4}	2.57×10^{4}	2.56×10^{5}	4.09×10^{5}	3.63×10^{5}	6.50	2.70	5.60×10^{-3}	1.00	1.55	up	Confident compound

ID	Description	Formula	RT (min)	m/z	Model1	Model2	Model3	FCDPE1	FCDPE2	FCDPE3	FC	log2FC	P value	ROC	VIP	Up/Down	Annotation category
C3213	Ser-Arg-Asn	$C_{13}H_{25}N_7O_6$	4.47	375.18	6.10×10^4	6.91×10^4	1.02×10^5	8.12×10^5	9.23×10^5	6.54×10^5	10.30	3.36	8.11×10^{-4}	1.00	1.61	up	Confident compound
C3219	Citral	$C_{10}H_{16}O$	3.69	152.12	4.26×10^4	2.73×10^4	3.55×10^4	5.75×10^4	6.36×10^4	5.10×10^4	1.63	0.71	1.80×10^{-2}	1.00	1.46	up	Confident compound
C3250	CIGSWLXZMSXAAE-ZCFIWIBFSA-N	$C_{14}H_{14}N_2O_4$	4.18	274.10	1.18×10^5	6.60×10^4	8.26×10^4	6.20×10^5	7.42×10^5	7.05×10^5	7.74	2.95	1.08×10^{-4}	1.00	1.63	up	Confident compound
C3253	(2R)-5-Methoxy-8,8-dimethyl-2-phenyl-2,3-dihydro-4H,8H-pyrano[2,3-f]chromen-4-one	$C_{21}H_{20}O_4$	2.98	336.14	5.84×10^4	8.91×10^4	9.00×10^4	2.79×10^5	2.63×10^5	1.40×10^5	2.87	1.52	3.00×10^{-2}	1.00	1.41	up	Confident compound
C3263	Pro-Ala-Thr	$C_{12}H_{21}N_3O_5$	3.30	287.15	5.64×10^4	2.52×10^4	5.73×10^4	6.37×10^5	7.02×10^5	6.06×10^5	13.99	3.81	3.80×10^{-5}	1.00	1.64	up	Confident compound
C3295	Pro-Val-Leu	$C_{16}H_{29}N_3O_4$	3.43	327.22	5.01×10^4	4.13×10^4	4.33×10^4	1.12×10^5	7.21×10^4	7.23×10^4	1.90	0.93	3.93×10^{-2}	1.00	1.35	up	Confident compound
C3304	Gln-Gln-Leu	$C_{16}H_{29}N_5O_6$	2.95	387.21	1.33×10^4	1.57×10^4	1.54×10^4	1.47×10^5	6.77×10^5	6.10×10^5	32.34	5.02	4.97×10^{-2}	1.00	1.35	up	Confident compound

Supplementary Table S2. Differential metabolites in the DCDPE group compared with the model group.

ID	Description	Formula	RT (min)	m/z	Model1	Model2	Model3	DCDPE1	DCDPE2	DCDPE3	FC	log2FC	P value	ROC	VIP	Up/Down	Annotation category
C41	1,4-dihydroxyheptadec-16-en-2-yl acetate	$C_{19}H_{36}O_4$	6.93	328.26	1.62×10^{8}	1.64×10^{8}	1.74×10^{8}	9.68×10^{7}	4.78×10^{7}	8.80×10^{7}	0.47	-1.10	4.62×10^{-3}	1.00	1.76	down	Confident compound
C104	Dicyclomine	$C_{19}H_{35}NO_2$	6.87	309.27	8.85×10^{7}	1.47×10^{8}	1.24×10^{8}	1.43×10^{7}	3.71×10^{7}	2.06×10^{7}	0.20	-2.32	6.27×10^{-3}	1.00	1.73	down	Suspected contaminant
C107	Stigmasterol	$C_{29}H_{48}O$	12.97	412.37	1.23×10^{8}	1.33×10^{8}	1.08×10^{8}	6.16×10^{7}	6.85×10^{7}	5.47×10^{7}	0.51	-0.98	1.96×10^{-3}	1.00	1.81	down	Confident compound
C134	Hypoxanthine	$C_5H_4N_4O$	0.56	136.04	5.26×10^{8}	1.01×10^{9}	1.28×10^{9}	6.42×10^{7}	2.88×10^{8}	1.08×10^{8}	0.16	-2.61	2.73×10^{-2}	1.00	1.59	down	Confident compound
C148	Pinolenic acid	$C_{18}H_{30}O_2$	6.94	278.22	3.70×10^{7}	3.79×10^{7}	4.14×10^{7}	2.43×10^{7}	1.36×10^{7}	2.19×10^{7}	0.51	-0.96	5.89×10^{-3}	1.00	1.74	down	Confident compound
C164	D-Sphingosine	$C_{18}H_{37}NO_2$	6.96	299.28	9.12×10^{7}	5.55×10^{7}	5.15×10^{7}	5.55×10^{6}	1.43×10^{7}	3.54×10^{7}	0.28	-1.84	3.64×10^{-2}	1.00	1.64	down	Confident compound
C165	LysoPC(O-18:0/0:0)	$C_{26}H_{56}NO_6P$	8.30	509.39	8.04×10^{7}	7.12×10^{7}	6.37×10^{7}	1.87×10^{7}	2.81×10^{7}	4.35×10^{7}	0.42	-1.25	8.68×10^{-3}	1.00	1.77	down	Confident compound
C197	1,1-Bi(cyclohexyl)	$C_{12}H_{22}$	8.10	166.17	5.80×10^{7}	7.60×10^{7}	5.60×10^{7}	1.16×10^{7}	7.98×10^{6}	1.59×10^{7}	0.19	-2.42	1.60×10^{-3}	1.00	1.80	down	Suspected contaminant
C200	Tiamulin	$C_{28}H_{47}NO_4S$	6.70	493.32	5.94×10^{6}	8.90×10^{6}	7.93×10^{6}	2.07×10^{6}	5.73×10^{6}	2.96×10^{6}	0.47	-1.08	4.65×10^{-2}	1.00	1.52	down	Suspected contaminant
C253	NAGly 15:0/16:0	$C_{33}H_{63}NO_5$	12.88	553.47	4.79×10^{7}	3.70×10^{7}	4.75×10^{7}	2.18×10^{7}	3.63×10^{7}	1.63×10^{7}	0.56	-0.83	4.92×10^{-2}	1.00	1.57	down	Confident compound
C262	13-HODE	$C_{18}H_{32}O_3$	6.34	296.24	3.44×10^{7}	2.07×10^{7}	4.37×10^{7}	1.18×10^{7}	1.43×10^{7}	4.28×10^{6}	0.31	-1.70	3.56×10^{-2}	1.00	1.61	down	Confident compound
C313	PI-Cer 35:0;3O	$C_{41}H_{82}NO_{12}P$	13.08	811.56	3.70×10^{7}	2.45×10^{7}	2.43×10^{7}	5.63×10^{6}	5.16×10^{6}	5.29×10^{6}	0.19	-2.42	5.18×10^{-3}	1.00	1.81	down	Confident compound
C320	LysoPC(26:0/0:0)	$C_{34}H_{70}NO_7P$	13.05	635.49	2.02×10^{7}	4.05×10^{7}	1.67×10^{7}	1.31×10^{6}	1.14×10^{6}	1.20×10^{6}	0.05	-4.41	2.99×10^{-2}	1.00	1.57	down	Confident compound
C329	Nicotinic acid ribonucleoside	$C_{11}H_{14}NO_6$	0.52	256.08	2.14×10^{7}	3.47×10^{7}	2.32×10^{7}	9.70×10^{6}	7.22×10^{6}	1.27×10^{7}	0.37	-1.42	2.06×10^{-2}	1.00	1.61	down	Confident compound
C376	N-(2-Hydroxyethyl)-9-octadecenamide	$C_{20}H_{39}NO_2$	8.80	325.30	3.19×10^{7}	2.63×10^{7}	1.03×10^{7}	4.85×10^{6}	2.91×10^{6}	2.83×10^{6}	0.15	-2.70	4.10×10^{-2}	1.00	1.58	down	Confident compound
C445	N,N-Dimethyloctadecanamide	$C_{20}H_{41}NO$	9.96	311.32	2.53×10^{7}	2.73×10^{7}	2.52×10^{7}	1.21×10^{7}	2.07×10^{7}	1.29×10^{7}	0.59	-0.77	1.96×10^{-2}	1.00	1.67	down	Suspected contaminant
C466	HexCer 18:2;2O/16:0;O	$C_{40}H_{75}NO_9$	11.00	713.54	9.67×10^{6}	8.35×10^{6}	5.40×10^{6}	2.92×10^{6}	4.61×10^{6}	3.89×10^{6}	0.49	-1.04	4.19×10^{-2}	1.00	1.59	down	Confident compound
C472	QOLRLLFJMZLYQJ-KVERTZGMSA-N	$C_{27}H_{42}O_4$	7.18	430.30	2.01×10^{7}	1.53×10^{7}	1.94×10^{7}	5.89×10^{6}	5.95×10^{6}	5.26×10^{6}	0.31	-1.68	1.18×10^{-3}	1.00	1.85	down	Confident compound
C501	N,N-Dimethylsphingosine	$C_{20}H_{41}NO_2$	7.75	327.31	1.58×10^{7}	9.61×10^{6}	7.89×10^{6}	1.21×10^{6}	2.28×10^{6}	5.54×10^{6}	0.27	-1.88	4.12×10^{-2}	1.00	1.62	down	Confident compound
C521	PI-Cer 33:0;3O	$C_{39}H_{78}NO_{12}P$	13.09	783.53	2.15×10^{7}	1.21×10^{7}	1.08×10^{7}	1.85×10^{6}	5.01×10^{6}	1.71×10^{6}	0.19	-2.37	2.74×10^{-2}	1.00	1.67	down	Confident compound
C538	LysoPI(18:0/0:0)	$C_{27}H_{53}O_{12}P$	7.41	600.33	1.36×10^{7}	9.37×10^{6}	1.32×10^{7}	2.76×10^{6}	8.38×10^{6}	2.35×10^{6}	0.37	-1.42	3.32×10^{-2}	1.00	1.64	down	Confident compound
C580	Cer 20:0;2O/2:0	$C_{22}H_{45}NO_3$	8.34	371.34	9.75×10^{6}	9.12×10^{6}	9.21×10^{6}	4.24×10^{6}	3.32×10^{6}	2.01×10^{6}	0.34	-1.55	7.96×10^{-4}	1.00	1.84	down	Confident compound
C649	Cholest-4-en-3-one	$C_{27}H_{44}O$	11.88	384.34	1.56×10^{7}	1.31×10^{7}	9.14×10^{6}	6.27×10^{6}	7.34×10^{6}	5.50×10^{6}	0.50	-0.99	3.33×10^{-2}	1.00	1.62	down	Confident compound
C706	Methyl caffeate	$C_{10}H_{10}O_4$	3.76	194.06	4.17×10^{6}	4.21×10^{6}	4.93×10^{6}	9.64×10^{5}	2.35×10^{6}	1.58×10^{6}	0.37	-1.45	3.96×10^{-3}	1.00	1.80	down	Confident compound
C743	2-(3-hydroxy-3,7,11,15-tetramethylhexadecyl)-3,5,6-trimethylcyclohexa-2,5-diene-1,4-dione	$C_{29}H_{50}O_3$	11.84	446.38	7.13×10^{6}	9.65×10^{6}	9.77×10^{6}	3.95×10^{6}	3.81×10^{6}	6.75×10^{6}	0.55	-0.87	3.56×10^{-2}	1.00	1.56	down	Confident compound

ID	Description	Formula	RT (min)	m/z	Model1	Model2	Model3	DCDPE1	DCDPE2	DCDPE3	FC	log2FC	P value	ROC	VIP	Up/Down	Annotation category
C755	LysoPC(18:1(9Z)/0:0)	$C_{26}H_{52}NO_7P$	8.61	521.35	8.40×10^{6}	1.31×10^{7}	8.24×10^{6}	3.64×10^{6}	5.53×10^{6}	3.75×10^{6}	0.44	-1.20	2.98×10^{-2}	1.00	1.57	down	Confident compound
C867	Cer 18:0;2O/18:2	$C_{36}H_{69}NO_3$	12.73	563.53	4.76×10^{6}	7.19×10^{6}	4.54×10^{6}	1.80×10^{6}	2.26×10^{6}	2.30×10^{6}	0.39	-1.38	1.75×10^{-2}	1.00	1.64	down	Confident compound
C889	3-Methyl-5-(5,5,8a-trimethyl-2-methylene-7-oxodecahydro-1-naphthalenyl)pentyl acetate	$C_{22}H_{36}O_3$	7.77	348.27	5.10×10^{6}	4.86×10^{6}	3.73×10^{6}	2.34×10^{6}	2.74×10^{6}	2.68×10^{6}	0.57	-0.82	1.08×10^{-2}	1.00	1.74	down	Confident compound
C902	Pentadecanoyl Ethanolamide	$C_{17}H_{35}NO_2$	7.86	285.27	5.08×10^{6}	4.40×10^{6}	2.75×10^{6}	5.33×10^{5}	5.81×10^{5}	1.18×10^{6}	0.19	-2.42	1.01×10^{-2}	1.00	1.75	down	Confident compound
C949	Trihexyl 1,2,4-benzenetricarboxylate	$C_{27}H_{42}O_6$	8.96	462.30	8.58×10^{6}	4.78×10^{6}	5.16×10^{6}	1.87×10^{6}	1.21×10^{6}	3.29×10^{6}	0.34	-1.54	4.04×10^{-2}	1.00	1.62	down	Suspected contaminant
C972	2-Arachidonoyl glycerol	$C_{23}H_{38}O_4$	9.26	378.28	6.98×10^{6}	7.93×10^{6}	8.02×10^{6}	3.75×10^{6}	2.45×10^{6}	3.32×10^{6}	0.41	-1.27	8.94×10^{-4}	1.00	1.81	down	Confident compound
C978	(2E,4E,14E)-13-Hydroperoxy-N-(2-methylpropyl)icosa-2,4,14-trienamide	$C_{24}H_{43}NO_3$	8.69	393.32	3.84×10^{6}	4.19×10^{6}	3.94×10^{6}	1.81×10^{6}	8.61×10^{5}	2.80×10^{6}	0.46	-1.13	1.90×10^{-2}	1.00	1.66	down	Confident compound
C1017	Cer 17:0;2O/2:0	$C_{19}H_{39}NO_3$	7.17	329.29	6.50×10^{6}	5.85×10^{6}	2.04×10^{6}	8.91×10^{5}	5.28×10^{5}	4.60×10^{5}	0.13	-2.94	4.05×10^{-2}	1.00	1.57	down	Confident compound
C1061	MG(0:0/14:1(9Z)/0:0)	$C_{17}H_{32}O_4$	6.25	300.23	5.09×10^{6}	6.18×10^{6}	6.81×10^{6}	2.36×10^{6}	9.01×10^{5}	2.25×10^{6}	0.30	-1.72	3.66×10^{-3}	1.00	1.76	down	Confident compound
C1084	NP-011223	$C_{10}H_{18}O_3$	6.92	186.13	2.19×10^{6}	2.42×10^{6}	2.93×10^{6}	1.82×10^{6}	8.01×10^{5}	1.46×10^{6}	0.54	-0.89	3.59×10^{-2}	1.00	1.55	down	Confident compound
C1086	Pro-Arg-Ala	$C_{14}H_{26}N_6O_4$	3.90	342.20	4.52×10^{6}	3.70×10^{6}	5.03×10^{6}	2.38×10^{6}	2.95×10^{6}	1.97×10^{6}	0.55	-0.86	1.44×10^{-2}	1.00	1.72	down	Confident compound
C1127	2,2-Methylenebis[4-methyl-6-(1-methylcyclohexyl)phenol]	$C_{29}H_{40}O_2$	8.08	420.30	4.02×10^{6}	4.09×10^{6}	3.02×10^{6}	2.91×10^{6}	1.79×10^{6}	2.43×10^{6}	0.64	-0.64	4.86×10^{-2}	1.00	1.51	down	Suspected contaminant
C1135	VCJZLHSIECUOIT-NSHDSACASA-N	$C_{13}H_{16}N_2O_2$	4.62	232.12	3.71×10^{6}	2.41×10^{6}	3.93×10^{6}	1.79×10^{6}	1.92×10^{6}	1.29×10^{6}	0.50	-1.01	2.99×10^{-2}	1.00	1.65	down	Confident compound
C1145	AUDA	$C_{23}H_{40}N_2O_3$	10.96	392.31	6.00×10^{6}	4.71×10^{6}	4.42×10^{6}	2.92×10^{6}	3.56×10^{6}	3.17×10^{6}	0.64	-0.65	2.49×10^{-2}	1.00	1.69	down	Suspected contaminant
C1174	HexCer 18:2;2O/16:1	$C_{40}H_{73}NO_8$	10.97	695.53	2.63×10^{6}	2.34×10^{6}	1.42×10^{6}	9.58×10^{5}	1.19×10^{6}	3.95×10^{5}	0.40	-1.33	4.22×10^{-2}	1.00	1.57	down	Confident compound
C1192	7-Oxocholesterol	$C_{27}H_{44}O_2$	9.29	400.33	1.77×10^{6}	3.29×10^{6}	2.40×10^{6}	7.93×10^{5}	3.28×10^{5}	1.22×10^{6}	0.31	-1.67	2.90×10^{-2}	1.00	1.56	down	Confident compound
C1193	trans-Hexadec-2-enoyl carnitine	$C_{23}H_{43}NO_4$	9.24	397.32	1.82×10^{6}	3.42×10^{6}	2.40×10^{6}	7.93×10^{5}	3.48×10^{5}	1.22×10^{6}	0.31	-1.69	2.98×10^{-2}	1.00	1.56	down	Confident compound
C1194	Gln-Arg-Asn	$C_{15}H_{28}N_8O_6$	2.83	416.21	4.26×10^{5}	5.94×10^{5}	6.61×10^{5}	1.41×10^{5}	1.43×10^{5}	1.77×10^{5}	0.27	-1.87	4.55×10^{-3}	1.00	1.75	down	Confident compound
C1200	4,8-Dihydroxy-7-(hydroxymethyl)-6-methoxy-3,4-dihydro-1(2H)-naphthalenone	$C_{12}H_{14}O_5$	4.65	238.08	7.22×10^{5}	7.66×10^{5}	9.07×10^{5}	2.19×10^{5}	3.50×10^{5}	1.36×10^{5}	0.29	-1.76	2.53×10^{-3}	1.00	1.80	down	Confident compound
C1213	2-{5-[2-({2-[5-(2-Hydroxypropyl)tetrahydro-2-furanyl]propanoyl}oxy)propyl]tetrahydro-2-furanyl}propanoic acid	$C_{20}H_{34}O_7$	4.33	386.23	1.63×10^{6}	1.46×10^{6}	1.43×10^{6}	8.49×10^{5}	8.20×10^{5}	6.72×10^{5}	0.52	-0.95	8.71×10^{-4}	1.00	1.85	down	Confident compound
C1221	4-Acetylphenetole	$C_{10}H_{12}O_2$	8.93	164.08	3.52×10^{6}	3.16×10^{6}	4.28×10^{6}	2.01×10^{6}	1.84×10^{6}	1.16×10^{6}	0.46	-1.13	9.27×10^{-3}	1.00	1.73	down	Confident compound
C1252	NP-015980	$C_{14}H_{24}O_6$	4.08	288.16	7.30×10^{5}	1.57×10^{6}	1.75×10^{6}	3.45×10^{5}	5.59×10^{5}	4.14×10^{5}	0.32	-1.62	4.71×10^{-2}	1.00	1.49	down	Confident compound
C1262	HexCer 18:1;2O/14:0	$C_{38}H_{73}NO_8$	9.57	671.53	2.68×10^{6}	3.74×10^{6}	3.11×10^{6}	1.86×10^{6}	2.18×10^{6}	1.23×10^{6}	0.55	-0.85	2.73×10^{-2}	1.00	1.58	down	Confident compound
C1293	NP-017176	$C_{27}H_{40}O_5$	9.32	444.28	2.07×10^{6}	1.69×10^{6}	1.57×10^{6}	5.04×10^{5}	1.08×10^{6}	7.98×10^{5}	0.45	-1.16	1.15×10^{-2}	1.00	1.76	down	Confident compound
C1301	Tris(2-ethylhexyl) phosphite	$C_{24}H_{51}O_3P$	8.38	418.36	3.64×10^{6}	2.74×10^{6}	2.79×10^{6}	1.39×10^{6}	1.83×10^{6}	2.21×10^{6}	0.59	-0.75	2.97×10^{-2}	1.00	1.67	down	Suspected contaminant

ID	Description	Formula	RT (min)	m/z	Model1	Model2	Model3	DCDPE1	DCDPE2	DCDPE3	FC	log2FC	P value	ROC	VIP	Up/Down	Annotation category
C1305	Mebeverine	$C_{25}H_{35}NO_5$	6.82	429.25	1.49×10^{6}	1.81×10^{6}	1.44×10^{6}	8.63×10^{5}	1.16×10^{6}	5.23×10^{5}	0.54	-0.90	2.87×10^{-2}	1.00	1.59	down	Suspected contaminant
C1356	Methyl N-{[(2S)-1-(cyclohexylcarbamoyl)-4-(4-piperidinylcarbonyl)-2-piperazinyl]carbonyl}-D-alaninate	$C_{22}H_{37}N_5O_5$	7.40	451.28	2.86×10^{6}	3.10×10^{6}	2.99×10^{6}	2.34×10^{6}	1.12×10^{6}	1.39×10^{6}	0.54	-0.88	2.26×10^{-2}	1.00	1.61	down	Suspected contaminant
C1374	3-Hydroxy-4,7,7-trimethylbicyclo[2.2.1]heptan-2-one	$C_{10}H_{16}O_2$	6.67	168.11	2.19×10^{6}	1.18×10^{6}	2.97×10^{6}	4.63×10^{5}	5.18×10^{5}	4.26×10^{5}	0.22	-2.17	3.43×10^{-2}	1.00	1.62	down	Confident compound
C1382	Ethylbenzoate	$C_9H_{10}O_2$	9.23	150.07	2.77×10^{6}	1.20×10^{6}	2.29×10^{6}	8.60×10^{5}	6.08×10^{5}	1.57×10^{5}	0.26	-1.95	3.80×10^{-2}	1.00	1.62	down	Confident compound
C1450	8-Isoprostaglandin F1β	$C_{20}H_{36}O_5$	7.03	356.26	2.22×10^{6}	2.30×10^{6}	2.33×10^{6}	1.83×10^{6}	1.06×10^{6}	1.16×10^{6}	0.59	-0.76	1.84×10^{-2}	1.00	1.63	down	Confident compound
C1478	Ergocalciferol	$C_{28}H_{44}O$	11.86	396.34	3.05×10^{6}	3.22×10^{6}	2.77×10^{6}	2.02×10^{6}	2.05×10^{6}	1.87×10^{6}	0.66	-0.61	1.94×10^{-3}	1.00	1.80	down	Confident compound
C1480	2,6-Dimethyl-4-nonylphenol	$C_{17}H_{28}O$	8.27	248.21	1.63×10^{6}	1.73×10^{6}	1.34×10^{6}	8.67×10^{5}	5.87×10^{5}	6.41×10^{5}	0.45	-1.17	4.09×10^{-3}	1.00	1.77	down	Suspected contaminant
C1487	2,2-Methylenebis(6-cyclohexyl-4-methylphenol)	$C_{27}H_{36}O_2$	7.91	392.27	1.36×10^{6}	1.12×10^{6}	1.27×10^{6}	7.37×10^{5}	5.52×10^{5}	7.28×10^{5}	0.54	-0.89	3.57×10^{-3}	1.00	1.81	down	Suspected contaminant
C1575	albocycline	$C_{18}H_{28}O_4$	6.71	308.20	1.62×10^{6}	1.97×10^{6}	2.25×10^{6}	1.51×10^{6}	1.01×10^{6}	1.34×10^{6}	0.66	-0.60	4.59×10^{-2}	1.00	1.49	down	Confident compound
C1576	Cer 18:1;2O/20:3	$C_{38}H_{69}NO_3$	12.31	587.53	1.60×10^{6}	1.27×10^{6}	8.23×10^{5}	2.47×10^{5}	1.87×10^{5}	1.65×10^{5}	0.16	-2.63	1.06×10^{-2}	1.00	1.74	down	Confident compound
C1613	3,9-Bis[(11-methyldodecyl)oxy]-2,4,8,10-tetraoxa-3,9-diphosphaspiro[5.5]undecane	$C_{31}H_{62}O_6P_2$	8.20	592.40	2.35×10^{6}	1.51×10^{6}	2.03×10^{6}	6.81×10^{5}	7.74×10^{5}	1.78×10^{5}	0.28	-1.85	9.77×10^{-3}	1.00	1.76	down	Suspected contaminant
C1633	6-Gingerol	$C_{17}H_{26}O_4$	5.76	294.18	1.21×10^{6}	1.34×10^{6}	1.43×10^{6}	5.87×10^{5}	9.75×10^{5}	7.12×10^{5}	0.57	-0.80	1.26×10^{-2}	1.00	1.71	down	Confident compound
C1716	Sulfolithocholic acid	$C_{24}H_{40}O_6S$	10.66	456.25	2.83×10^{7}	2.50×10^{7}	1.42×10^{7}	5.23×10^{6}	9.57×10^{6}	7.05×10^{6}	0.32	-1.63	2.64×10^{-2}	1.00	1.65	down	Confident compound
C1747	5-[5-(methoxycarbonyl)-5,8a-dimethyl-2-methylidene-decahydronaphthalen-1-yl]-3-methylpentanoic acid	$C_{21}H_{34}O_4$	5.41	350.25	4.67×10^{5}	4.54×10^{5}	4.71×10^{5}	1.59×10^{5}	1.64×10^{5}	3.67×10^{5}	0.50	-1.01	2.72×10^{-2}	1.00	1.64	down	Confident compound
C1815	GlcCer(d18:1/16:0)	$C_{40}H_{77}NO_8$	9.85	699.57	7.74×10^{5}	8.04×10^{5}	9.24×10^{5}	3.93×10^{5}	5.04×10^{5}	6.18×10^{5}	0.61	-0.72	1.43×10^{-2}	1.00	1.71	down	Confident compound
C1900	7α-Hydroxytestosterone	$C_{19}H_{28}O_3$	4.94	304.20	4.80×10^{5}	5.19×10^{5}	8.67×10^{5}	1.74×10^{5}	1.61×10^{5}	2.84×10^{5}	0.33	-1.59	3.21×10^{-2}	1.00	1.59	down	Confident compound
C2011	Diisononyl adipate	$C_{24}H_{46}O_4$	9.85	398.34	4.10×10^{7}	3.45×10^{7}	2.15×10^{7}	1.41×10^{7}	9.50×10^{6}	1.40×10^{7}	0.39	-1.37	2.89×10^{-2}	1.00	1.63	down	Suspected contaminant
C2017	Succinic acid	$C_4H_6O_4$	0.83	118.03	7.41×10^{8}	1.14×10^{9}	1.77×10^{9}	5.52×10^{7}	2.37×10^{8}	7.85×10^{7}	0.10	-3.30	2.31×10^{-2}	1.00	1.62	down	Confident compound
C2091	PI-Cer 12:0;2O/23:0;O	$C_{41}H_{82}NO_{12}P$	13.07	811.56	1.23×10^{8}	1.22×10^{8}	9.68×10^{7}	3.44×10^{7}	4.77×10^{7}	2.97×10^{7}	0.33	-1.61	1.61×10^{-3}	1.00	1.83	down	Confident compound
C2121	1,3-Dioxolane	$C_3H_6O_2$	0.84	74.04	1.79×10^{7}	2.40×10^{7}	3.78×10^{7}	1.34×10^{6}	4.90×10^{6}	2.62×10^{6}	0.11	-3.17	1.68×10^{-2}	1.00	1.66	down	Suspected contaminant
C2163	D&C Red Dye 27	$C_{20}H_4Br_4Cl_4O_5$	11.37	779.56	2.70×10^{7}	4.13×10^{7}	2.52×10^{7}	9.32×10^{6}	1.56×10^{6}	9.89×10^{6}	0.22	-2.17	1.35×10^{-2}	1.00	1.66	down	Suspected contaminant
C2170	PI-Cer 12:0;2O/21:0;O	$C_{39}H_{78}NO_{12}P$	13.08	783.53	6.12×10^{7}	4.54×10^{7}	3.93×10^{7}	1.25×10^{7}	2.34×10^{7}	8.87×10^{6}	0.31	-1.70	1.26×10^{-2}	1.00	1.74	down	Confident compound
C2205	4-Hydroxy-3-methoxyphenylglycol sulfate	$C_9H_{12}O_7S$	3.28	264.03	1.97×10^{6}	6.09×10^{5}	1.26×10^{6}	7.43×10^{4}	1.46×10^{5}	4.91×10^{4}	0.07	-3.83	3.94×10^{-2}	1.00	1.63	down	Confident compound
C2206	3-[3-(Sulfooxy)phenyl]propanoic acid	$C_9H_{10}O_6S$	3.28	246.02	1.97×10^{6}	6.26×10^{5}	1.26×10^{6}	8.38×10^{4}	1.46×10^{5}	5.53×10^{4}	0.07	-3.76	3.79×10^{-2}	1.00	1.64	down	Confident compound
C2223	BTB13021	$C_{12}H_{16}N_2S$	0.51	220.11	2.22×10^{7}	3.47×10^{7}	2.40×10^{7}	1.02×10^{7}	7.43×10^{6}	1.34×10^{7}	0.38	-1.39	1.75×10^{-2}	1.00	1.63	down	Confident compound

ID	Description	Formula	RT (min)	m/z	Model1	Model2	Model3	DCDPE1	DCDPE2	DCDPE3	FC	log2FC	P value	ROC	VIP	Up/Down	Annotation category
C2245	NAGlySer 15:0/17:0	$C_{37}H_{70}N_2O_7$	12.51	654.52	3.22×10^{7}	3.02×10^{7}	2.00×10^{7}	1.01×10^{7}	1.02×10^{7}	1.16×10^{7}	0.39	-1.37	1.15×10^{-2}	1.00	1.73	down	Confident compound
C2266	NAGlySer 15:0/16:0	$C_{36}H_{68}N_2O_7$	12.01	640.50	3.63×10^{7}	2.71×10^{7}	2.47×10^{7}	1.24×10^{7}	1.29×10^{7}	8.44×10^{6}	0.38	-1.38	9.10×10^{-3}	1.00	1.76	down	Confident compound
C2283	IPIKCTWGPWLVBN-FGYSCDCISA-N	$C_{30}H_{44}O_5$	6.94	484.32	6.56×10^{6}	1.26×10^{7}	1.52×10^{7}	2.18×10^{6}	4.34×10^{6}	3.49×10^{6}	0.29	-1.78	3.71×10^{-2}	1.00	1.54	down	Confident compound
C2302	HMHFWDKFVGYHMA-UHFFFAOYSA-N	$C_{30}H_{44}O_7$	5.55	516.31	7.79×10^{6}	7.10×10^{6}	1.07×10^{7}	3.17×10^{6}	2.83×10^{6}	2.90×10^{6}	0.35	-1.52	7.28×10^{-3}	1.00	1.75	down	Confident compound
C2336	PI-Cer 12:0;2O/24:0;O	$C_{42}H_{84}NO_{12}P$	13.06	825.57	1.80×10^{7}	2.16×10^{7}	1.86×10^{7}	1.03×10^{6}	6.02×10^{6}	4.51×10^{5}	0.13	-2.95	1.28×10^{-3}	1.00	1.82	down	Confident compound
C2391	LNAPE 22:0/N-2:0	$C_{29}H_{58}NO_8P$	9.26	579.39	4.28×10^{6}	2.80×10^{6}	4.69×10^{6}	1.12×10^{6}	1.16×10^{6}	1.51×10^{6}	0.32	-1.64	1.05×10^{-2}	1.00	1.76	down	Confident compound
C2454	SL 18:1;O/15:0	$C_{33}H_{65}NO_5S$	9.66	587.46	5.29×10^{6}	4.94×10^{6}	3.65×10^{6}	2.76×10^{6}	2.44×10^{6}	1.19×10^{6}	0.46	-1.12	2.27×10^{-2}	1.00	1.64	down	Confident compound
C2458	PE O-24:6_16:0	$C_{45}H_{80}NO_7P$	11.40	777.56	5.29×10^{6}	5.71×10^{6}	3.83×10^{6}	2.27×10^{6}	3.48×10^{6}	3.33×10^{6}	0.61	-0.71	4.92×10^{-2}	1.00	1.54	down	Confident compound
C2509	LNAPE 15:0/N-2:0	$C_{22}H_{44}NO_8P$	6.66	481.28	7.39×10^{6}	5.13×10^{6}	7.72×10^{6}	2.39×10^{6}	1.86×10^{6}	3.34×10^{6}	0.37	-1.42	1.01×10^{-2}	1.00	1.76	down	Confident compound
C2534	HXHLXXMORMBTQM-UHFFFAOYSA-N	$C_{30}H_{46}O_7$	4.95	518.32	5.03×10^{6}	6.47×10^{6}	4.79×10^{6}	1.02×10^{6}	2.85×10^{6}	1.77×10^{6}	0.35	-1.53	9.02×10^{-3}	1.00	1.73	down	Confident compound
C2553	Hexadecanedioic acid	$C_{16}H_{30}O_4$	5.42	286.21	4.37×10^{6}	5.24×10^{6}	6.38×10^{6}	2.63×10^{6}	3.60×10^{6}	3.86×10^{6}	0.63	-0.66	4.72×10^{-2}	1.00	1.53	down	Confident compound
C2585	Linoleic acid-biotin	$C_{28}H_{48}N_4O_3S$	4.81	520.34	3.07×10^{6}	3.27×10^{6}	1.48×10^{6}	4.18×10^{5}	8.16×10^{5}	7.98×10^{5}	0.26	-1.94	2.94×10^{-2}	1.00	1.62	down	Suspected contaminant
C2661	NAGlySer 14:0/16:0	$C_{35}H_{66}N_2O_7$	11.58	626.49	7.58×10^{6}	5.34×10^{6}	3.86×10^{6}	2.04×10^{6}	2.39×10^{6}	1.42×10^{6}	0.35	-1.52	3.10×10^{-2}	1.00	1.64	down	Confident compound
C2708	LPE O-18:1	$C_{23}H_{48}NO_6P$	8.26	465.32	2.27×10^{6}	3.26×10^{6}	2.49×10^{6}	9.77×10^{5}	1.59×10^{6}	1.35×10^{6}	0.49	-1.03	1.72×10^{-2}	1.00	1.65	down	Confident compound
C2726	NP-017624	$C_{23}H_{32}O_8$	4.31	436.21	2.14×10^{6}	2.16×10^{6}	2.90×10^{6}	1.63×10^{6}	1.01×10^{6}	5.78×10^{5}	0.45	-1.16	2.81×10^{-2}	1.00	1.59	down	Confident compound
C2739	N-(4-Fluorobenzyl)-2-[(1S,4S,5S)-5-isopropyl-2-methyl-4-{[(propylsulfonyl)amino]methyl}-2-cyclohexen-1-yl]acetamide	$C_{23}H_{35}FN_2O_3S$	4.16	438.23	1.35×10^{6}	1.08×10^{6}	1.28×10^{6}	4.40×10^{5}	9.00×10^{5}	7.62×10^{5}	0.57	-0.82	2.72×10^{-2}	1.00	1.68	down	Suspected contaminant
C2779	Acoric acid	$C_{15}H_{24}O_4$	5.47	268.17	2.37×10^{6}	2.21×10^{6}	1.99×10^{6}	8.61×10^{5}	8.68×10^{5}	3.15×10^{5}	0.31	-1.68	2.11×10^{-3}	1.00	1.81	down	Confident compound
C2840	(+/-)-C75	$C_{14}H_{22}O_4$	5.39	254.15	2.35×10^{6}	1.80×10^{6}	2.03×10^{6}	1.11×10^{6}	1.38×10^{6}	1.09×10^{6}	0.58	-0.79	9.23×10^{-3}	1.00	1.78	down	Suspected contaminant
C2877	Myristic acid alkyne	$C_{14}H_{24}O_2$	6.25	224.18	3.54×10^{6}	2.43×10^{6}	1.51×10^{6}	5.22×10^{5}	7.15×10^{5}	1.02×10^{5}	0.18	-2.48	2.88×10^{-2}	1.00	1.65	down	Suspected contaminant
C2895	PE O-16:4_15:0	$C_{36}H_{66}NO_7P$	12.41	655.45	2.23×10^{6}	1.84×10^{6}	2.07×10^{6}	4.84×10^{5}	2.75×10^{5}	4.07×10^{5}	0.19	-2.40	2.21×10^{-4}	1.00	1.87	down	Confident compound
C2981	SHexCer 42:0;3O	$C_{48}H_{95}NO_{12}S$	11.34	909.66	2.14×10^{6}	2.25×10^{6}	2.50×10^{6}	1.20×10^{6}	1.27×10^{6}	1.05×10^{6}	0.51	-0.97	7.79×10^{-4}	1.00	1.83	down	Confident compound
C3037	2,4,8,10-Tetraoxaspiro[5.5]undecane-3,9-diylbis-2-methyl-2,1-propanediyl bis{3-[4-hydroxy-3-methyl-5-(2-methyl-2-propanyl)phenyl]propanoate}	$C_{43}H_{64}O_{10}$	9.50	740.45	2.36×10^{6}	2.18×10^{6}	1.90×10^{6}	3.20×10^{5}	4.19×10^{5}	3.77×10^{5}	0.17	-2.53	2.09×10^{-4}	1.00	1.87	down	Suspected contaminant
C3056	LPI 20:3	$C_{29}H_{51}O_{12}P$	6.68	622.31	9.42×10^{5}	6.76×10^{5}	8.46×10^{5}	2.45×10^{5}	2.83×10^{5}	1.37×10^{5}	0.27	-1.89	2.57×10^{-3}	1.00	1.83	down	Confident compound
C3082	Cer 17:0;2O/16:0;(3OH)	$C_{33}H_{67}NO_4$	11.71	541.51	1.40×10^{6}	8.50×10^{5}	8.95×10^{5}	3.10×10^{5}	4.39×10^{5}	3.32×10^{5}	0.34	-1.54	1.86×10^{-2}	1.00	1.72	down	Confident compound
C3204	Cer 17:0;2O/15:0;(3OH)	$C_{32}H_{65}NO_4$	11.29	527.49	8.34×10^{5}	5.31×10^{5}	5.89×10^{5}	1.68×10^{5}	1.44×10^{5}	1.63×10^{5}	0.24	-2.04	6.08×10^{-3}	1.00	1.80	down	Confident compound
